# Strain Prioritization and Genome Mining for Enediyne Natural Products

**DOI:** 10.1128/mBio.02104-16

**Published:** 2016-12-20

**Authors:** Xiaohui Yan, Huiming Ge, Tingting Huang, Hindra, Dong Yang, Qihui Teng, Ivana Crnovčić, Xiuling Li, Jeffrey D. Rudolf, Jeremy R. Lohman, Yannick Gansemans, Xiangcheng Zhu, Yong Huang, Li-Xing Zhao, Yi Jiang, Filip Van Nieuwerburgh, Christoph Rader, Yanwen Duan, Ben Shen

**Affiliations:** a Department of Chemistry, the Scripps Research Institute, Jupiter, Florida, USA; b Department of Cancer Biology, the Scripps Research Institute, Jupiter, Florida, USA; c Laboratory of Pharmaceutical Biotechnology, Ghent University, Ghent, Belgium; d Xiangya International Academy of Translational Medicine, Central South University, Changsha, Hunan, China; e Hunan Engineering Research Center of Combinatorial Biosynthesis and Natural Product Drug Discovery, Changsha, Hunan, China; f Yunnan Institute of Microbiology, Yunnan University, Kunming, Yunnan, China; g Department of Molecular Therapeutics, the Scripps Research Institute, Jupiter, Florida, USA; h Natural Products Library Initiative at The Scripps Research Institute, the Scripps Research Institute, Jupiter, Florida, USA

## Abstract

The enediyne family of natural products has had a profound impact on modern chemistry, biology, and medicine, and yet only 11 enediynes have been structurally characterized to date. Here we report a genome survey of 3,400 actinomycetes, identifying 81 strains that harbor genes encoding the enediyne polyketide synthase cassettes that could be grouped into 28 distinct clades based on phylogenetic analysis. Genome sequencing of 31 representative strains confirmed that each clade harbors a distinct enediyne biosynthetic gene cluster. A genome neighborhood network allows prediction of new structural features and biosynthetic insights that could be exploited for enediyne discovery. We confirmed one clade as new C-1027 producers, with a significantly higher C-1027 titer than the original producer, and discovered a new family of enediyne natural products, the tiancimycins (TNMs), that exhibit potent cytotoxicity against a broad spectrum of cancer cell lines. Our results demonstrate the feasibility of rapid discovery of new enediynes from a large strain collection.

## IMPORTANCE

Recent advances in microbial genomics clearly revealed that the biosynthetic potential of soil actinomycetes to produce enediynes is underappreciated. A great challenge is to develop innovative methods to discover new enediynes and produce them in sufficient quantities for chemical, biological, and clinical investigations. This work demonstrated the feasibility of rapid discovery of new enediynes from a large strain collection. The new C-1027 producers, with a significantly higher C-1027 titer than the original producer, will impact the practical supply of this important drug lead. The TNMs, with their extremely potent cytotoxicity against various cancer cells and their rapid and complete cancer cell killing characteristics, in comparison with the payloads used in FDA-approved antibody-drug conjugates (ADCs), are poised to be exploited as payload candidates for the next generation of anticancer ADCs. Follow-up studies on the other identified hits promise the discovery of new enediynes, radically expanding the chemical space for the enediyne family.

## INTRODUCTION

Natural products offer unmatched chemical and structural diversity compared to any other small-molecule families. They continue to inspire novel chemistry, enzymology, and biology investigations and remain the best sources of drugs and drug leads (1). The enediynes represent one of the most fascinating families of natural products for their unprecedented molecular architecture and extraordinary biological activities. Since the neocarzinostatin (NCS) chromophore structure was first unveiled in 1985 (2), the enediyne family has grown steadily but remains very small, with only 11 structurally characterized members and 4 additional members isolated in their cycloaromatized form known to date. Classified into two subcategories according to the size of the enediyne core structures (3–7), members of the 9-membered enediyne subcategory include NCS, C-1027, kedarcidin (KED), maduropeptin (MDP), N1999A2, the sporolides (SPO), the cyanosporasides (CYA and CYN), and the fijiolides, with the latter four isolated in cycloaromatized form (see Fig. S1A in the supplemental material). Members of the 10-membered enediyne subcategory include the calicheamicins (CAL), the esperamicins (ESP), dynemicin (DYN), namenamicin, shishijimicin, and uncialamycin (UCM) (Fig. S1B).

The enediynes have had a profound impact on modern chemistry, biology, and medicine (3, 4, 7). All enediynes contain a unit consisting of two acetylenic groups conjugated to a double bond or an incipient double bond within the 9- or 10-membered carbocycle (Fig. S1A and B). As a consequence of this structural feature, the enediynes share a mode of action—electronic rearrangement of the enediyne carbocycle produces a transient benzenoid diradical. When positioned within the minor groove of DNA, the diradical abstracts hydrogen atoms from the deoxyribose backbone of duplex DNA; the DNA-centered radicals can then cause interstrand cross-links (ICLs) or react with molecular oxygen, leading ultimately to DNA double-strand breaks (DSBs), or both (3, 4, 7, 8). With their exquisite mode of action and their extraordinary cytotoxicity, the enediynes have been successfully translated into clinical drugs. It is remarkable that, among the 11 enediynes known to date, 2 [NCS as poly(styrene-comaleic acid)-conjugated NCS (SMANCS) and CAL as gemtuzumab ozogamicin (Mylotarg)] have been developed into marketed drugs, 1 (C-1027) is in clinical trials, and another (UCM) is in preclinical studies, representing an astonishing and remarkable ~35% success rate with the enediyne class of natural products (3–7). A great challenge is that of developing innovative methods to discover new enediynes and producing them in sufficient quantities for chemical, biological, and clinical investigations.

Here we report strain prioritization and genome mining for enediyne natural products from the actinomycetes strain collection at the Scripps Research Institute (TSRI). By surveying 3,400 strains, we identified 81 potential producers, and genome sequencing of 31 representatives revealed at least 28 distinct enediyne biosynthetic gene clusters. We constructed an enediyne genome neighborhood network (GNN) to facilitate gene cluster annotation and to predict new structural features, thereby further streamlining the discovery of new enediyne natural products. To demonstrate the feasibility of our approach in rapidly discovering new enediynes from a large strain collection, we characterized a new C-1027 producer with a significantly higher C-1027 titer than the original *Streptomyces globisporus* producer (9, 10) and discovered the tiancimycins (TNMs), new enediyne natural products that exhibit potent cytotoxicity against a broad spectrum of cancer cell lines and kill selected cancer cells more rapidly and completely than the payloads used in FDA-approved antibody (Ab)-drug conjugates (ADCs). Production of TNMs in sufficient quantities by microbial fermentation and manipulation of TNM biosynthesis for engineering new analogues were also demonstrated.

## RESULTS AND DISCUSSION

### Genome survey of 3,400 actinomycetes for the enediyne polyketide synthase gene cassette identifying 81 potential enediyne producers that could be grouped into 28 distinct clades.

Since the cloning of the first 9-membered (C-1027) (11) and 10-membered (CAL) (12) enediyne biosynthetic gene clusters in 2002, three additional 9-membered (NCS, MDP, and KED) (13–15) and two additional 10-membered (DYN and ESP [partial]) (16, 17) enediyne gene clusters, as well as the three clusters encoding the biosynthesis of SPO (18), CYA (19), and CYN (19), have been characterized. Comparative analysis of the 10 gene clusters revealed a set of 5 genes common to all enediynes, i.e., the enediyne polyketide synthase (PKS) gene cassette consisting of *E3*, *E4*, *E5*, *E*, and *E10* (*E3*/*E4*/*E5*/*E*/*E10*) (Fig. S2A) (17, 20–22); no apparent conservation was observed beyond the enediyne PKS gene cassettes, accounting for the structural diversity characteristic for the periphery moieties of the enediynes (Fig. S1A and B). The remarkable sequence homology prompted us to select genes within the enediyne PKS cassettes as probes to survey genomes for the presence of enediyne biosynthetic machinery. This was validated recently by a virtual survey of all bacterial genomes available in public databases, revealing the rich potential of biosynthesis of enediynes by actinomycetes (Fig. S2B and C) (5, 6).

**FIG 1:**
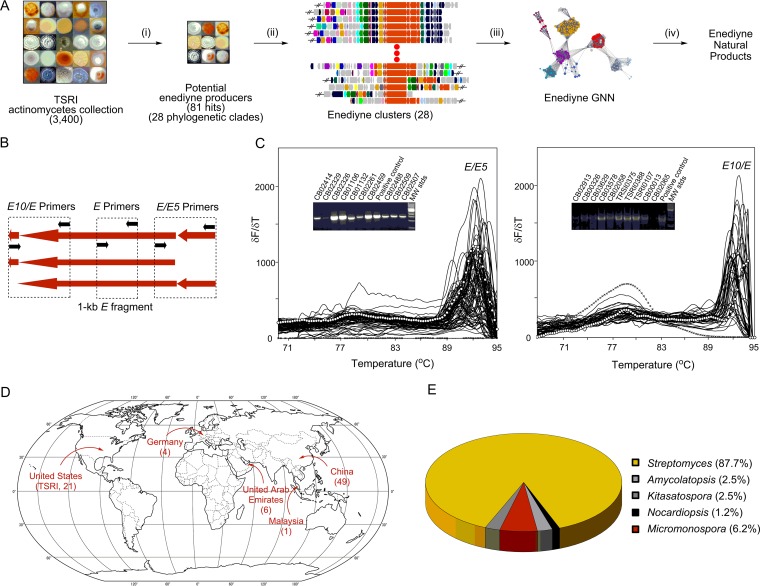
Strain prioritization and genome mining for enediyne natural products. (A) A high-throughput method to survey the enediyne biosynthetic machinery in a strain collection and prioritize the hits for new enediyne natural-product discovery: (i) genome survey of 3,400 representative strains from the TSRI actinomycetes collection identifying 81 novel enediyne producers; (ii) genome sequencing of 31 representative producers yielding 28 distinct enediyne biosynthetic gene clusters; (iii) construction of an enediyne GNN unveiling new insights for enediyne biosynthesis and structural novelty; and (iv) fermentation optimization and production, isolation, and structural elucidation affording the new enediyne natural products. (B) Design of PCR primers for enediyne PKS gene cassette, targeting *E5*/*E* or *E*/*E10*, and the primers for the 1-kb internal fragment of *E*. (C) Representative melting curve analysis in real-time PCR in a 384-well plate format, as exemplified by using *E*/*E5* or *E10*/*E* primers, with each of the peaks indicating a specific product. Solid lines with open circles represent the positive controls (genomic DNAs of the C-1027 producer *S. globisporus* and the KED producer *Streptomyces* sp. strain ATCC 53650 were used as positive controls for *E*/*E5* and *E10*/*E* amplification, respectively), and dashed lines represent the negative controls with no-template DNA. Insets show PCR products of the hits that were analyzed by agarose gel electrophoresis and confirmed by DNA sequencing. (D) Geographic distribution of the 81 new enediyne producers identified (see Table S2 for details). Numbers in parentheses are the numbers of new enediyne producers isolated from each of the clades. (E) Taxonomic distribution of the 81 newly identified enediyne producers in the culture collection, with approximately 88% of them belonging to the *Streptomyces* genus, ensuring that the expedient genetic tools developed in the past two decades for *Streptomyces* will be readily available to manipulate the enediyne biosynthetic machinery in these producers for production, titer improvement, and structural diversity.

**FIG 2:**
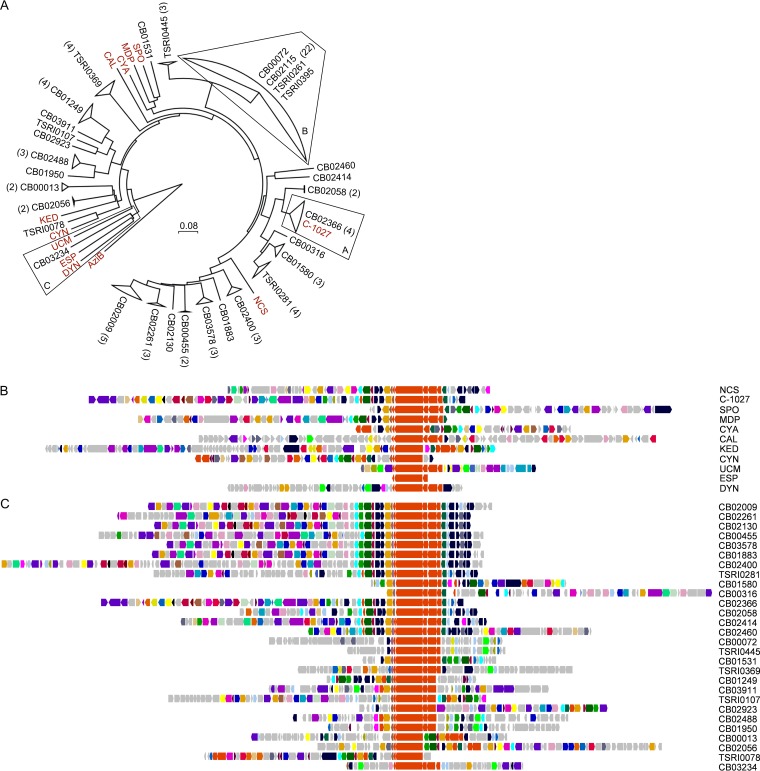
A genome survey of 3,400 strains from the TSRI actinomycetes collection, identifying 81 enediyne producers. (A) Phylogenetic analysis of the 81 enediyne gene clusters, based on the translated 1-kb internal fragment of the *E* genes, in comparison with the 11 known enediyne producers (red), affording 28 distinct clades upon employing a 90% amino acid identity cutoff value. Numbers in parentheses are the hits identified from each of the clades. The clades of alternative C-1027 producers (section A), multiple producers sequenced with highly homologous enediyne gene clusters (section B), and the TNM producer *Streptomyces* sp. strain CB03234 (section C) are highlighted. (B) The 10 enediyne biosynthetic gene clusters known previously and the *ucm* biosynthetic gene cluster characterized in this study (see Table S3 for detailed annotation). (C) The 28 distinct enediyne gene clusters identified upon genome sequencing of 31 representative hits from the 28 clades (see Tables S3 and S4 for detailed annotation). Genes are color-coded based on GNN annotation (see Fig. 4).

Inspired by the accuracy and specificity of the virtual screening, we adapted our recently developed high-throughput real-time PCR method (23) to survey the TSRI actinomycetes collection for the enediyne PKS gene cassettes to identify new enediyne producers (Fig. 1A). Two sets of PCR primers were designed to specifically target *E5*/*E* or *E*/*E10* (Fig. 1B). Hits identified by both sets of the primers featured the enediyne PKS gene cassettes with *E5*/*E*/*E10* clustered together, while hits identified by only one of the two sets of primers featured an enediyne PKS gene cassette with either *E5* or *E10* separate from the *E* gene (Fig. 1B). By real-time PCR in a 384-well plate format, specific PCR products were rapidly identified, in a high-throughput manner, by melting curve analysis and confirmed by gel electrophoresis and DNA sequencing (Fig. 1C). From 3,400 representative strains, 81 distinct enediyne producers were identified on the basis of the identity of the enediyne PKS gene cassettes (i.e., *E5*, *E10*, and a 1-kb internal fragment of *E*), taxonomy, and geographic locations where the strains were isolated (Fig. 1B to E). Phylogenetic analysis of the 81 new enediyne PKS cassettes, with the known enediyne PKS cassettes as controls, was carried out using the translated 1-kb internal fragment of *E*. While each of the enediyne PKS cassettes is unique, the phylogenetic tree of the 81 new enediyne PKS cassettes subjected to 90% amino acid identity cutoff collapsed into 28 distinct clades (the pairwise comparison of the known enediyne PKS cassettes revealed amino acid sequence identities ranging from 33% to 69%) (Fig. 2A). It is therefore very significant that 27 of the 28 clades are distinct from the known enediyne PKS cassettes, indicative of novel enediynes. The CB02366 group forms a clade with the C-1027 enediyne PKS cassette (11), suggesting that these hits potentially represent new C-1027 producers (Fig. 2A, section A).

Genome sequencing of 31 representative hits from the 28 clades confirmed that they all contain an enediyne gene cluster and therefore are true enediyne producers (Fig. 2). Significantly, hits from different clades yielded distinct enediyne gene clusters, while hits from the same clade afforded highly homologous gene clusters, as exemplified by TSRI0395, TSRI0261, CB02115, and CB00072 (Fig. 2A, section B) (only the cluster from CB00072 is shown; Fig. 2C). These findings support the proposal of using the enediyne PKS cassette clades to further prioritize the hits and streamline enediyne natural-product dereplication.

### Discovery of *Streptomyces* sp. strain CB02366 as a new C-1027 producer with a significantly higher titer than the original producer.

**FIG 3:**
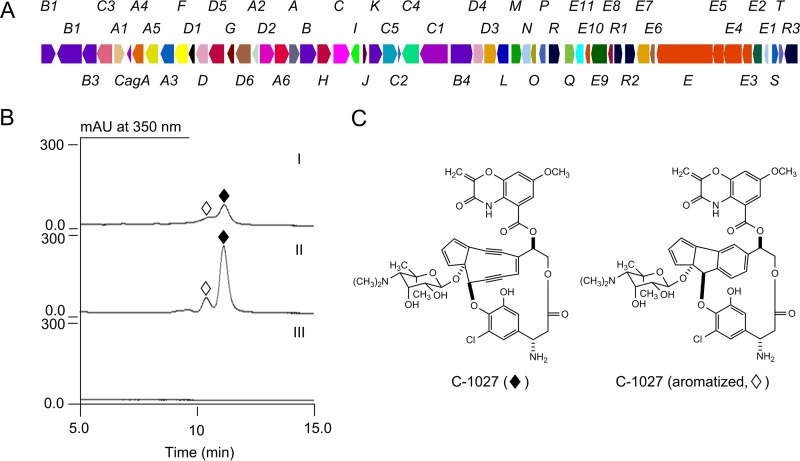
Characterization of *Streptomyces* sp. strain CB02366 as an alternative C-1027 producer. (A) Genetic organization of the C-1027 gene cluster from CB02366 (see Table S3). (B) HPLC analysis of fermentations of the *Streptomyces* sp. strain CB02366 wild type (II) and SB1036 (i.e., Δ*pksE*) mutant (III), with the *S. globisporus* wild-type strain as a control (I), establishing CB02366 as a new C-1027 producer with a significantly higher C-1027 (solid diamonds) titer. Titers for C-1027 chromoprotein production were estimated on the basis of the C-1027 chromophore as determined by HPLC analysis. The *S. globisporus* wild-type strain produces ~5.5 mg/liter of the C-1027 chromophore, which translates to ~74 mg/liter of the C-1027 chromoprotein (9). mAU, milli-absorbance units. (C) The structures of C-1027 (solid diamonds) and its aromatized metabolite (open diamonds).

C-1027 was originally isolated from *Streptomyces globisporus* in 1993 (10). C-1027 has been in clinical development as an antibody-drug conjugate (ADC) for hepatoma (24, 25). We have been studying C-1027 biosynthesis in *S. globisporus* as a model for enediyne biosynthesis and engineering (7, 11, 20, 22, 26). To confirm the four hits that form a clade with the C-1027 enediyne PKS cassette as alternative C-1027 producers (Fig. 2A, section A), we sequenced the genome of *Streptomyces* sp. strain CB02366, a representative from the clade, and indeed revealed a C-1027 biosynthetic gene cluster (Fig. 3A). We subsequently isolated C-1027 from *Streptomyces* sp. strain CB02366 and generated *ΔpksE* mutant strain SB1036 (Fig. S3; see also Table S1 in the supplemental material), which has completely lost its ability to produce C-1027, thereby confirming that the cloned gene cluster encodes the C-1027 biosynthetic machinery (Fig. 3B and C). Interestingly, CB02366, isolated from a soil sample collected in Dubai, United Arab Emirates, is classified as a *Streptomyces griseus* species (Table S2), but the original C-1027 producer, isolated from a soil sample collected in Qianjiang county, Hubei Province, China, is known as *Streptomyces globisporus* (27). And yet the two C-1027 clusters are highly homologous (with identical genetic organizations and ~90%/94% identity of DNA/amino acid sequences) (Fig. 3A and Table S3). The finding of *Streptomyces* sp. strain CB02366 as an alternative C-1027 producer is significant because C-1027 had not been rediscovered since it was first isolated in 1993 (10). Remarkably, *Streptomyces* sp. strain CB02366 produces the C-1027 chromoprotein with a titer of ~750 mg/liter, which is minimally 10-fold higher than that produced by the original *S. globisporus* wild-type strain at ~74 mg/liter (calculated on the basis of 5.5 mg/liter of the C-1027 chromophore as determined by high-performance liquid chromatography [HPLC] analysis) (Fig. 3B) (9). Multiple producers (for either known or new enediynes) with various growth characteristics and levels of genetic amenability also present new opportunities for yield improvement, structural diversity, or both by applying metabolic pathway engineering strategies and methods (1, 28). The fact that most of the new enediyne producers identified in this study are *Streptomyces* species (Fig. 1E) ensures that the expedient recombinant DNA technologies and genetic tools available can be readily adopted for these experiments.

### An enediyne GNN facilitating gene cluster annotation and predicting new structural features.

**FIG 4:**
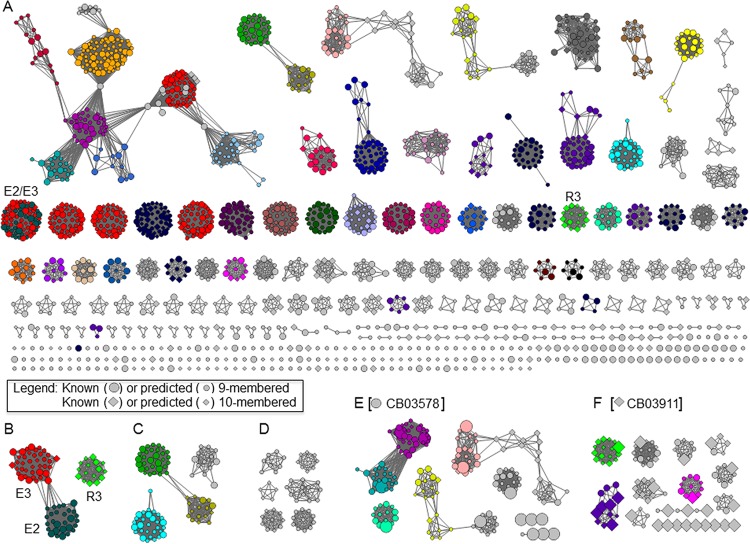
An enediyne GNN revealing that the enediyne biosynthetic gene clusters discovered are rich in new chemistry and diverse structures. (A) The enediyne GNN consisting of the known and new enediyne gene clusters (see Fig. 2B and C) displayed with an *E* value threshold of 10^−6^. The enediyne GNN displays both the conserved nature and the diverse nature of proteins involved in enediyne biosynthesis. (B) The E2, E3, and R3 families displayed with an *E* value threshold of 10^−75^ to separate the E2 and E3 subfamilies for use as 9-membered versus 10-membered enediyne prediction. (C) Selected representatives of proteins of unknown function, highlighting the vast number of uncharacterized and unknown chemistries found in enediyne biosynthesis. (D) Selected representatives of proteins of unknown function found only in the new enediyne gene clusters, representing novel chemistry, enzymology, and structures found in the new enediyne producers. Big and small dots and diamonds in panels A to D denote known and predicted 9- and 10-membered enediynes, respectively. (E) A predicted 9-membered enediyne cluster from CB03578 (denoted by big dots) featuring tryptophan degradation enzymes with corresponding activation and condensing enzymes, uncharacterized protein families, and singletons. (F) A predicted 10-membered enediyne cluster from CB03911 (denoted with big diamonds), with several conserved families of proteins for regulation and resistance in 10-membered biosynthesis and shikimate pathway enzymes and singletons unique to 10-membered enediyne biosynthesis.

For the other 27 clades, genome sequencing of representative hits unveiled gene clusters that are distinct from all enediyne biosynthetic gene clusters known to date (Fig. 2), indicative of novel enediyne natural products. GNNs have recently emerged as a component of a powerful bioinformatics strategy to predict enzyme functions on a large scale based on their genomic context (29, 30). Given the high complexity of enediyne gene clusters (often spanning 80 kb to 120 kb of DNA and consisting of 50 to 75 open reading frames [ORFs]), the large number of functionally unassigned proteins in these gene clusters, and, most importantly, the desire to compare the new enediyne gene clusters to the known ones, we constructed an enediyne GNN to quickly and accurately analyze the new enediyne gene clusters. The enediyne GNN (Fig. 4) included all known enediyne gene clusters (Fig. 2B) and the 31 new enediyne gene clusters (i.e., the 28 distinct gene clusters and the 3 homologous gene clusters from the same clade of CB00072 [Fig. 2A, section B]) (Fig. 2C). The newly discovered enediyne biosynthetic gene clusters are diverse and rich in new chemistry (Fig. 4), featuring many enzyme families that are different from those encoded by the known enediyne gene clusters or are functionally unknown (Fig. 4C and D). Close examination of the GNN unveiled many new insights that could be exploited to predict novel structural features and guide experimental designs to discover new enediyne natural products.

The 28 distinct new gene clusters can be divided into two groups using our recently reported method to predict 9-membered or 10-membered enediynes based on the exclusive presence of *E2* or *R3* genes, respectively (5)—21 clusters expected to encode the production of 9-membered enediynes and 7 clusters expected to encode the production of 10-membered enediynes (Fig. 4B and Table S2). Among the predicted 9-membered producers (Table S2), seven contain apoprotein genes (encoding CB01883, CB02009, CB02130, CB02261, CB02366, CB02400, and CB03578) (also see Table S4), and many strains share common genes responsible for the biosynthesis of peripheral moieties, such as β–amino acids (CB00455, CB01883, CB02009, CB02130, CB02261, and CB02414), amino sugar (CB01580, CB02414, CB02460, and TSRI0369), and benzoxazolinate (CB02261, CB02130, CB02009, CB01883, CB00455, and CB02400) (Table S4). Besides these known moieties, several predicted 9-membered enediyne gene clusters contain genes encoding the biosynthesis of unknown moieties. For example, the enediyne gene cluster from CB03578 encodes enzymes for tryptophan degradation (making those genes unique among enediyne biosynthetic gene clusters), methyltransferases, and corresponding activation and condensing enzymes, implying the presence of an anthranilic acid-like peripheral moiety (Fig. 4E and Table S4).

Among the strains harboring predicted 10-membered enediyne biosynthetic gene clusters (Table S2), one strain (TSRI0369) is proposed to produce an enediyne compound structurally similar to CAL (Table S4) and another one (CB03234) (Table S3) shares many homologous genes with the DYN biosynthetic gene cluster from *Micromonospora chersina* and the UCM gene cluster from *S. uncialis*, while the other five gene clusters (CB00072, TSRI0455, CB03911, CB02488, and CB01950) are fundamentally different from the reported 10-membered enediyne biosynthetic gene clusters (also see Table S4), implying that they might produce new families of 10-membered enediynes. This notion is highlighted by results seen with the gene cluster from CB03911, which possesses putative 10-membered enediyne resistance genes, shikimate pathway enzyme genes, and several singletons (Fig. 4F and Table S4). Chorismate metabolism has been seen in 9-membered enediyne biosynthesis (e.g., benzoxazolinate in C-1027) but was not seen in suspected 10-membered enediyne biosynthesis until now (4, 7, 11).

### Tiancimycins (TNMs) from *Streptomyces* sp. strain CB03234 demonstrating our approach in rapidly discovering new enediynes.

**FIG 5:**
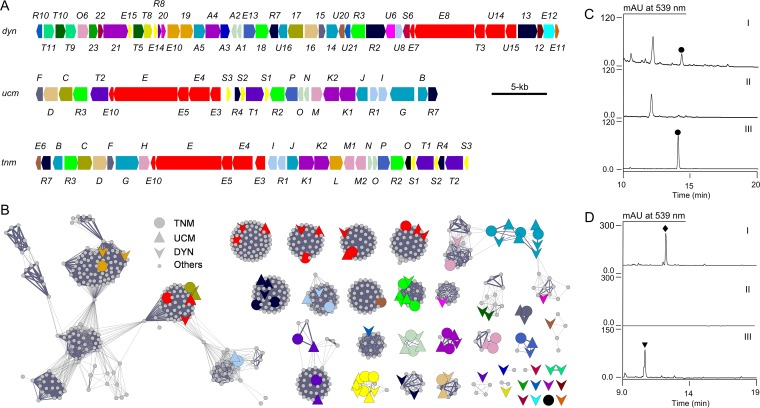
Characterization of *Streptomyces* sp. strain CB03234 as a TNM producer. (A) Genetic organization of the *ucm*, *tnm*, and *dyn* gene clusters (see Table S3). Genes are color-coded based on GNN annotation (see Fig. 4). (B) GNN analysis (*E* value of 10^−6^) unveiling functional similarity among the *dyn*, *ucm*, and *tnm* biosynthetic gene clusters. (C) HPLC analysis of fermentations of the *S. uncialis* wild-type strain (I) and SB18001 (i.e., Δ*ucmE*) mutant (II), in comparison with an authentic UCM standard (III), establishing the cloned gene cluster encoding UCM (solid circles) biosynthesis. (D) HPLC analysis of fermentations of the *Streptomyces* sp. strain CB03234 wild type (I) and the SB20001 (i.e., Δ*tnmE*) (II) and SB20002 (i.e., Δ*tnmH*) (III) mutants, establishing the cloned gene cluster encoding TNM A (solid diamond) biosynthesis and demonstrating the feasibility of manipulating TNM biosynthesis in *Streptomyces* sp. strain CB03234 as exemplified by the engineered production of TNM C (inverted triangle) from SB20002.

We noticed that *Streptomyces* sp. strain CB03234 is clustered together with the UCM (31), ESP (32), and DYN (33) producers upon phylogenetic analysis of their enediyne PKS cassettes (Fig. 2A, section C), suggesting that CB03234 might produce a new 10-membered enediyne. The *dyn* biosynthetic gene cluster was previously cloned from *M. chersina* (Fig. 5A) (16), but the gene cluster for UCM, discovered from *S. uncialis* (31), had not been cloned until now. Thus, we first identified the *ucm* gene cluster by sequencing the *S. uncialis* genome (Fig. 5A and Table S3) and inactivated the *ucmE* gene in *S. uncialis* to generate *ΔucmE* mutant strain SB18001 (Fig. S3 and Table S1). HPLC analysis of SB18001 fermentation, with the *S. uncialis* wild type as a control, showed complete abolishment of UCM production, confirming that the cloned gene cluster encodes UCM biosynthesis (Fig. 5C). We next identified the new enediyne gene cluster (i.e., the *tnm* cluster) from *Streptomyces* sp. strain CB03234 by genome sequencing (Fig. 5A). Remarkably, while the *dyn*, *ucm*, and *tnm* clusters show little conservation in genetic organization beyond the enediyne PKS cassette (Fig. 5A), GNN analysis revealed that the three clusters share many homologous genes (Fig. 5B; also see Table S3) and may therefore encode the biosynthesis of a common enediyne core with various peripheral moieties.

**FIG 6:**
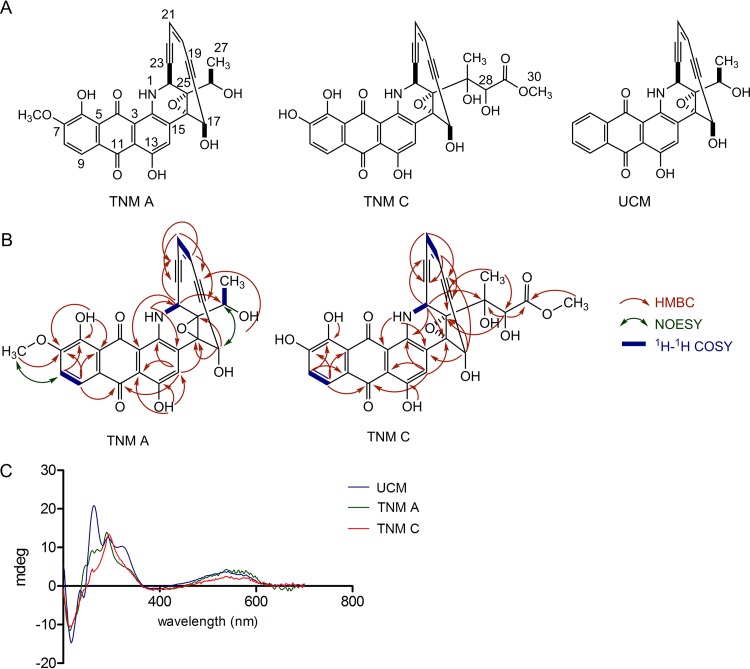
Structural elucidation of TNM A and C on the basis of 1D and 2D NMR and CD spectroscopic data analysis. (A) Structures of TNM A and C in comparison with that of UCM. (B) Key ^1^H-^1^H correlation spectroscopy (COSY), nuclear Overhauser effect spectroscopy (NOESY), and heteronuclear multiple bond correlation (HMBC) analysis of TNM A and C, supporting their structural assignments (also see Table S4). (C) CD spectra of TNM A and C in comparison with an authentic standard of UCM, supporting their absolute stereochemistry assignment.

To discover the new enediyne from *Streptomyces* sp. strain CB03234, we first inactivated the *tnmE* gene in *Streptomyces* sp. strain CB03234 to afford the corresponding *ΔtnmE* mutant strain SB20001 (Fig. S3 and Table S1). Comparison of the HPLC metabolite profiles between fermentations of the *Streptomyces* sp. CB03234 wild-type and SB20001 mutant strains revealed one major metabolite whose biosynthesis could be readily correlated to the *tnm* gene cluster (Fig. 5D). This metabolite was subsequently isolated and named TNM A. The structure of TNM A was established on the basis of extensive mass spectrometry (MS) and one-dimensional (1D) and 2D nuclear magnetic resonance (NMR) analysis (Fig. 6A and B; see also Fig. S4 and Table S5), with its absolute stereochemistry established by comparison of the circular dichroism (CD) spectra of TNM A to an authentic UCM standard (Fig. 6C). TNM A featured an anthraquinone-fused 10-membered enediyne core, characteristic of both DYN and UCM. The DYN producer, isolated from a soil sample collected in Gujarat state, India, was identified as a *M. chersina* species (33). The UCM producer, extracted from the surface of a lichen specimen collected in British Columbia, Canada, was classified as a *S. uncialis* species (31). *Streptomyces* sp. strain CB03234 was isolated from a soil sample collected in Yuanjiang county, Yunnan Province, China, and has been assigned as a *Streptomyces venezuelae* species (Table S2). Pairwise comparison of the *dyn*, *ucm*, and *tnm* clusters, as exemplified by the translated products of the enediyne PKS cassette (i.e., *E10*/*E*/*E5*/*E4*/*E3*; Fig. 5A), revealed only 41% to 60% amino acid sequence identity. It is therefore fascinating that three distantly related strains, harboring gene clusters with distinct organizations, biosynthesize structurally related enediynes, indicative of an intricate evolution for these complex natural-product biosynthetic machineries.

### Manipulation of TNM biosynthesis in *Streptomyces* sp. strain CB03234 demonstrating the feasibility of analogue engineering.

Microbial genome mining and metabolic pathway engineering are rapidly changing the landscape of discovery and structural diversity of natural products (1, 28–30, 34, 35). Development of an expedient genetic system for *in vivo* manipulation of the targeted biosynthetic machinery is of paramount importance to implement these emerging strategies. Thus, a critical decision in manipulating enediyne biosynthesis is the selection of the producers that are compatible with the expedient technologies and tools of recombinant DNA work in *Streptomyces* species and related organisms that have been developed in the past two decades (1, 28, 34, 36, 37). Accordingly, the TSRI actinomycetes collection is enriched with *Streptomyces* species (Fig. 1E), and this selection is vital to overcoming the current challenges of, and meeting future objectives for, enediyne discovery, biosynthesis, and engineering in their native producers.

We have developed an expedient genetic system for *Streptomyces* sp. strain CB03234. The feasibility of manipulating TNM biosynthesis in *Streptomyces* sp. strain CB03234 to generate novel analogues has been demonstrated by the engineered production of a TNM analogue. Thus, we inactivated the *tnmH* gene, encoding a putative *O*-methyltransferase (Fig. 5A and B and Table S3), to generate *ΔtnmH* mutant strain SB20002 (Fig. S3 and Table S1). HPLC analysis of SB20002 fermentation, with the *Streptomyces* sp. strain CB03234 wild type as a control, showed complete abolishment of TNM A production and a concomitant accumulation of a new metabolite (Fig. 5D), which was subsequently identified as TNM C on the basis of extensive MS, CD, and 1D and 2D NMR analysis (Fig. 6; see also Fig. S4 and Table S5). TNM C featured an -OH group at C-7, as would be expected from inactivating the *tnmH* gene, but an additional side chain at C-26, revealing new insights into TNM A biosynthesis. Thus, TnmH-catalyzed O-methylation at C-7 most likely takes place early in TNM A biosynthesis, without which the side chain at C-26 cannot be fully processed *en route* to TNM A as has been proposed previously for UCM biosynthesis (31). Given the biosynthetic relationship among DYN, TNM, and UCM (Fig. 5A and B), manipulation of TNM biosynthesis in CB03234 therefore provides an outstanding bioengineering platform to access the DYN and UCM scaffolds, which have been difficult to access in their native producers due to either recalcitrance to genetic manipulation (for DYN) (16) or the inability to produce in submerged fermentation (for UCM) (31).

### TNMs exhibiting potent cytotoxicity, with rapid and complete killing, toward a broad spectrum of cancer cell lines.

The enediynes are among the most cytotoxic molecules known to date, and they are active in many tumor types. Although the natural enediynes have limited use as clinical drugs, both polymer-based delivery systems and ADCs have shown great clinical success or promise in anticancer therapy (3, 7). For example, poly(styrene-comaleic acid)-conjugated NCS (SMANCS) has been marketed since 1994 for use against hepatoma (38). Several ADCs have been developed, including an anti-CD33 monoclonal antibody (MAb)-CAL conjugate (i.e., gemtuzumab ozogamicin) for acute myeloid leukemia (AML) and an anti-CD22 MAb-CAL conjugate (inotuzumab ozogamicin) for non-Hodgkin lymphoma (39, 40), as well as several MAb-C-1027 conjugates for hepatoma (24) and MAb-UCM conjugates for selected tumors (N. S. Chowdari, S. Gangwar, and B. Sufi, 22 August 2013, European patent application WO 2013122823 A1). These examples demonstrate that the enediynes can be developed into powerful drugs and that the new enediynes therefore represent outstanding payload candidates for ADCs.

We carried out preliminary cytotoxicity evaluations of TNMs in comparison with UCM, auristatin F phenylenediamine (AFP) (41), a variant of the natural product dolostatin 10, and maytansinoid AP-3 (Fig. S1C). Auristatins and maytansinoids are used in the FDA-approved ADCs brentuximab vedotin (Adcetris) and trastuzumab emtansine (Kadcyla), respectively, as well as in many ADCs currently in clinical development (39, 40, 42, 43). The TNMs are extremely potent against a broad spectrum of cancer cell lines, with subnanomolar 50% inhibitory concentrations (IC_50_s). For example, TNM A is more potent than UCM, particularly against breast cancer cell lines (Table 1A); UCM is currently in preclinical development as an ADC (Chowdari et al., European patent application WO 2013122823 A1) (44). Most impressively, as exemplified with the SKBR-3 breast cancer cell line, TNM A exhibited more rapid and more complete cell killing than AFP and AP-3 (Tables 1B and C), thereby minimizing the development of potential drug resistance (45, 46), supporting the wisdom of exploiting the TNMs as payload candidates for the next generation of anticancer ADCs.

**TABLE 1:**
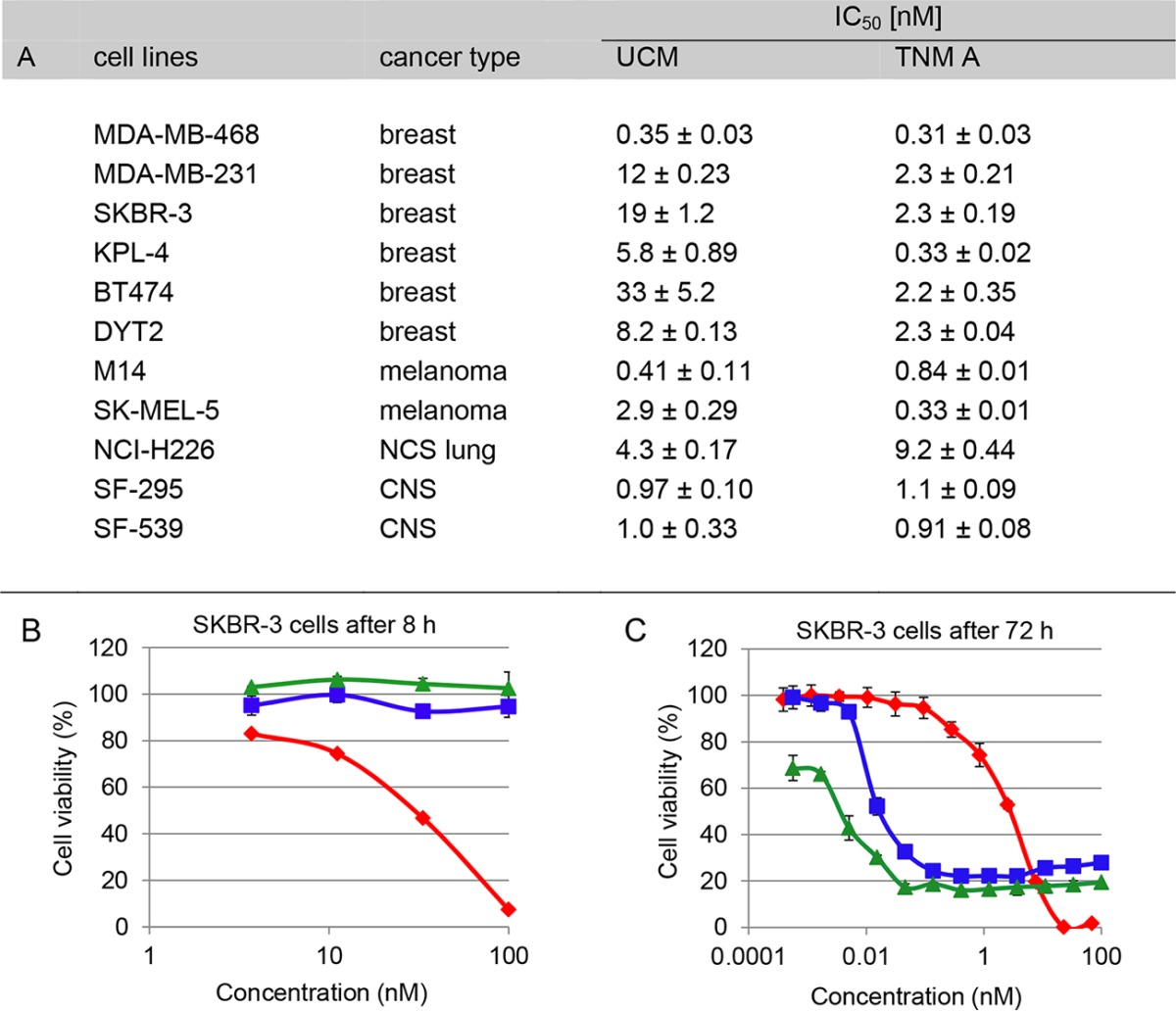
Cytotoxicity of and killing of SKBR-3 cells by TNM A^a^

a (A) Cytotoxicity of TNM A against selected breast and other cancer cell lines in comparison to UCM. (B) Rapid and (C) complete killing of SKBR-3 cells by TNM A (red diamonds) in comparison with AFP (green triangles) and AP-3 (blue squares) (see Fig. S1 for structures), analogues of the natural products auristatin and maytansine used as payloads in the FDA-approved ADCs brentuximab vedotin (Adcetris) and trastuzumab emtansine (Kadcyla), respectively. Each point represents the mean ± SD of results from at least three replicates, and the IC_50_s were determined by computerized curve fitting using GraphPad Prism. ADC, antibody-drug conjugates; TNM, tiancimycin; UCM, uncialamycin.

### Conclusions and significance.

In spite of their profound impact on modern chemistry, biology, and medicine, the enediyne family of natural products remains very small, with only 11 members structurally characterized to date. Recent advances in DNA sequencing and microbial genomics, however, clearly revealed that the biosynthetic potential of soil actinomycetes to produce enediynes is greatly underappreciated. A great challenge is that of developing innovative methods to discover new enediynes and producing them in sufficient quantities for chemical, biological, and clinical investigations. We recently reported a high-throughput real-time PCR method to prioritize strains for natural-product discovery. By adapting this method to identify strains that are highly likely to encode enediyne biosynthesis, followed by genome sequencing, bioinformatics analysis, genetic manipulation, and fermentation optimization, we have now demonstrated the feasibility of rapid discovery of new enediynes from a large strain collection. The new C-1027 producers, with a significantly higher C-1027 titer than the original producer, will impact the practical supply of the drug should it eventually be brought into clinical applications. The TNMs, with their extremely potent cytotoxicity against a broad spectrum of cancer cells and rapid and complete cell killing characteristics, in comparison with the payloads used in FDA-approved ADCs and ADCs in various stages of development, are poised to be exploited as payload candidates for the next generation of anticancer ADCs. Follow-up studies on the other hits that have already been identified in this study or on application of our strategy to other strain collections promise the discovery of new enediynes, radically expanding the chemical space for the enediyne family of natural products. Our results also support strain prioritization and genome mining for the discovery of other classes of natural products from ever-growing microbial strain collections. Together with other emerging strategies and technologies, these findings will inspire continued innovations in natural-product discovery.

## MATERIALS AND METHODS

### General materials.

Primers, plasmids, and strains used and reported in this study are summarized in Table S1 in the supplemental material.

### Construction of the actinomycetes genomic DNA library.

Genomic DNA (gDNA) was prepared from strains isolated from various unexplored and underexplored ecological niches (23, 47, 48). Strains were cultivated in a rich liquid medium (tryptic soy broth [TSB]) for 2 to 3 days. DNA was isolated using the salting-out protocol (36), deposited into 96-well plates, and stored at −80°C.

### Real-time PCR screening of 3,400 strains for enediyne producers.

Real-time PCR was performed using an Applied Biosystems 7900HT Fast real-time PCR system. Preparation of gDNA arrays and application of the real-time PCR method to survey the 3,400 actinomycetes strains for the enediyne PKS gene cassette and to identify new enediyne producers followed the published protocol for strain prioritization with modifications (23). For further details, see Text S1 in the supplemental material.

### Genome sequencing and assembly.

Genome sequencing of the representative enediyne producers was performed using an Illumina MiSeq sequencer (2 × 300 paired-end sequencing) at the Next Generation Sequencing and Microarray Core Facility, TSRI. Read quality filtering was performed using a tool developed in-house. Adapter trimming and *de novo* assembly were done with CLC Genomics Workbench version 7.5.1 (CLC Bio.) using default settings. The resulting contigs were further extended and joined into a final scaffold by SSPACE version 2.0 (49) using all quality-filtered reads. The remaining gaps inside the final scaffold were partially or completely filled using the quality-filtered reads by GapFiller version 1.10 (50). The draft genome sequences of the selected hits (under BioProject PRJNA293172) and *S. uncialis* DCA2648 (under BioProject PRJNA286672) reported in this study have been deposited in GenBank, with their accession numbers summarized in Table S2.

### Enediyne genome neighborhood network (GNN).

Annotation of the new enediyne gene clusters and construction of the enediyne GNN were carried out as described previously (5, 6). Annotations of all the enediyne biosynthetic gene clusters reported in this study are summarized in Tables S3 and S4. The C-1027 gene cluster from *Streptomyces* sp. strain CB02366, the *ucm* gene cluster from *S. uncialis* DCA2648, and the *tnm* gene cluster from *Streptomyces* sp. strain CB03234 were deposited in GenBank under accession numbers KU597647, KT762610, and KT716443, respectively. Accession numbers for the other new enediyne gene clusters are listed in Table S2.

The enediyne GNN was constructed and included (i) the 7 known 9-membered enediyne gene clusters (encoding C-1027, NCS, MDP, KED, SPO, CYA, and CYN), (ii) the 3 known 10-membered enediyne gene clusters (encoding CAL, DYN, and ESP), (iii) the newly characterized gene cluster for the 10-membered enediyne UCM, and (iv) the 31 new enediyne gene clusters (i.e., the 28 distinct gene clusters plus the 3 homologous gene clusters from the same clade of CB00072) discovered in this study (Fig. 2). Cytoscape v 3.0 was used for GNN generation, visualization, and analysis (51). All GNNs were displayed using the “organic” layout with edge widths corresponding to the *E* value corresponding to comparisons between proteins. A more detailed description is provided in Text S1.

### Gene inactivation.

Inactivation of selected genes within the cloned enediyne clusters in *Streptomyces* species was performed by gene replacement following literature procedures (36, 37). The genotypes of the resultant mutants were confirmed by PCR (Table S1) and Southern analysis (Fig. S3) as described in Text S1.

### Fermentation, production, and HPLC analyses of C-1027.

The *Streptomyces* sp. strain CB02366 wild-type and SB1036 (i.e., Δ*pksE*) mutant strains were cultured individually following previously reported procedures (11), with the original C-1027 producer *S. globisporus* wild-type strain as a control. The identity of C-1027 was confirmed by HPLC and high-resolution electrospray ionization–mass spectrometry HR-ESI-MS analysis (Fig. 3B) as described in Text S1. To determine C-1027 titers, HPLC analysis was calibrated with an authentic C-1027 standard (9, 11).

### Fermentation, production, and HPLC analysis of UCM.

Fermentation of *S. uncialis* and production of UCM were performed on solid agar medium (ISP4) following previously published procedures (31). The identity of UCM was confirmed by HPLC and HR-ESI-MS analysis with an authentic UCM standard (Fig. 5C) as described in Text S1.

### Fermentation, production, and HPLC analyses of TNM A and C.

The *Streptomyces* sp. strain CB03234 wild-type, SB20001 (i.e., Δ*tnmE*), and SB20002 (i.e., Δ*tnmH*) mutant strains were cultured individually in 250-ml baffled flasks containing 50 ml of TSB liquid medium. After growth at 28°C and 250 rpm for 2 days, 5 ml of seed culture was inoculated into 250-ml baffled flasks containing 50 ml of the production medium (1% soluble starch, 0.5% Pharmamedia, 0.2% CaCO_3_, 0.005% CuSO_4⋅_ 5H_2_O, 0.0005% NaI, pH 7.0). The resulting cultures were incubated at 28°C and 250 rpm for 7 days and individually harvested. Each culture was centrifuged, the supernatant was extracted with EtOAc, and the cell pellet was extracted with CH_3_COCH_3_. The combined extracts were concentrated in vacuum and dissolved in CH_3_OH for HPLC and HR-ESI-MS analysis (Fig. 5D) as described in Text S1. TNM titers were determined by HPLC analysis calibrated with authentic standards.

### Isolation and structural elucidation of TNM A and C.

For structural elucidation, TNM A (1.2 mg) was isolated from 6 liters of fermentation culture of the *Streptomyces* sp. CB03234 wild-type strain, while TNM C (0.7 mg) was isolated from 6 liters of fermentation culture of the Δ*tnmH* mutant strain SB20002. The structures of TNM A and C were established on the basis of extensive MS, CD, and NMR analysis (Fig. 6 and Table S2). For further details, see Text S1.

### Cytotoxicity assay of TNMs.

The IC_50_s of TNMs against selected human cancer cell lines, including breast (MDA-MB-468, MDA-MB-231, SKBR-3, KPL-4, BT474, and DYT2), melanoma (M14 and SK-MEL-5), non-small cell lung cancer (NCI-H226), and central nervous system (SF-295 and SF-539) cells, with UCM as a control, were determined by following standard protocols described previously (47). Each point represents the mean ± standard deviation (SD) of results from three replicates, and the IC_50_ was determined by computerized curve fitting using GraphPad Prism (Table 1A). For comparison of the cell killing rates among TNM A, AFP, and AP-3, SK-BR-3 breast cancer cells were incubated with graded doses (ranging from 0 to 100 nM) of drugs for 8, 12, 24, and 72 h before the assays were developed as described above. Cell viability was expressed as a percentage of the level seen with untreated control cells (Tables 1B and C). Further details are provided in Text S1.

### Accession number(s).

Accession numbers for the C-1027 gene cluster from *Streptomyces* sp. strain CB02366, the *ucm* gene cluster from *S. uncialis* DCA2648, and the *tnm* gene cluster from *Streptomyces* sp. strain CB03234 are KU597647, KT762610, and KT716443, respectively. Accession numbers for the draft genomes of the selected 31 hits, as well as the UCM producer *S. uncialis* DCA2648, are summarized in Table S2.

## SUPPLEMENTAL MATERIAL

Figure S1  Known enediynes and selected natural products as ADC payloads. Download Figure S1, PDF file, 0.5 MB

Figure S2  A virtual survey of bacterial genomes for enediyne producers. Download Figure S2, PDF file, 0.5 MB

Figure S3  Construction and confirmation of SB1036, SB18001, SB20001, and SB20002. Download Figure S3, PDF file, 0.2 MB

Figure S4  ^1^H and ^13^C NMR spectra of TNM A and TNM C. Download Figure S4, PDF file, 0.3 MB

Table S1  Plasmids, strains, and primers used in this work. Table S1, PDF file, 0.1 MB

Table S2  Taxonomy, geography, and accession numbers of sequenced genomes. Table S2, PDF file, 0.4 MB

Table S3  Annotation of the C-1027, UCM, and TNM gene clusters from CB02366, *S. uncialis* DCA2648, and CB03234, respectively. Table S3, PDF file, 0.1 MB

Table S4  Annotation of the 29 enediyne gene clusters from the representative hits. Table S4, PDF file, 0.4 MB

Table S5  ^1^H and ^13^C NMR data and other physicochemical data of TNM A and C. Table S5, PDF file, 0.3 MB

Text S1  Supplemental Experimental Procedures. Download Text S1, PDF file, 0.3 MB
