# Supplementary material for: Strain Prioritization and Genome Mining for Enediyne Natural Products
Source: mBio. 2016 Dec 20;7(6):e02104-16. doi: 10.1128/mBio.02104-16 (PMC5181780; doi:10.1128/mBio.02104-16)

Figure S1. Related to Figure 2. Structures of the 11 enediyne natural products known to date and cytotoxic natural products as payloads for antibody-drug conjugates (ADCs). (A) The five 9-membered (NCS, C-1027, KED, MDP, N1999A2) and (B) six 10-membered (CAL, ESP, DYN, namenamicin, shishijimicin, UCM) enediyne natural products with the enediyne cores highlighted in red. SPO, CYA, CYN, and fijiolides were proposed to be derived from 9-membered enediyne precursors after cycloaromatization. The years when each of the enediyne structures was established are given in parentheses. (C) Structures of AFP and AP-3, analogues of the natural products auristatin and maytanisine used as payloads in the FDA-approved ADCs Adcetris® and Kadcyla®, respectively.

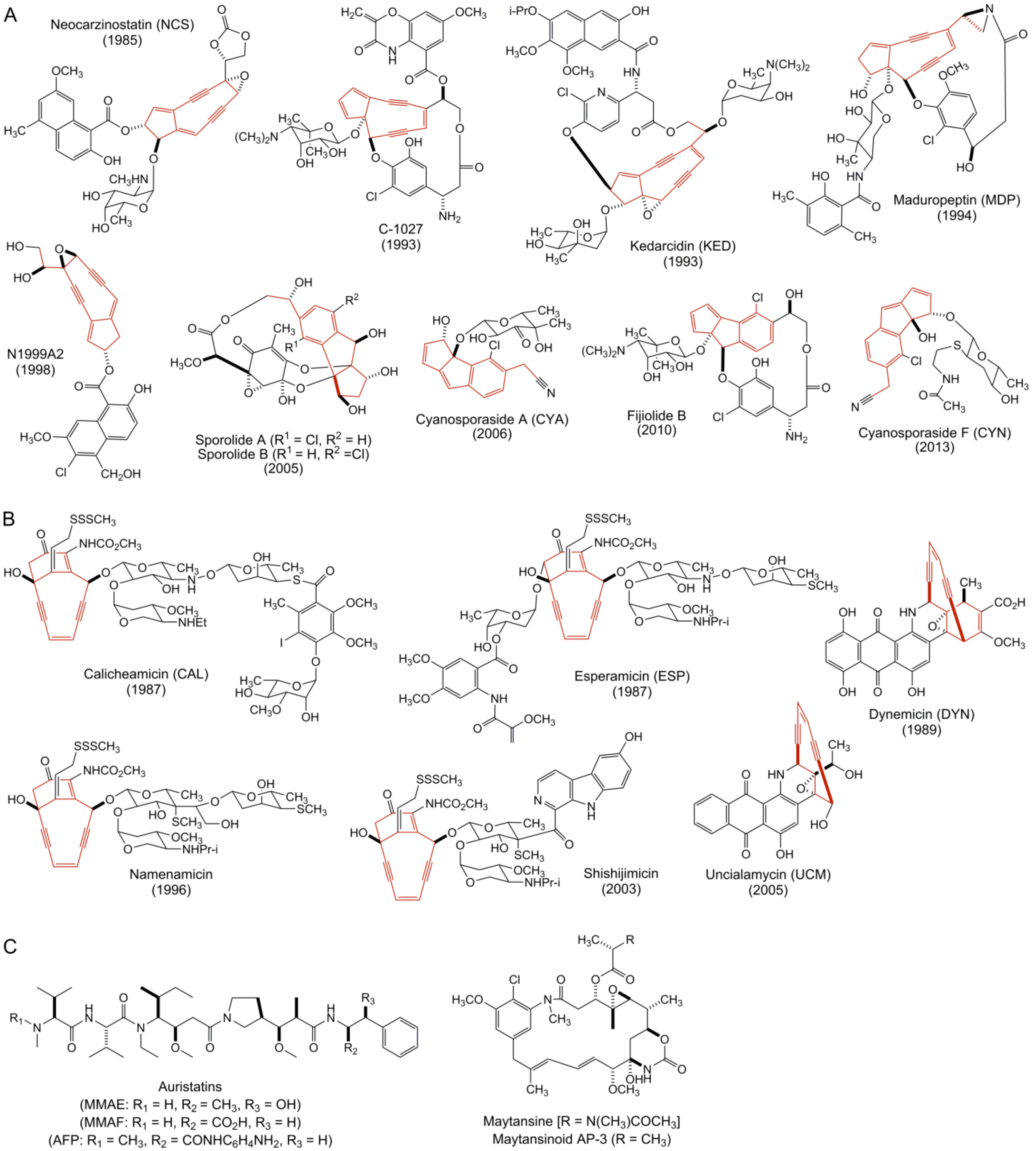

Supplement: Figure S1 — Known enediynes and selected natural products as ADC payloads. Download [file mbo006163128sf1.pdf]
