# Supplementary material for: Strain Prioritization and Genome Mining for Enediyne Natural Products
Source: mBio. 2016 Dec 20;7(6):e02104-16. doi: 10.1128/mBio.02104-16 (PMC5181780; doi:10.1128/mBio.02104-16)

Figure S3-1. Related to Figure 3. Confirmation of the cloned gene cluster from *Streptomyces* sp. CB02366 to encode C-1027 biosynthesis by inactivating the *pksE* gene to afford the Δ*pksE* mutant strain SB1036. (A) Construction of the Δ*pksE* mutant strain of SB1036 via a double crossover homologous recombination event between pBS1158 and the *Streptomyces* sp. CB02366 wild-type strain. Restriction maps showing an internal fragment of the *E* gene replaced by the apramycin resistance gene cassette *aac(3)IV* in the mutant strain SB1036. P, *Pst*I; Apr^R, apramycin resistant; Apr^S, apramycin sensitive; Tsr^R, thiostrepton resistant; Tsr^S, thiostrepton sensitive. (B) The genomic DNAs isolated from *Streptomyces* sp. CB02366 wild-type and SB1036 mutant strains and digested by *Pst*I and hybridized with a PCR amplified probe for Southern analysis, showing the expected sizes of 1.4-kb for the *Streptomyces* sp. CB02366 wild-type and 2.4-kb for the SB1036 mutant strains, respectively. Lane 1, *Streptomyces* sp. CB02366 wild-type; lane 2, SB1036 (Δ*pksE*) mutant; and lane 3, DNA ladder.

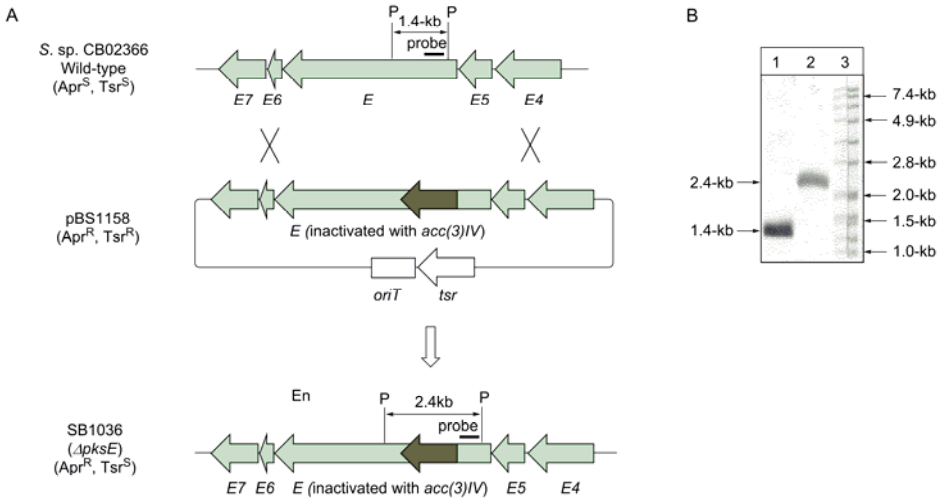

Figure S3-2. Related to Figure 5. Confirmation of the cloned gene cluster from *S. uncialis* to encode UCM biosynthesis by inactivating the *ucmE* gene to afford the Δ*ucmE* mutant strain of SB18001. (A) Construction of the Δ*ucmE* mutant strain of SB18001 via a double crossover homologous recombination event between pBS18004 and the *S. uncialis* wild-type strain. Restriction maps showing an internal fragment of the *ucmE* gene replaced by the apramycin resistance gene cassette *aac(3)IV* in the mutant strain SB18001. Ms, *Msc*I; Apr^R, apramycin resistant; Apr^S, apramycin sensitive; $Kana^R$, kanamycin resistant; $Kana^S$, kanamycin sensitive. (B) The genomic DNAs isolated from *S. uncialis* wild-type and SB18001 mutant strains and digested by *Msc*I and hybridized with a PCR amplified probe for Southern analysis, showing the expected sizes of 2.4-kb for the *S. uncialis* wild-type and 1.8-kb for the SB18001 mutant strains, respectively. Lane 1, *S. uncialis* wild-type; lane 2, SB18001 (Δ*ucmE*) mutant; and lane 3, DNA ladder.

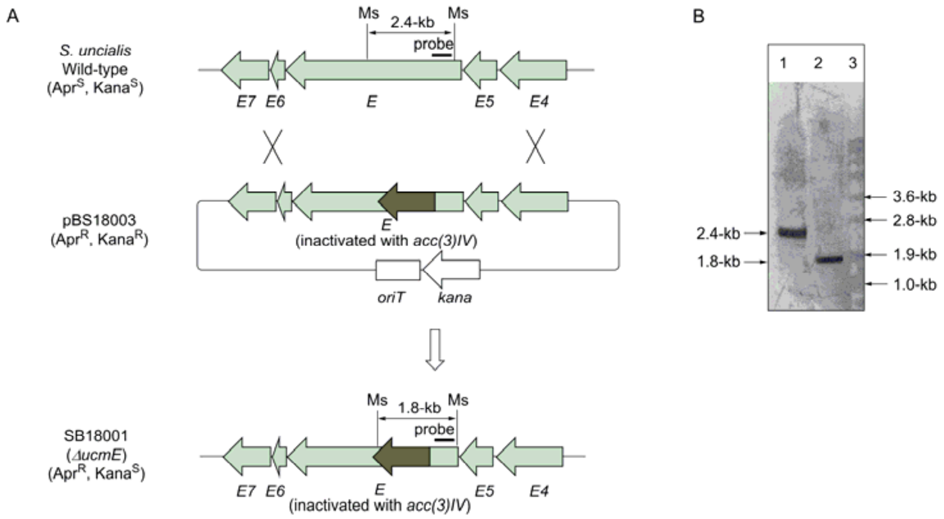

Figure S3-3. Related to Figure 5. Confirmation of the cloned gene cluster from *Streptomyces* sp. CB03234 to encode TNM biosynthesis by inactivating the *tnmE* gene to afford the Δ*tnmE* mutant strain of SB20001 and demonstration of manipulating TNM biosynthesis in *Streptomyces* sp. CB03234 by constructing the Δ*tnmH* mutant strain SB20002 to produce TNM C. (A) Construction of the Δ*tnmE* mutant strain of SB20001 via a double crossover homologous recombination event between pBS20004 and the *Streptomyces* sp. CB03234 wild-type strain. Restriction maps showing the upstream half of the *tnmE* gene replaced by the kanamycin resistance gene cassette *kana* in the mutant strain SB20001. (B) The genomic DNAs isolated from *Streptomyces* sp. CB03234 wild-type and SB20001 mutant strains and digested by *Eco*NI and hybridized with a PCR amplified probe for Southern analysis, showing the expected sizes of 3.9-kb for the *Streptomyces* sp. CB03234 wild-type and 8.8-kb for the SB20001 mutant strains, respectively. Lane 1, SB20001 (Δ*tnmE*) mutant; lane 2, *Streptomyces* sp. CB003234 wild-type; and lane 3, DNA ladder. (C) Construction of the Δ*tnmH* mutant strain of SB20002 via a double crossover homologous recombination event between pBS20005 and the *Streptomyces* sp. CB03234 wild-type strain. Restriction maps showing the *tnmH* gene replaced by the kanamycin resistance gene cassette *kana* in the mutant strain SB20002. (D) The genomic DNAs isolated from *Streptomyces* sp. CB03234 wild-type and SB20002 mutant strains and digested by *Eco*NI and hybridized with a PCR amplified probe for Southern analysis, showing the expected sizes of 6.6-kb for the *Streptomyces* sp. CB03234 wild-type and 9.1-kb for the SB20002 mutant strains, respectively. Lane 1, SB20002 (Δ*tnmH*) mutant; lane 2, *Streptomyces* sp. CB003234 wild-type; and lane 3, DNA ladder. En, *Eco*NI; Apr^R, apramycin resistant; Apr^S, apramycin sensitive; $Kana^R$, kanamycin resistant; $Kana^S$, kanamycin sensitive.

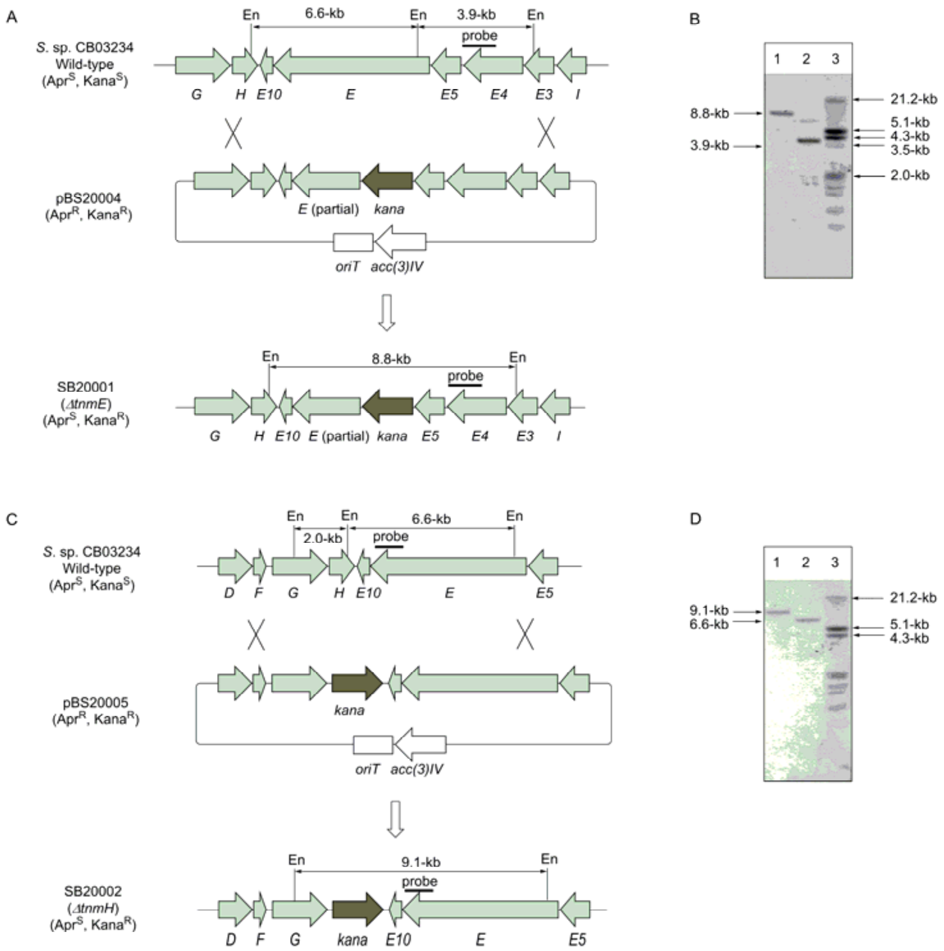

Supplement: Figure S3 — Construction and confirmation of SB1036, SB18001, SB20001, and SB20002. Download [file mbo006163128sf3.pdf]
