# Supplementary material for: Strain Prioritization and Genome Mining for Enediyne Natural Products
Source: mBio. 2016 Dec 20;7(6):e02104-16. doi: 10.1128/mBio.02104-16 (PMC5181780; doi:10.1128/mBio.02104-16)

Figure S4-1. Related to Figure 6. ^{1}H (700 MHz) and ^{13}C (175 MHz) NMR spectra of TNM A in acetone-d_6.

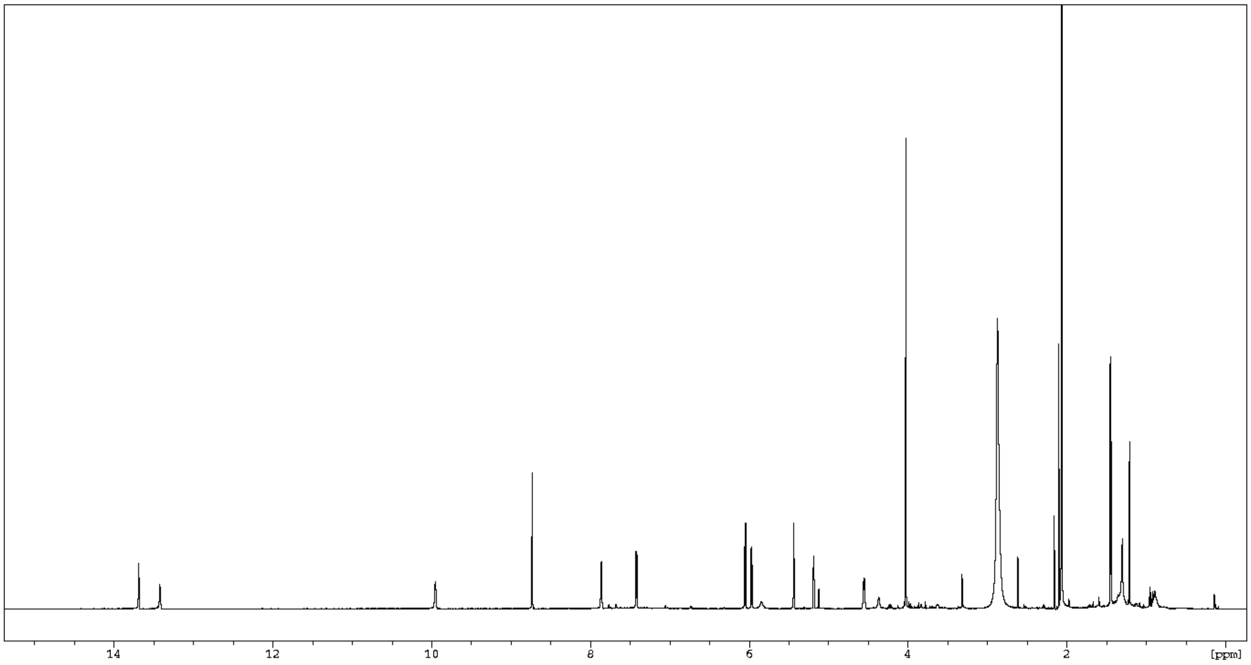

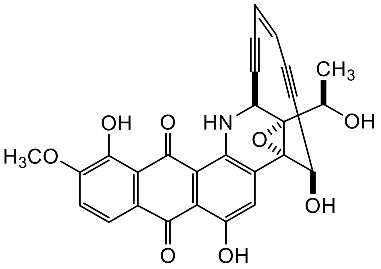

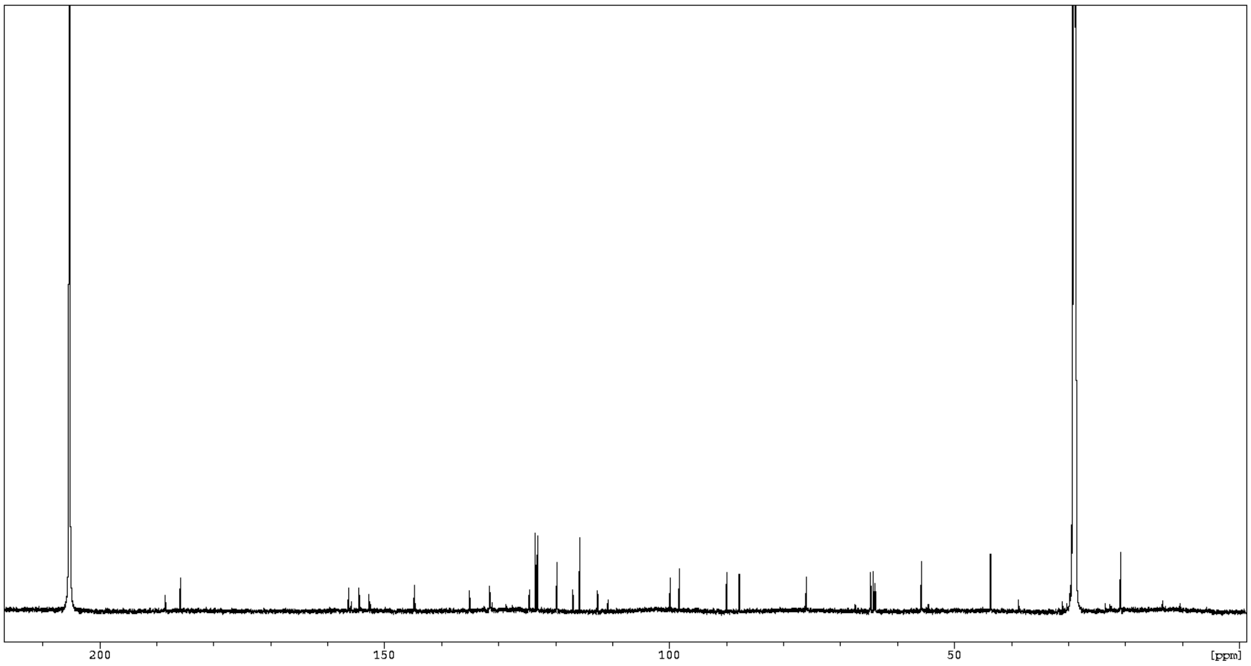

Figure S4-2. Related to Figure 6. HSQC and HMBC NMR spectra of TNM A in acetone-d_6.

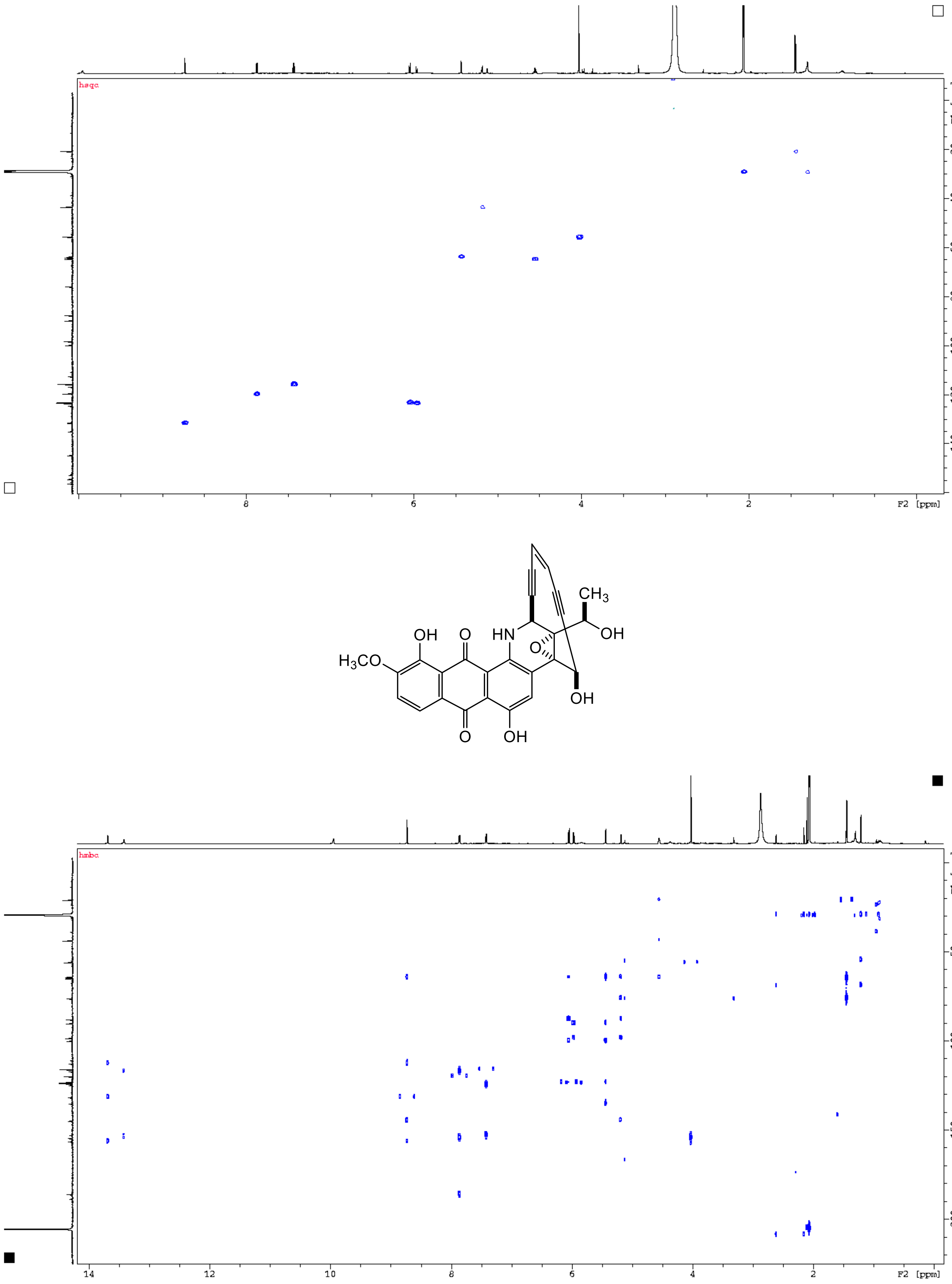

Figure S4-3. Related to Figure 6. ^{1}H-^{1}H COSY and ROESY NMR spectra of TNM A in acetone-d_6.

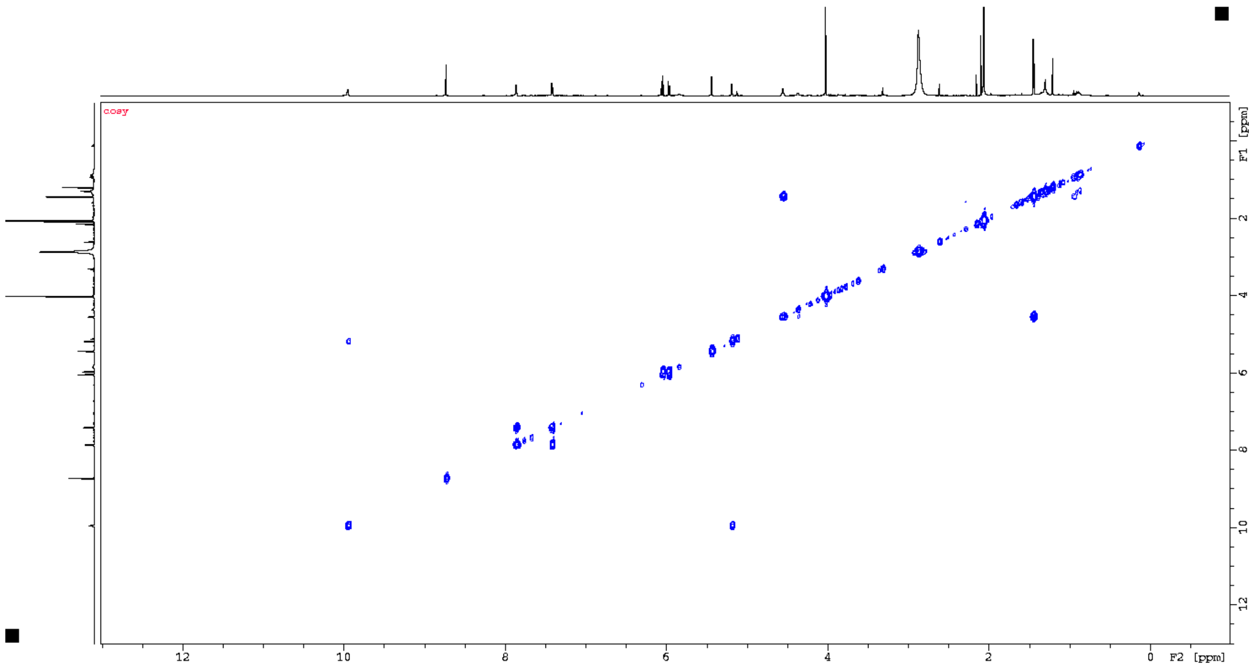

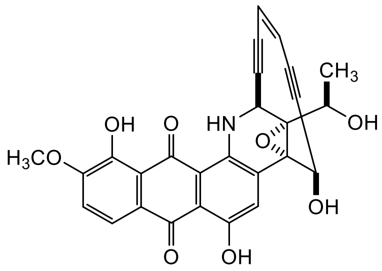

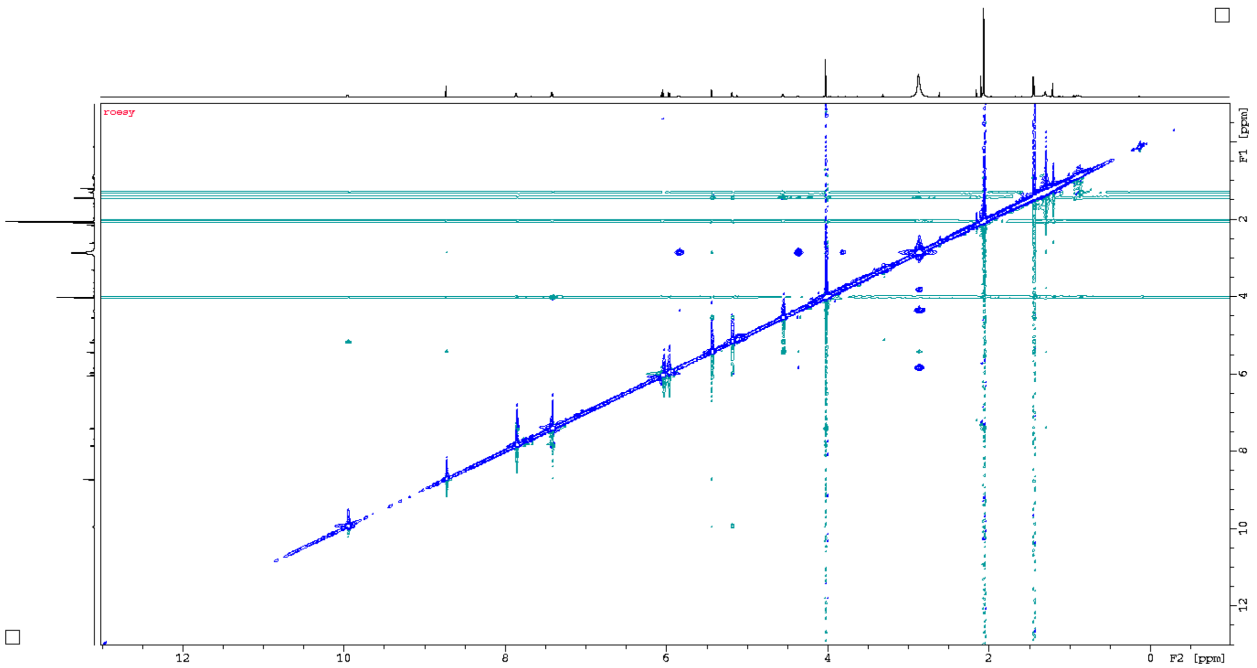

Figure S4-4. Related to Figure 6. ^{1}H (700 MHz) and ^{13}C (175 MHz) NMR spectra of TNM C in acetone-d_6.

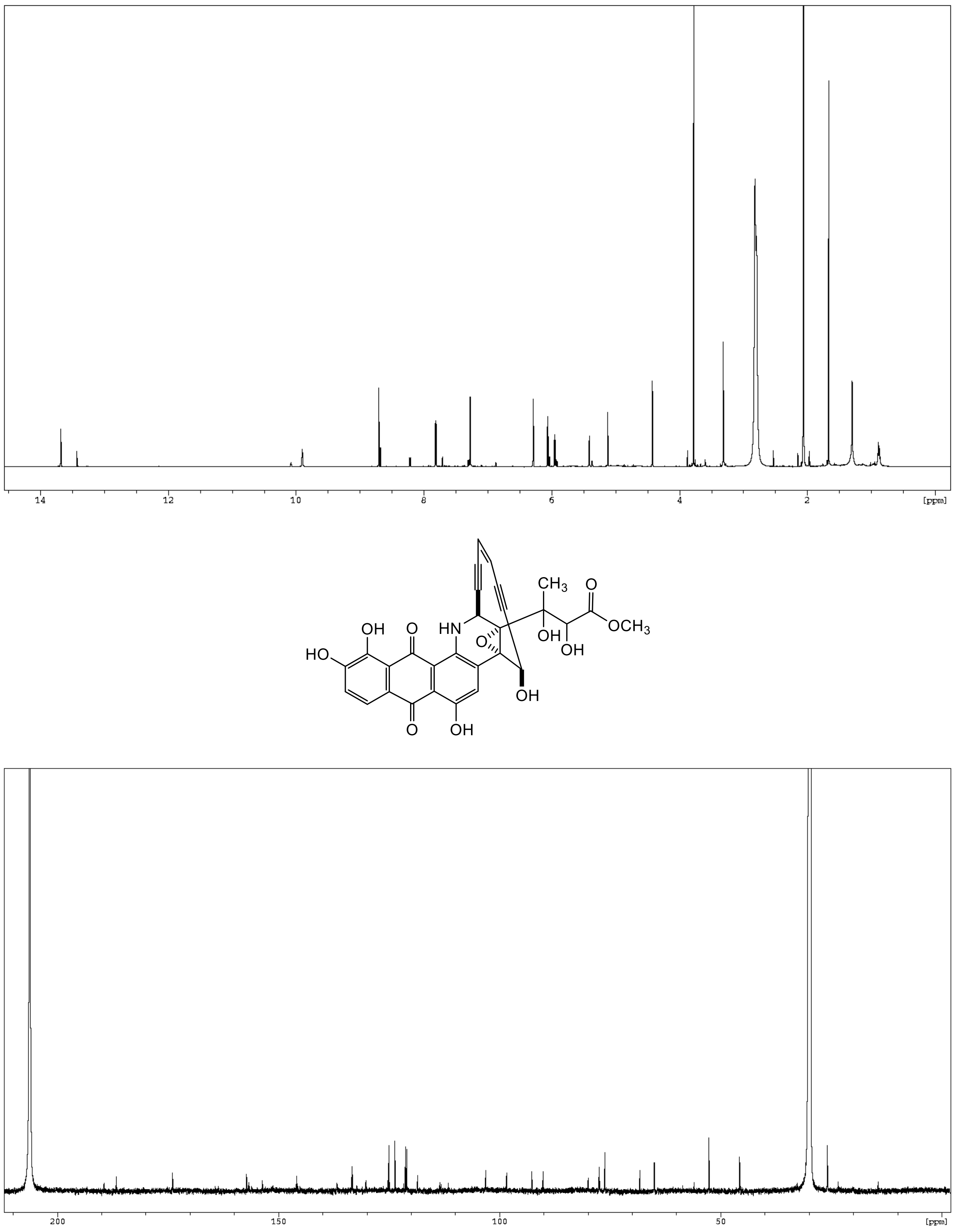

Figure S4-5. Related to Figure 6. HSQC and HMBC NMR spectra of TNM C in acetone-d_6.

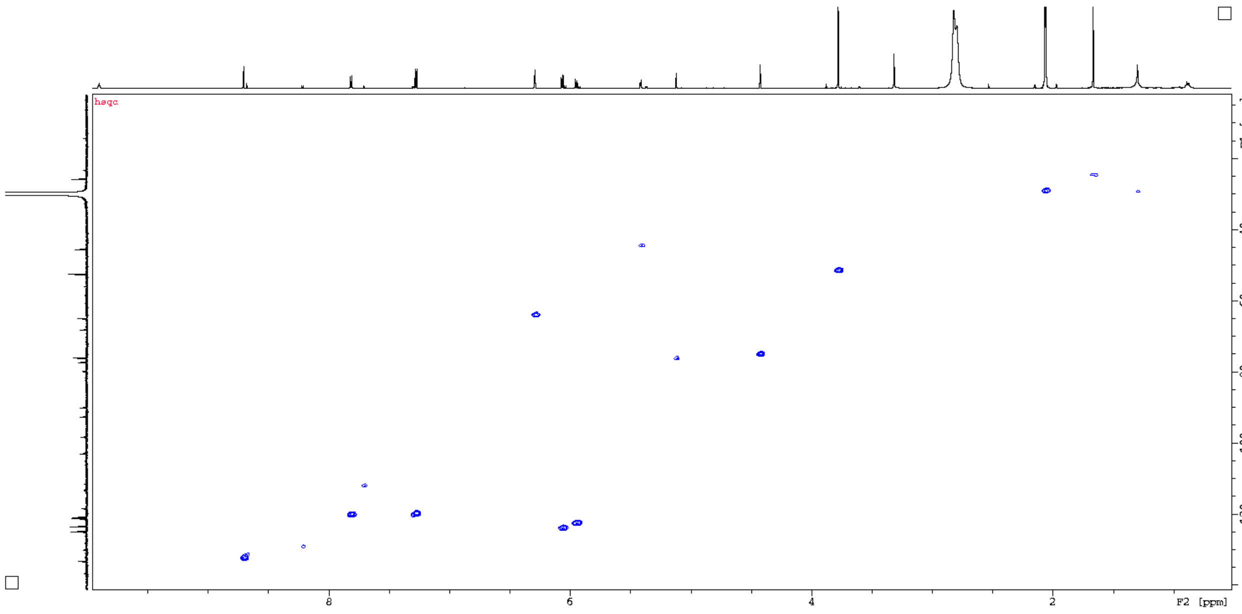

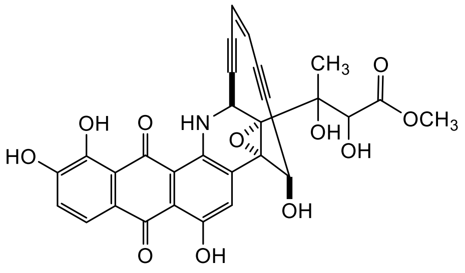

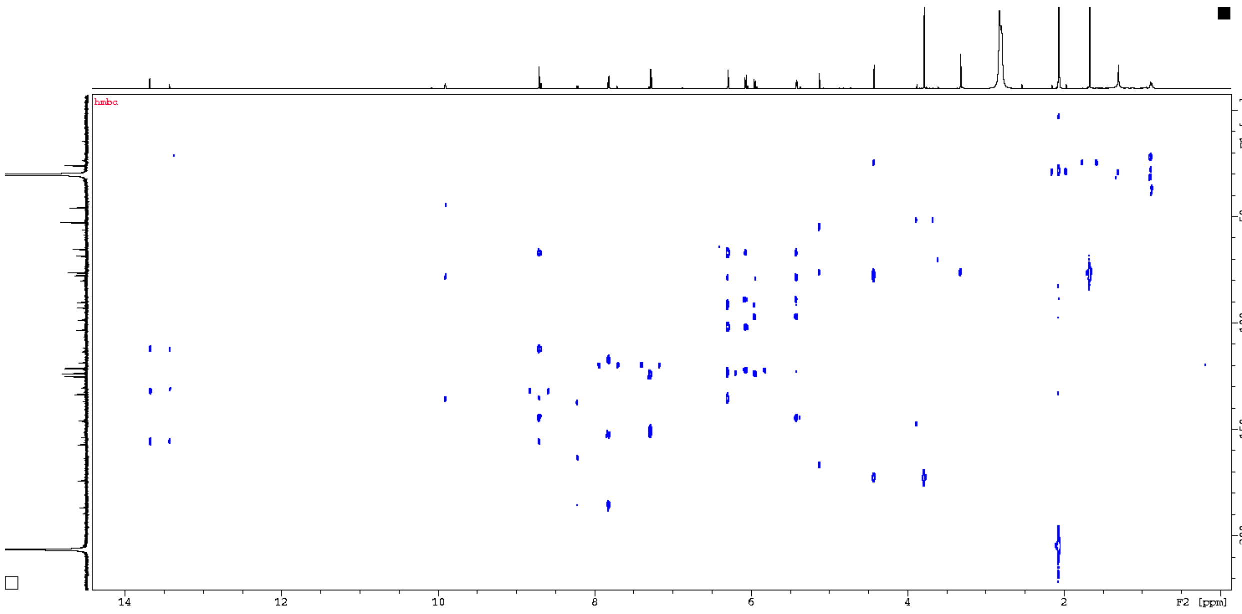

Figure S4-6. Related to Figure 6. ^{1}H-^{1}H COSY and ROESY NMR spectra of TNM C in acetone-d_6.

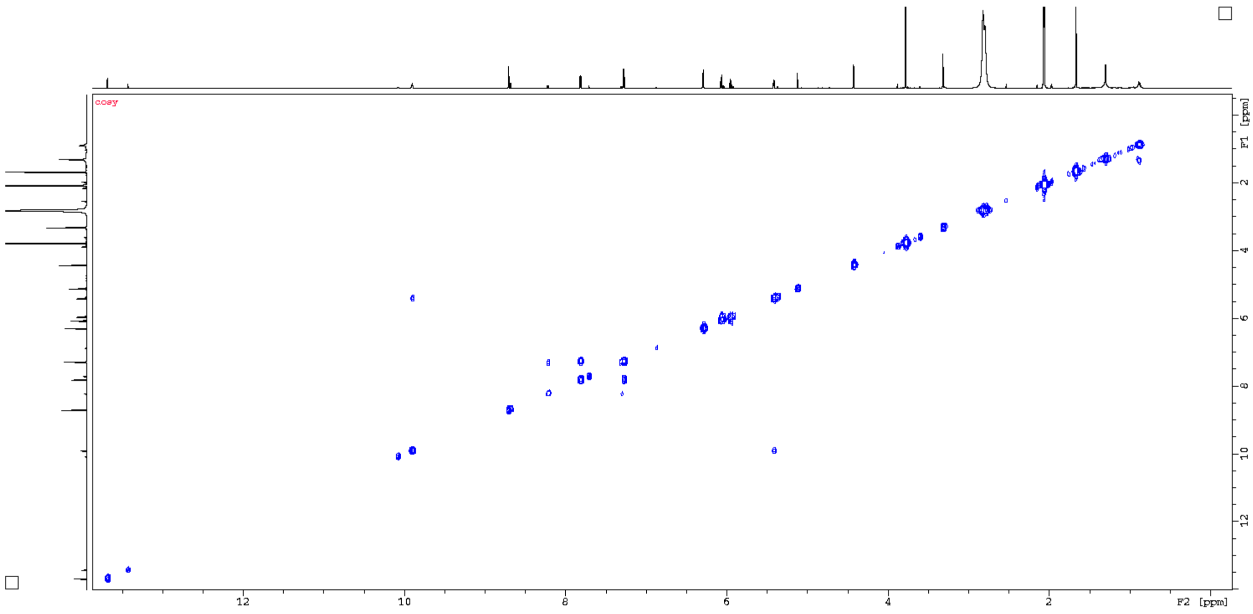

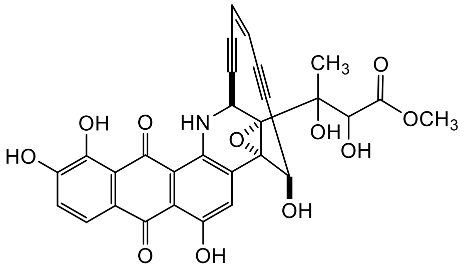

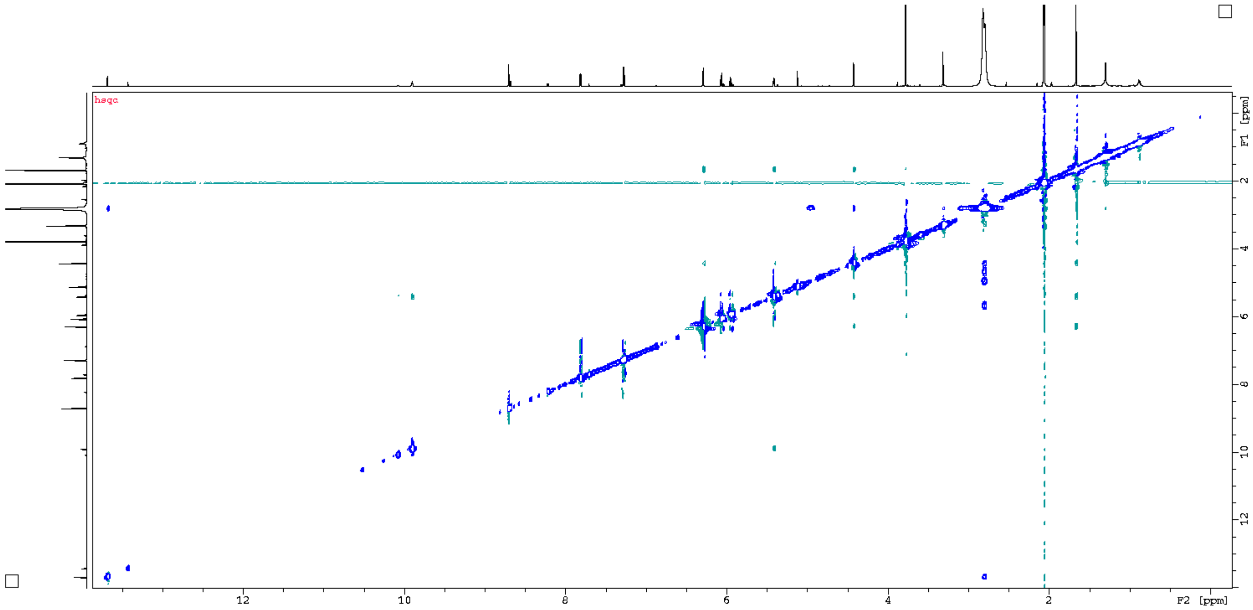

Supplement: Figure S4 — 1H and 13C NMR spectra of TNM A and TNM C. Download [file mbo006163128sf4.pdf]
