# Supplementary material for: Strain Prioritization and Genome Mining for Enediyne Natural Products
Source: mBio. 2016 Dec 20;7(6):e02104-16. doi: 10.1128/mBio.02104-16 (PMC5181780; doi:10.1128/mBio.02104-16)
Supplement: Table S1 — Plasmids, strains, and primers used in this work. [file mbo006163128st1.pdf]

Table S1-1. Related to Experimental Procedures. Plasmids and strains used in this study

strain/plasmid	genotype and description	reference
E. coli strains		
DH5α	*E. coli* host for cloning	2
XL1-Blue MRF	*E. coli* host for cosmid library construction	2
ET12567/pUZ8002	Methylation-deficient *E. coli* host for intergeneric conjugation; contains pUZ8002, a nontransmissible *oriT* mobilizing plasmid.	3
BW25113/pIJ790	*E. coli* host for PCR targeting	4
Streptomyces strains		
S. globisporus C-1027	The original C-1027 producer discovered in 1993, wild-type	5
Streptomyces sp. CB02366	The newly discovered C-1027 producer, wild-type	This study
SB1036	The Δ*sgcE* mutant strain of CB02366	This study
Streptomyces sp. CB03234	The newly discovered TNM producer, wild-type	This study
SB20001	The Δ*tnmE* mutant strain of CB03234	This study
SB20002	The Δ*tnmH* mutant strain of CB03234	This study
SB20003	The CB03234 recombinant strain overexpressing *tnmR3* under *ErmE**	This study
SB20004	The CB03234 recombinant strain overexpressing *tnmR4* under *ErmE**	This study
SB20005	The CB03234 recombinant strain overexpressing *tnmR7* under *ErmE**	This study
S. uncialis DCA2648	The original UCM producer discovered in 2005, wild-type	6
SB18001	The Δ*ucmE* mutant strain of *S. uncialis* DCA2648	This study
Plasmids		
pJTU4659	Plasmid contains kanamycin cassette for PCR targeting template	This study
pIJ773	Plasmid containing the apramycin resistance cassette (*aac(3)IV* + *oriT*)	3
SuperCos1	Vector for the construction of cosmid library	2
SuperCos1.1	SuperCos1 variant with the kanamycin resistance gene replaced by *aac(3)IV* + *oriT*	This study
pHJL401T	*Streptomyces*-*E. coli* shuttle vector	7
pBS1156	The 2.6-kb upstream fragment of *pksE* from the enediyne cluster in CB02366 cloned into the *Hin*dIII/*Xba*I sites of pHJL401T	This study
pBS1157	The 2.7-kb downstream fragment of *pksE* from the enediyne cluster in CB02366 cloned into the *Xba*I/*Eco*RI sites of pBS1156	This study
pBS1158	A 1.3-kb fragment containing the *aac(3)IV* + *oriT* cassette inserted into the *Xba*I site of pBS1157	This study
pBS20001	Cosmid 4G2 from CB03234 cosmid library, containing partial *tnm* gene cluster	This study
pBS20002	Cosmid 11H1 from CB03234 cosmid library, containing partial *tnm* gene cluster	This study
pBS20003	Cosmid 4C3 from CB03234 cosmid library, containing partial *tnm* gene cluster	This study
pBS20004	pBS20002 with *tnmE* inactivated with kanamycin resistance cassette by PCR targeting (i.e., Δ*tnmE*)	This study
pBS20005	pBS20002 with *tnmH* inactivated with kanamycin resistance cassette by PCR targeting (i.e., Δ*tnmH*)	This study
pBS18002	Cosmid 13B3 from *S. uncialis* cosmid library, containing partial *ucm* gene cluster	This study
pBS18003	Cosmid 20A3 from *S. uncialis* cosmid library, containing partial *ucm* gene cluster	This study
pBS18004	pBS18003 with *ucmE* inactivated with *aac(3)IV* + *oriT* by PCR targeting (i.e., Δ*ucmE*)	This study

Table S1-2. Related to Experimental Procedures. Primers used in this study

primer	nucleotide sequence (5'-3')	function
E5T3KS-S	CCCCGCVCACATCACSGSCCTCGCSGTGAACATGCT	PCR targeting the *E5*/*E* regions
E5T3KS-AS	GCAGGCKCCGTCSACSGTGTABCCGCCGCC	PCR targeting the *E5*/*E* regions
CTE10E7-S	GCVGTGVTSGSCMTGGAGGCGATG	PCR targeting the *E*/*E10* regions
CTE10E7-AS	TCCCGGCASYGSCCCTGCC	PCR targeting the *E*/*E10* regions
EKSAT-S	TGTAAAACGACGGCCAGTATGGGSTTCGGCGGSATCAAC	Confirmation of the KS-AT sequence
EKSAT-AS	CAGGAAACAGCTATGACCAGMGGNGAGTGGAANGCGTG	Confirmation of the KS-AT sequence
16SrRNA_for	AGAGTTTGATCCTGGCTCAG	Phylogenetic analysis
16SrRNA_rev	ACGGCTACCTTGTTACGACTT	Phylogenetic analysis
rpoB-2	CATCGACCACTTCGGCAAC	Phylogenetic analysis
ActRpoB3303R	GAANCGCTGDCCRCCGAACTG	Phylogenetic analysis
trpBfor	TAATACGACTCACTATAGGGGCGCGAGGACCTGAACCACAC	Phylogenetic analysis
trpBrev	GCTAGTTATTGCTCAGCGGCATGGCCGGGATGATGCCC	Phylogenetic analysis
2366pksEUpF	CGGAAGCTTGGTCTCGCCGTGAACATGCT (the *Hin*dIII site is underlined)	Amplifying the upstream homologous arm of *pksE* in *Streptomyces* sp. CB02366
2366pksEUpR	GCTCTAGAACGTGCCGGAACCCTGCCCC (the *Xba*I site is underlined)	Amplifying the upstream homologous arm of *pksE* in *Streptomyces* sp. CB02366
2366pksEDnF	GCTCTAGAGAGGCCGACATCGCGCTCGG (the *Xba*I site is underlined)	Amplifying the downstream homologous arm of *pksE* in *Streptomyces* sp. CB02366
2366pksEDnR	CGGAATTCGAAGTGCTCCGCCGCGAAAC (the *Eco*RI site is underlined)	Amplifying the downstream homologous arm of *pksE* in *Streptomyces* sp. CB02366
KD-2366pksEF	TGGACCCCCACGACCTGCTC	SB1036 mutant PCR confirmation
KD-2366pksER	TGACGATCTGACCGACCGTG	SB1036 mutant PCR confirmation
SH-2366pksEF	CTCCTCCGACGGTCAGGGAG	Southern blotting confirmation of SB1036 mutant
SH-2366pksER	TCCCCGTCCAGGAGCAGCAG	Southern blotting confirmation of SB1036 mutant
tnmE-RedETF	GTGACGTCCACGATCGCCGTCGTCGGCATGGCGTGCCGGATT CCGGGGATCCGTCGACC	λRED-mediated PCR targeting replacement of *tnmE*
tnmE-RedETR	ATCGGCGAGGAGCAGGTCGTCGGCGCGGATCGACTCGGGTGT AGGCTGGAGCTGCTTC	λRED-mediated PCR targeting replacement of *tnmE*
KD-tnmEdelF	GACTCGGCACGAGGCGGTGCGT	SB20001 PCR confirmation
KD-tnmEdelF	CGTCAGTTCGCGTTCGGCCTGGGC	SB20001 PCR confirmation
SH-tnmEdelF	GAACGTCCGGGTGATCCGGT	Southern blotting confirmation of SB20001 mutant
SH-tnmEdelR	CGTCATGACACCCAAGTCGAAGGTG	Southern blotting confirmation of SB20001 mutant
tnmH-RedETF	ATGGCCGACGACGACGCGTTCGTCCACCTGCTGCGGCTCTGT AGGCTGGAGCTGCTTC	λRED-mediated PCR targeting replacement of *tnmH*
tnmH-RedETR	TCAGCCGGCCGCCGACGTCTCCGCCTCGCACACCACCAGATT CCGGGGATCCGTCGACC	λRED-mediated PCR targeting replacement of *tnmH*
KD-tnmHdelF	GTACGCCACGAGCACCCTGT	SB20002 PCR confirmation
KD-tnmHdelR	GCCACCAGACCATCGCCTGC	SB20002 PCR confirmation
SH-tnmHdelF	GTACGCGAAGTCCATGGTGAC	Southern blotting confirmation of SB20002 mutant
SH-tnmHdelR	CCGAGAAGGGCTTCGTCTC	Southern blotting confirmation of SB20002 mutant
ucmE-RedETF	CTACACACTGGCCGGGTACGCGCCCGGGTCCGTCGGCTG ATTCCGGGGATCCGTCGACC	λRED-mediated PCR targeting replacement of *ucmE*

ucmE-RedETR	GTCGCCGTGGCCATCGGGGTCATCTCCACCGGCGGATCCTGT AGGCTGGAGCTGCTTC	λRED-mediated PCR targeting replacement of *ucmE*
KD-ucmEdelF	GCAGACCGTCGTCCTTGACCG	SB18001 PCR confirmation
KD-ucmEdelR	CCACGTTCCGCACCACCGACAC	SB18001 PCR confirmation
SH-ucmEdelF	ACCGTGGCCGGACGGATC	Southern blotting confirmation of SB18001 mutant
SH-ucmEdelR	AGTGTGTAGGCCCGGTGG	Southern blotting confirmation of SB18001 mutant
