# Supplementary material for: Strain Prioritization and Genome Mining for Enediyne Natural Products
Source: mBio. 2016 Dec 20;7(6):e02104-16. doi: 10.1128/mBio.02104-16 (PMC5181780; doi:10.1128/mBio.02104-16)
Supplement: Table S2 — Taxonomy, geography, and accession numbers of sequenced genomes. [file mbo006163128st2.pdf]

Table S2. Related to Figures 1, 2, 4. Taxonomy, geography, and genome sequences of the 81 hits and accession numbers associated with the *pksE* genes (partial), the selected housekeeping genes for taxonomic identification, and the draft genomes for the selected 31 hits, as well as the UCM producer *S. uncialis* DCA2648

			NCBI accession number				
strain[a]	location[b]	taxonomy[c]	*pksE*[d]	*16S rRNA*	*rpoB*	*trpB*	genome
CB01143	China	*Streptomyces* sp.	KT736388	KT722926	KT736486	KT793907	
CB01083	China	*Streptomyces* sp.	KT736394	KT722912	KT736484	KT793905	
CB01603	China	*Streptomyces* sp.	KT736346	KT722853	KT736415	KT793857	
CB02341	China	*Streptomyces* sp.	KT736321	KT722890	KT736446	KT793879	
**CB02009**●	China	*Streptomyces* sp.	KT736363	KT722855	KT736429	KT793842	LIVT00000000
CB02459	China	*Streptomyces* sp.	KT736329	KT722886	KT736442	KT793861	
**CB02261**●	China	*Streptomyces* sp.	KT736371	KT722859	KT736440	KT793838	LIVX00000000
CB02736	Malaysia	*Streptomyces* sp.	KT736372	KT722929	KT736473	KT793891	
**CB02130**●	China	*Streptomyces* sp.	KT736349	KT722858	KT736437	KT793839	LIXK00000000
TSRI0246	TSRI	*Streptomyces* sp.	KT736370	KT722898	KT736472	KT793916	
**CB00455**●	China	*Streptomyces* sp.	KT736382	KT722906	KT736464	KT793889	LIVQ00000000
TSRI0050	TSRI	*Streptomyces* sp.	KT736345	KT722867	KT736418	KT793850	
TSRI0272	TSRI	*Streptomyces* sp.	KT736339	KT722866	KT736420	KT793858	
**CB03578**●	China	*Streptomyces* sp.	KT736395	KT722909	KT736482	KT793902	LWLD00000000
**CB01883**●	China	*Streptomyces* sp.	KT736344	KT722854	KT736417	KT793843	LIWA00000000
CB02510	UAE	*Streptomyces* sp.	KT736376	KT722899	KT736457	KT793893	
**CB02400**●	UAE	*Streptomyces* sp.	KT736324	KT722865	KT736412	KT793876	LIWB00000000
CB02507	UAE	*Streptomyces* sp.	KT736326	KT722885	KT736441	KT793862	
TSRI0262	TSRI	*Streptomyces* sp.	KT736386	KT722914	KT736485	KT793906	
TSRI0275	TSRI	*Streptomyces* sp.	KT736338	KT722869	KT736454	KT793886	
**TSRI0281**●	TSRI	*Streptomyces* sp.	KT736336	KT722871	KT736423	KT793847	LWLE00000000
TSRI0279	TSRI	*Streptomyces* sp.	KT736337	KT722870	KT736422	KT793848	
CB01199	China	*Streptomyces* sp.	KT736374	KT722902	KT736477	KT793896	
**CB01580**●	China	*Streptomyces* sp.	KT736348	KT722849	KT736460	KT793851	LIXJ00000000
CB01388	China	*Streptomyces* sp.	KT736354	KT722881	KT736438	KT793853	
**CB00316**●	Germany	*Streptomyces* sp.	KT736381	KT722907	KT736465	KT793890	LIXI00000000
CB00657	China	*Streptomyces* sp.	KT736389	KT722843	KT736406	KT793915	
CB03608	China	*Streptomyces* sp.	KT736385	KT722844	KT736407	KT793901	
CB02329	China	*Streptomyces* sp.	KT736327	KT722845	KT736408	KT793863	
**CB02366**●	UAE	*Streptomyces griseus*	KT736331	KT722846	KT736409	KT793837	LIVT00000000
**CB02058**●	China	*Streptomyces* sp.	KT736383	KT722905	KT736462	KT793888	LIPG00000000
CB01894	China	*Streptomyces* sp.	KT736343	KT722875	KT736455	KT793913	
**CB02414**●	UAE	*Streptomyces* sp.	KT736330	KT722889	KT736463	KT793864	LIPF00000000
**CB02460**●	China	*Streptomyces* sp.	KT736320	KT722860	KT736445	KT793836	LIVY00000000
CB02030	China	*Streptomyces* sp.	KT736341	KT722876	KT736428	KT793841	
CB01764	China	*Streptomyces* sp.	KT736350	KT722884	KT736439	KT793852	
CB02467	China	*Streptomyces* sp.	KT736357	KT722888	KT736444	KT793865	
CB00305	Germany	*Streptomyces* sp.	KT736387	KT722916	KT736460	KT793908	
CB00324	Germany	*Streptomyces* sp.	KT736380	KT722903	KT736479	KT793898	
CB01300	China	*Streptomyces* sp.	KT736368	KT722932	KT736470	KT793911	
CB00225	China	*Streptomyces* sp.	KT736399	KT722930	KT736469	KT793910	
CB00229	China	*Streptomyces* sp.	KT736400	KT722931	KT736471	KT793912	
**TSRI0395**[e,◆]	TSRI	*Streptomyces* sp.	KT736358	KT722862	KT736436	KT793856	LIPE00000000
**TSRI0261**[e,◆]	TSRI	*Streptomyces* sp.	KT736340	KT722850	KT736413	KT793884	LISY00000000
TSRI0149	TSRI	*Streptomyces* sp.	KT736333	KT722873	KT736426	KT793844	

TSRI0367	TSRI	*Streptomyces* sp.	KT736359	KT722878	KT736432	KT793869	
CB02802	China	*Streptomyces* sp.	KT736375	KT722927	KT736474	KT793892	
TSRI0287	TSRI	*Streptomyces* sp.	KT736335	KT722872	KT736424	KT793846	
CB02477	China	*Streptomyces* sp.	KT736323	KT722891	KT736447	KT793877	
CB03629	China	*Streptomyces* sp.	KT736384	KT722908	KT736481	KT793900	
CB00326	Germany	*Streptomyces* sp.	KT736397	KT722904	KT736480	KT793899	
**CB02115**[e,◆]	China	*Streptomyces* sp.	KT736332	KT722857	KT736430	KT793840	LIVW00000000
**CB00072**[e,◆]	China	*Streptomyces* sp.	KT736342	KT722863	KT736427	KT793855	LIPB00000000
TSRI0148	TSRI	*Streptomyces* sp.	KT736334	KT722874	KT736425	KT793845	
CB01106	China	*Streptomyces* sp.	KT736328	KT722851	KT736410	KT793860	
CB02913	China	*Streptomyces* sp.	KT736379	KT722928	KT736478	KT793897	
CB01059	China	*Streptomyces* sp.	KT736362	KT722882	KT736456	KT793887	
**TSRI0445**[◆]	TSRI	*Streptomyces* sp.	KT736360	KT722879	KT736433	KT793868	LIPC00000000
CB01064	China	*Streptomyces sp.*	KT736361	KT722877	KT736431	KT793870	
**CB01531**[●]	China	*Micromonospora sp.*	KT736352	KT722883	KT736468	KT793914	LWLS00000000
TSRI0375	TSRI	*Micromonospora sp.*	KT736392	KT722913	KT736458	KT793882	
**TSRI0369**[◆]	TSRI	*Micromonospora sp.*	KT736355	KT722861	KT736435	KT793880	LIVU00000000
TSRI0388	TSRI	*Micromonospora sp.*	KT736393	KT722915	KT736459	KT793883	
TSRI0356	TSRI	*Micromonospora sp.*	KT736356	KT722880	KT736434	KT793881	
CB01132	China	*Streptomyces sp.*	KT736353	KT722887	KT736443	KT793866	
CB02865	China	*Streptomyces sp.*	KT736373	KT722901	KT736476	KT793895	
**CB01249**[●]	China	*Streptomyces sp.*	KT736378	KT722852	KT736414	KT793854	LISW00000000
CB02520	UAE	*Streptomyces sp.*	KT736325	KT722892	KT736448	KT793875	
**CB03911**[◆]	China	*Streptomyces sp.*	KT736401	KU160265	KT736487	KT793909	LWLA00000000
**TSRI0107**[●]	TSRI	*Streptomyces sp.*	KT736369	KT722897	KT736421	KT793885	LIVZ00000000
**CB02923**[●]	China	*Streptomyces sp.*	KT736391	KT722911	KT736483	KT793904	LWKZ00000000
CB02853	China	*Streptomyces sp.*	KT736377	KT722900	KT736475	KT793894	
CB03299	China	*Streptomyces sp.*	KT736390	KT722910	KT736466	KT793903	
**CB02488**[◆]	China	*Streptomyces sp.*	KT736322	KT722848	KT736411	KT793878	LIVO00000000
**CB01950**[◆]	China	*Kitasatospora sp.*	KT736347	KT722864	KT736416	KT793859	LISX00000000
CB01783	China	*Amycolatopsis* sp.	KT736398	KT722893	KT736449	KT793874	
**CB00013**[●]	China	*Amycolatopsis* sp.	KT736366	KT722894	KT736453	KT793867	LIWC00000000
CB02065	China	*Streptomyces* sp.	KT736364	KT722895	KT736451	KT793872	
**CB02056**[●]	China	*Kitasatospora* sp.	KT736365	KT722856	KT736450	KT793873	LIPD00000000
**TSRI0078**[●]	TSRI	*Nocardiopsis* sp.	KT736351	KT722868	KT736419	KT793849	LWLB00000000
**CB03234**[◆]	China	*Streptomyces venezuelae*	KT736396	KT722842	KT736402	KT736405	LIYH00000000
**DCA2648**[◆]	Canada	*Streptomyces uncialis*	KJ652675	JN177509	-	-	LFBV00000000

[a]Representative strains from the 28 clades whose genomes have been sequenced (also see Figure 2). Strains predicted to contain 9-membered (●) or 10-membered (◆) enediyne biosynthetic gene clusters.
[b]Geographic distribution of the 81 hits, UAE abbreviated for United Arab Emirates, and TSRI denoted legacy strains in the TSRI strain collection whose geographic origins are not available (also see Figure 1D).
[c]Taxonomy classification based on the three housekeeping genes of *16S rRNA, rpoB, trpB* (also see Figure 1E).
[d]The internal PKSE fragment (Figure 1B) used to construct the phylogenetic tree for clade determination (Figure 2A).
[e]Selected strains from the same clade (Figure 2, panel B) that have been sequenced, yielding highly homologous gene clusters (also see Table S4), and only the cluster from CB00072 was shown in Figure 2C.
