# Supplementary material for: Strain Prioritization and Genome Mining for Enediyne Natural Products
Source: mBio. 2016 Dec 20;7(6):e02104-16. doi: 10.1128/mBio.02104-16 (PMC5181780; doi:10.1128/mBio.02104-16)
Supplement: Table S3 — Annotation of the C-1027, UCM, and TNM gene clusters from CB02366, S. uncialis DCA2648, and CB03234, respectively. [file mbo006163128st3.pdf]

Table S3-1. Related to Figures 2, 3, 4. Predicted functions of ORFs in the C-1027 biosynthetic gene cluster from *Streptomyces* sp. CB02366 in comparison with the C-1027 gene cluster from *S. globisporus* (5)

gene[a]	aa[b]	putative function[c]	protein homologue	% identity/ % similarity
CB02366_*orf(-1)*	491	DNA primase	BF14_2521 (KIX45972)	93/96
CB02366_sgcB1	456	Glycerol phosphate ABC transporter	SgcB1 (AAL06653)	91/95
CB02366_sgcB2	775	UvrA-like drug resistance pump	SgcB2 (AAL06654)	98/98
CB02366_sgcB3	461	Na/H efflux pump	SgcB3 (AAL06655)	91/94
CB02366_sgcC3	494	Alkylhalidase	SgcC3 (AAL06656)	95/97
CB02366_sgcA1	355	dNTP-glucose synthase	SgcA1 (AAL06657)	91/95
CB02366_cagA	143	Apoprotein	CagA (AAL06658)	94/97
CB02366_sgcA4	410	Amino transferase	SgcA4 (AAL06659)	93/96
CB02366_sgcA5	244	*N*-methyl transferase	SgcA5 (AAL06660)	98/97
CB02366_sgcA3	423	*C*-methyltransferase	SgcA3 (AAL06661)	99/99
CB02366_sgcF	385	Epoxide hydrolase	SgcF (AAL06662)	97/98
CB02366_sgcD1	220	Anthranilate synthase II	SgcD1 (AAL06663)	95/97
CB02366_sgcD	493	2-Amino-4-deoxychorismate synthase	SgcD (AAL06664)	95/96
CB02366_sgcD5	484	Phenylacetyl-CoA ligase	SgcD5 (AAL06665)	97/97
CB02366_sgcG	223	2-Amino-4-deoxychorismate dehydrogenase	SgcG (AAL06666)	97/98
CB02366_sgcD6	440	3-*O*-Acyltransferase	SgcD6 (AAL06667)	97/98
CB02366_sgcA2	192	dTDP-4-dehydrorhamnose 3,5-epimerase	SgcA2 (AAL06668)	91/93
CB02366_sgcD2	448	FAD-binding monooxygenase	SgcD2 (AAL06669)	97/97
CB02366_sgcA6	460	Glycosyl transferase	SgcA6 (AAL06670)	95/97
CB02366_sgcA	330	dNTP-glucose dehydratase	SgcA (AAL06671)	96/97
CB02366_sgcB	521	Transmembrane efflux protein	SgcB (AAL06672)	95/96
CB02366_sgcH	436	Coenzyme F390 synthase-like protein	SgcH (AAL06673)	99/99
CB02366_sgcC	527	Chlorophenol-4-monooxygenase	SgcC (AAL06674)	97/98
CB02366_sgcI	278	Hydrolase	SgcI (AAL06675)	97/97
CB02366_sgcJ	143	Unknown	SgcJ (AAL06676)	96/97
CB02366_sgcK	441	Citrate/shikimate transporter	SgcK (AAL06677)	96/98
CB02366_sgcC5	460	C-domain type II peptide synthetase	SgcC5 (AAL06678)	96/98
CB02366_sgcC2	93	Type II PCP	SgcC2 (AAL06679)	93/97
CB02366_sgcC4	539	MIO-dependent tyrosine 2,3-aminomutase	SgcC4 (AAL06680)	98/99
CB02366_sgcC1	882	A-domain type II peptide synthetase	SgcC1 (AAL06681)	83/86
CB02366_sgcB4	702	Antibiotic transporter	SgcB4 (AAL06682)	97/98
CB02366_sgcD4	349	*O*-methyltransferase	SgcD4 (AAL06683)	94/95
CB02366_sgcD3	420	Cytochrome P450 hydroxylase	SgcD3 (AAL06684)	93/95
CB02366_sgcL	393	Oxidoreductase	SgcL (AAL06685)	97/98
CB02366_sgcM	342	Unknown	SgcM (AAL06686)	89/90
CB02366_sgcN	306	Oxidoreductase	SgcN (AAL06687)	92/94
CB02366_sgcO	183	Alkylhydroperoxidase	SgcO (WP_010056303)	91/95
CB02366_sgcP	205	Hypothetical protein	SgcP (AAL06688)	91/94
CB02366_sgcR	413	Regulator	SgcR (AAL06689)	95/96
CB02366_sgcQ	327	Oxidase	SgcQ (AAL06690)	87/90
CB02366_sgcE11	267	Unknown	SgcE11 (AAL06691)	94/98
CB02366_sgcE10	156	Type II thioesterase	SgcE10 (AAL06692)	97/97
CB02366_sgcE9	551	Oxidoreductase	SgcE9 (AAL06693)	98/99
CB02366_sgcE8	196	Unknown	SgcE8 (AAL06694)	96/97
CB02366_sgcR1	359	Regulatory protein	SgcR1 (AAL06695)	93/94
CB02366_sgcR2	284	AraC family transcriptional regulator	SgcR2 (AAL06696)	92/96
CB02366_sgcE7	449	Cytochrome P450	SgcE7 (AAL06697)	93/94
CB02366_sgcE6	182	Flavin reductase	SgcE6 (AAL06698)	94/95
CB02366_sgcE	1939	Enediyne polyketide synthase	SgcE (AAL06699)	94/96
CB02366_sgcE5	376	Unknown	SgcE5 (AAL06700)	88/91
CB02366_sgcE4	622	Unknown	SgcE4 (AAL06701)	95/98

CB02366_sgcE3	325	Unknown	SgcE3 (AAL06702)	95/98
CB02366_sgcE2	314	Unknown	SgcE2 (AAL06703)	90/93
CB02366_sgcE1	147	HxlR family transcriptional regulator	SgcE1 (AAL06704)	95/95
CB02366_sgcS	201	Unknown	SgcS (AAL06705)	90/94
CB02366_sgcT	156	Unknown	SgcT (AAL06706)	84/90
CB02366_sgcR3	395	Transcriptional regulator	SgcR3 (AAL06707)	94/96

[a]*orf(-1)* and *orf(+1)* are predicted to represent the upstream and downstream boundaries of the C-1027 gene cluster.
[b]Number of amino acids.
[c]Also see Figure 3 and Figure S3 for the organization of the C-1027 gene cluster.

Table S3-2. Related to Figures 2, 4, 5. Predicted functions of ORFs in the *ucm* biosynthetic gene cluster from *S. uncialis* DCA2648

gene[a]	aa[b]	putative function[c]	protein homologue	% identity/ % similarity
orf (-1)	354	5-Methyltetrahydropteroyltriglutamate-homocysteine methyltransferase	M878_32615 (EST23768)	87/93
ucmF	217	Unknown	DynORF15 (ACB47058)	78/85
ucmD	439	PBS lyase HEAT-like repeat protein	DynORF16 (ACB47059)	79/86
ucmC	422	Ketone reductase	DynORF17 (ACB47060)	47/57
ucmR3	436	Unknown	DynR3 (ACB47054)	46/57
ucmT2	544	MFS transporter	SCLAV_p1343 (EFG04827)	39/52
ucmE10	144	Type II thioesterase	DynE7 (ACB47049)	51/65
ucmE	1950	Enediyne polyketide synthase	SgcE (AAL06699)	46/57
ucmE5	333	Unknown	DynT3 (ACB47047)	54/69
ucmE4	649	Unknown	DynU14 (ACB47046)	44/55
ucmE3	327	Unknown	DynU15 (ACB47045)	41/49
ucmS3	119	Glyoxalase/bleomycin resistance protein/dioxygenase	DynE15 (ACB47077)	50/64
ucmR4	178	AraC family transcriptional regulator	NCAST_20_03280 (GAD83759)	63/77
ucmS2	135	Glyoxalase/bleomycin resistance protein/dioxygenase	M877_16725 (EST27455)	54/70
ucmT1	554	Tetracenomycin C resistance and export protein	SgcB (AAL06672)	33/49
ucmS1	126	Glyoxalase/bleomycin resistance protein/dioxygenase	DynE15 (ACB47077)	41/52
ucmR2	442	Unknown	Dynorf18 (ACB47063)	56/68
ucmP	380	FAD-dependent oxidoreductase	DynE13 (ACB47064)	50/59
ucmO	173	Putative hydroxylase	DynA2 (ACB47066)	75/81
ucmN	158	Putative hydroxylase	DynA1 (ACB47065)	63/78
ucmM	340	Rieske (2Fe-2S) iron-sulfur domain protein	P354_01035 (EXU92880)	35/44
ucmK2	479	Hydrolase	DynA4 (ACB47068)	51/65
ucmK1	503	Hydrolase	DynA4 (ACB47068)	52/66
ucmJ	356	SAM-dependent methyltransferase	DynA5 (ACB47069)	51/62
ucmR1	235	Unknown	DynU8 (ACB47051)	45/60
ucmI	263	Oxidoreductase	KedU16 (AFV52172)	35/46
ucmG	736	Unknown +<br>SAM-dependent methyltransferase	DynORF17 (ACB47057)<br>DynORF28 (ACB47069)	54/62<br>43/53
ucmB	296	Enediyne self-sacrifice resistance protein	DynU16 (ACB47061)	47/59
ucmR7	281	AraC family transcriptional regulator	DynR7 (ACB47062)	42/51
orf(+1)	172	Pyridoxamine 5'-phosphate oxidase	SBD_5792 (EMF52716)	98/99

[a]*orf(-1)* and *orf(+1)* are predicted to represent the upstream and downstream boundaries of the *ucm* gene cluster.
[b]Number of amino acids.
[c]Also see Figures 5A, 5B for the organization of the *ucm* gene cluster.

Table S3-3. Related to Figures 2, 4, 5. Predicted functions of ORFs in the *tnm* biosynthetic gene cluster from *Streptomyces.* sp. CB03234 and comparison of the *tnm* gene cluster with the *ucm* gene cluster from *S. uncialis* DCA2648

tnm gene cluster			homologue in *ucm* cluster			homologue in NCBI database	
gene[a]	aa[b]	putative function[c]	gene[c]	aa[b]	% identity/ % similarity	protein homologue	% identity/ % similarity
orf(-1)	565	ATP-dependent DNA helicase RecQ				SHJG_7340 (AEY92607)	66/75
tnmE6	166	Flavin reductase				SpoE6 (ABP55139)	39/55
tnmR7	304	AraC family transcriptional regulator	*ucmR7*	281	76/80	DynR7 (ACB47062)	44/53
tnmB	303	Enediyne self-sacrifice resistance protein	*ucmB*	296	71/78	DynU16 (ACB47061)	51/62
tnmR3	428	Unknown	*ucmR3*	436	69/78	DynR3 (ACB47054)	47/59
tnmC	457	Ketone reductase	*ucmC*	422	44/51	DynORF17 (ACB47060)	40/48
tnmD	440	PBS lyase HEAT-like repeat protein	*ucmD*	439	89/92	DynORF16 (ACB47059)	82/90
tnmF	216	Unknown	*ucmF*	217	84/89	DynORF15 (ACB47058)	79/87
tnmG	737	Unknown + Methyltransferase	*ucmG*	736	78/83	DynORF14 (ACB47057)<br>DynA5 (ACB47069)	54/62<br>44/54
tnmH	344	*O*-Methyltransferase				SgcD4 (AAL06683)	39/55
tnmE10	147	Type II thioesterase	*ucmE10*	144	50/61	DynE7 (ACB47049)	59/70
tnmE	2109	Enediyne polyketide synthase	*ucmE*	1950	45/54	DynE8 (ACB47048)	50/58
tnmE5	336	Unknown	*ucmE5*	333	50/64	DynT3 (ACB47047)	60/70
tnmE4	652	Unknown	*ucmE4*	649	46/57	DynU14 (ACB47046)	53/64
tnmE3	322	Unknown	*ucmE3*	322	43/51	DynU15 (ACB47045)	44/52
tnmI	279	Oxidoreductase	*ucmI*	263	56/70	KedU16 (AFV52172)	33/47
tnmR1	247	HxlR family transcriptional regulator	*ucmR1*	235	45/60	DynU8 (ACB47051)	42/57
tnmJ	370	SAM-dependent methyltransferase	*ucmJ*	356	70/80	DynA5 (ACB47069)	49/62
tnmK1	484	Hydrolase	*ucmK1*	503	76/82	DynA4 (ACB47068)	53/67
tnmK2	489	Hydrolase	*ucmK2*	479	82/88	DynA4 (ACB47068)	52/66
tnmL	420	Cytochrome P450 monooxygenase				DynE10 (ACB47071)	46/53
tnmM1	337	Rieske (2Fe-2S) iron-sulfur domain protein	*ucmM*	340	74/83	P354_01035 (EXU92880)	29/42
tnmM2	339	Rieske (2Fe-2S) iron-sulfur domain protein	*ucmM*	340	68/75	Nos7107_5103 (AFY45618)	31/48
tnmN	146	Putative hydroxylase	*ucmN*	158	84/92	DynA1 (ACB47065)	62/78
tnmO	162	Putative hydroxylase	*ucmO*	173	88/95	DynA2 (ACB47066)	73/80
tnmP	387	FAD-dependent oxidoreductase	*ucmP*	380	75/81	DynE13 (ACB47064)	50/60
tnmR2	426	Putative regulator	*ucmR2*	442	82/86	DynORF18 (ACB47063)	54/65
tnmQ	189	Hypothetical protein				BN6_83400 (CCH35557)	59/70
tnmS1	126	Glyoxalase	*ucmS1*	126	90/95	DynE15 (ACB47077)	42/52
tnmT1	555	Transporter	*ucmT1*	554	76/82	CynR5 (AGO97174)	31/49
tnmS2	135	Glyoxalase	*ucmS2*	135	83/91	DynE15 (ACB47077)	43/50
tnmR4	194	AraC family transcriptional regulator	*ucmR4*	178	77/81	AMETH_2959 (AIJ23051)	56/70
tnmT2	568	MFS transporter	*ucmT2*	544	53/65	M878_32555 (EST23904)	52/65
tnmS3	124	Glyoxalase	*ucmS3*	119	82/88	DynE15 (ACB47077)	52/66
orf(+1)	237	Hypothetical protein				H114_11836 (EMF29004)	47/60

[a]*orf(-1)* and *orf(+1)* are predicted to represent the upstream and downstream boundaries of the *tnm* gene cluster.
[b]Number of amino acids.
[c]Also see Figures 5A, 5B for the organization of the *ucm* and *tnm* gene clusters.
