# Supplementary material for: Strain Prioritization and Genome Mining for Enediyne Natural Products
Source: mBio. 2016 Dec 20;7(6):e02104-16. doi: 10.1128/mBio.02104-16 (PMC5181780; doi:10.1128/mBio.02104-16)
Supplement: Table S4 — Annotation of the 29 enediyne gene clusters from the representative hits. [file mbo006163128st4.pdf]

Table S4-1. Related to Figures 2, 4. Predicted functions of ORFs in the enediyne biosynthetic gene cluster from *Streptomyces* sp. CB02009

gene[a]	aa[b]	putative function[c]	protein homologue	% identity/ % similarity
CB02009_orf(-1)	410	NikS protein	SSFG_07672 (EFE72437)	84/87
CB02009_orf1	496	Transmembrane efflux protein	NcsA2 (AAM77999)	32/46
CB02009_orf2	412	Cytochrome P450	SgcD3 (AAL06684)	35/46
CB02009_orf3	343	Chorismatase	FkbO (pdb|4BPS)	46/56
CB02009_orf4	495	AMP-dependent synthetase	NcsB1 (AAM77984)	25/37
CB02009_orf5	537	MIO-dependent tyrosine 2,3-aminomutase	SgcC4 (AAL06680)	81/89
CB02009_orf6	559	MFS transporter	SgcB (AAL06672)	87/93
CB02009_orf7	148	Apoprotein	NcsA (AAM77994)	38/53
CB02009_orf8	461	Oxidoreductase	SpoT4 (ABP55175)	34/48
CB02009_orf9	511	Methyltransferase	SpoT6 (ABP55177)	50/63
CB02009_F	389	Epoxide hydrolase	SgcF (AAL06662))	67/78
CB02009_orf11	470	Glycerol phosphate ABC transporter	SgcB1 (AAL06653)	50/63
CB02009_orf12	435	Phenylacetate-CoA ligase	SgcH (AAL06673)	82/90
CB02009_orf13	215	Anthranilate synthase II	SgcD1 (AAL06663)	76/82
CB02009_orf14	492	2-Amino-4-deoxychorismate synthase	SgcD (AAL06664)	72/79
CB02009_orf15	478	Phenylacetyl-CoA ligase	SgcD5 (AAL06665)	78/83
CB02009_orf16	223	2-Amino-4-deoxychorismate dehydrogenase	SgcG (AAL06666)	84/91
CB02009_orf17	403	Cytochrome P450 hydroxylase	SgcD3 (AAL06684)	71/79
CB02009_orf18	335	*O*-Methyltransferase	SgcD4 (AAL06683)	74/83
CB02009_orf19	702	Antibiotic transporter	SgcB4 (AAL06682)	68/79
CB02009_orf20	262	Hydrolase	SgcI (AAL06675)	47/62
CB02009_orf21	432	3-*O*-Acyltransferase	SgcD6 (AAL06667)	41/59
CB02009_orf22	621	Amino acid adenylation domain	SpoT2 (ABP55169)	58/68
CB02009_orf23	72	MbtH protein	SSAG_05958 (EDX26147)	51/64
CB02009_orf24	457	Type II condensation domain protein	MdpC5 (ABY66007)	45/59
CB02009_orf25	410	Hydroxylase	SpoORF17 (ABP55164)	69/79
CB02009_orf26	416	Cytochrome P450 monooxygenase	NcsB3 (AAM77997)	51/67
CB02009_orf27	179	MarR family transcriptional regulator	B446_16615 (AGS70139)	61/76
CB02009_orf28	448	FAD-binding monooxygenase	KedD2 (AFV52150)	65/75
CB02009_J	142	Unknown	KedJ (AFV52149)	61/76
CB02009_orf30	393	Oxidoreductase	SgcL (AAL06685)	67/79
CB02009_orf31	526	Monooxygenase	MdpC (ABY66026)	78/88
CB02009_orf32	549	Thiamine pyrophosphate-binding protein	Tter_2675 (ACZ43563)	45/60
CB02009_orf33	343	Methyltransferase	MppJ (AAU34201)	53/70
CB02009_orf34	300	Prephenate dehydrogenase	SSFG_07703 (EFE72468)	64/73
CB02009_E11	269	Unknown	NcsE11 (AAM78004)	70/81
CB02009_M	346	Unknown	SgcM (AAL06686)	55/64
CB02009_E9	555	Oxidoreductase	NcsE9 (AAM78005)	83/89
CB02009_E8	195	Unknown	NcsE8 (AAM78006)	78/87
CB02009_orf39	360	Transcriptional regulator	NcsR6 (AAM78007)	60/73
CB02009_orf40	258	AraC family transcriptional regulator	NcsR5 (AAM78008)	66/75
CB02009_E7	449	Cytochrome P450	NcsE7 (AAM78009)	78/86
CB02009_E6	151	Flavin reductase	NcsE6 (AAM78010)	78/88
CB02009_E10	152	Type II thioesterase	NcsE10 (AAM78011)	84/89
CB02009_E	1955	Enediyne polyketide synthase	NcsE (AAM78012)	75/83
CB02009_E5	344	Unknown	NcsE5 (AAM78013)	73/82
CB02009_E4	636	Unknown	NcsE4 (AAM78014)	80/89
CB02009_E3	328	Unknown	NcsE3 (AAM78015)	76/83
CB02009_E2	325	Unknown	NcsE2 (AAM78016)	68/76
CB02009_E1	147	Unknown	NcsE1 (AAM78017)	73/85
CB02009_orf50	402	Transcriptional regulator	NcsR7 (AAM78019)	60/72

CB02009_orf51	211	TetR family transcriptional regulator	NcsR3 (AAM78020)	50/65
CB02009_orf52	243	Short chain dehydrogenase	KedU16 (AFV52172)	35/47
CB02009_orf53	240	Haloacid dehalogenase-like hydrolase	SgvC (AGN74907)	61/72
CB02009_orf54	206	Butyrolactone receptor	NcsR2 (AAM78022)	50/67
CB02009_orf55	316	Gama-butyrolactone biosynthetic protein	NcsR1 (AAM78023)	61/77
CB02009_orf56	506	Hypothetical protein	SZN_11478 (EGX59686)	64/71
CB02009_orf57	217	MerR family transcriptional regulator	Spoorf2 (ABP55136)	39/52
CB02009_orf58	280	Unknown	NcsORF55 (AAM78024)	69/83
CB02009_orf(+1)	395	Plasmid partitioning protein	ParB2 (EDY46146)	52/63

[a]*orf(-1)* and *orf(+1)* are predicted to represent the upstream and downstream boundaries of the enediyne gene cluster.
[b]Number of amino acids.
[c]Also see Figure 4 and Figure 2C for the organization of the enediyne gene cluster.

Table S4-2. Related to Figures 2, 4. Predicted functions of ORFs in the enediyne biosynthetic gene cluster from *Streptomyces* sp. CB02261

gene[a]	aa[b]	putative function[c]	protein homologue	% identity/ % similarity
CB02261_orf(-1)	110	Hypothetical protein	SSFG_07664 (EFE72429)	64/75
CB02261_orf1	146	Apoprotein	NcsA (AAM77994)	48/59
CB02261_orf2	515	Long-chain fatty acid-CoA ligase	SacglDRAFT_00879 (EIE97817)	45/55
CB02261_orf3	573	Thiamine pyrophosphate-binding protein	Amir_4859 (ACU38685)	51/60
CB02261_orf4	436	Aldehyde dehydrogenase	Amir_4858 (ACU38684)	53/61
CB02261_orf5	308	GCN5 family acetyltransferase	MCAG_03609 (EEP73282)	28/40
CB02261_orf6	416	Carboxylate-amine ligase	MPTA5024_14060 (ETK35455)	47/60
CB02261_orf7	441	Carboxylase	SSFG_07669 (EFE72434)	84/88
CB02261_orf8	244	Serine *O*-acetyltransferase	Sfla_6199 (ADW07579)	70/83
CB02261_orf9	320	Cysteine synthase	SSFG_07671 (EFE72436)	85/92
CB02261_orf10	406	NikS protein	SSFG_07672 (EFE72437)	86/90
CB02261_orf11	838	A-domain type II peptide synthetase	SgcC1 (AAL06681)	48/55
CB02261_orf12	538	MIO-dependent tyrosine 2,3-aminomutase	SgcC4 (AAL06680)	87/92
CB02261_orf13	509	Methyltransferase type 11	SpoT6 (ABP55177)	53/64
CB02261_orf14	393	Oxidoreductase	SgcL (AAL06685)	62/72
CB02261_orf15	749	Cytochrome P450 and Zinc-containing alcohol dehydrogenase	SpoT9 (ABP55168) and SpoT5 (ABP55167)	73/83<br>66/81
CB02261_orf16	277	Methyltransferase type 11	SSFG_07678 (EFE72443)	89/92
CB02261_orf17	119	Hypothetical protein	Spoorf16 (ABP55163)	54/70
CB02261_orf18	467	Type II condensation domain protein	MdpC5 (ABY66007)	50/64
CB02261_orf19	91	Type II PCP domain protein	MdpC2 (ABY66003)	53/65
CB02261_orf20	536	Monooxygenase	MdpC (ABY66026)	82/91
CB02261_orf21	453	Glycerol phosphate ABC transporter	SgcB1 (AAL06653)	49/64
CB02261_orf22	238	Hypothetical protein	SSFG_07684 (EFE72449)	81/85
CB02261_orf23	260	Short-chain dehydrogenase	SSFG_07685 (EFE72450)	89/94
CB02261_orf24	448	FAD-linked oxidase	SpoT4 (ABP55175)	35/50
CB02261_orf25	421	Acyl-CoA dehydrogenase	Spoorf17 (ABP55164)	69/78
CB02261_orf26	460	Oxidoreductase	SpoT4 (ABP55175)	33/49
CB02261_orf27	371	Esterase	SSFG_07689 (EFE72454)	85/91
CB02261_orf28	538	MFS transporter	SgcB (AAL06672)	47/65
CB02261_orf29	222	2-Amino-4-deoxychorismate dehydrogenase	SgcG (AAL06666)	82/87
CB02261_orf30	477	Phenylacetyl-CoA ligase	SgcD5 (AAL06665)	76/82

CB02261_orf31	491	2-Amino-4-deoxychorismate synthase	SgcD (AAL06664)	66/76
CB02261_orf32	218	Anthranilate synthase II	SgcD1 (AAL06663)	74/84
CB02261_orf33	332	Alcohol dehydrogenase	MdpC8 (ABY66028)	84/91
CB02261_orf34	436	Phenylacetate-CoA ligase	SgcH (AAL06673)	79/87
CB02261_orf35	446	3-*O*-Acyltransferase	SgcD6 (AAL06667)	43/62
CB02261_F	388	Epoxide hydrolase	SgcF (AAL06662)	73/83
CB02261_orf37	466	FAD-binding monooxygenase	MdpL (ABY66029)	63/72
CB02261_J	139	Unknown	MdpJ (ABY66022)	59/76
CB02261_orf39	502	Oxidoreductase	SpoT4 (ABP55175)	29/41
CB02261_orf40	298	Prephenate dehydrogenase	SSFG_07702 (EFE72468)	64/75
CB02261_E11	269	Unknown	NcsE11 (AAM78004)	71/81
CB02261_M	347	Unknown	SgcM (AAL06686)	54/64
CB02261_E9	476	Oxidoreductase	NcsE9 (AAM78005)	83/90
CB02261_E8	195	Unknown	NcsE8 (AAM78006)	74/82
CB02261_orf45	371	Transcriptional regulator	NcsR6 (AAM78007)	59/72
CB02261_orf46	255	AraC family transcriptional regulator	NcsR5 (AAM78008)	67/76
CB02261_E7	449	Cytochrome P450	NcsE7 (AAM78009)	79/88
CB02261_E6	181	Flavin reductase	NcsE6 (AAM78010)	74/85
CB02261_E10	152	Type II thioesterase	NcsE10 (AAM78011)	84/90
CB02261_E	1958	Enediyne polyketide synthase	NcsE (AAM78012)	74/82
CB02261_E5	347	Unknown	NcsE5 (AAM78013)	74/83
CB02261_E4	636	Unknown	NcsE4 (AAM78914)	77/88
CB02261_E3	328	Unknown	NcsE3 (AAM78015)	75/84
CB02261_E2	326	Unknown	NcsE2 (AAM78016)	65/74
CB02261_E1	147	Unknown	NcsE1 (AAM78017)	73/83
CB02261_orf56	392	Transcriptional regulator	NcsR7 (AAM78019)	60/72
CB02261_orf57	213	Gamma-butyrolactone receptor protein	NcsR3 (AAM78020)	49/66
CB02261_orf58	319	Autoregulator biosynthesis protein	NcsC2 (AAM78021)	55/68
CB02261_orf59	209	Butyrolactone receptor	NcsR2 (AAM78022)	65/75
CB02261_orf60	316	Gama-butyrolactone biosynthetic protein	NcsR1 (AAM78023)	62/76
CB02261_orf61	496	Hypothetical protein	SSFG_07727 (EFE72492)	61/71
CB02261_orf62	221	MerR family transcriptional regulator	Spoorf2 (ABP55136)	35/48
CB02261_orf63	281	Unknown	NcsORF55 (AAM78024)	67/82
CB02261_orf(+1)	70	Hypothetical protein	SSDG_02131 (EDY63712)	90/91

[a]*orf(-1)* and *orf(+1*) are predicted to represent the upstream and downstream boundaries of the enediyne gene cluster.
[b]Number of amino acids.
[c]Also see Figure 4 and Figure 2C for the organization of the enediyne gene cluster.

Table S4-3. Related to Figures 2, 4. Predicted functions of ORFs in the enediyne biosynthetic gene cluster from *Streptomyces* sp. CB02130

gene[a]	aa[b]	putative function[c]	protein homologue	% identity/ % similarity
CB02130_orf(-1)	410	NikS protein	SSFG_07672 (EFE72437)	81/86
CB02130_orf1	495	MFS transporter	NcsA2 (AAM77999)	32/47
CB02130_orf2	431	Cytochrome P450	SgcD3 (AAL06684)	33/44
CB02130_orf3	344	Chorismatase	Cuv10 (AGO98693)	49/59
CB02130_orf4	494	AMP-dependent synthetase and ligase	MdpB2 (ABY66018)	30/38
CB02130_orf5	537	MIO-dependent tyrosine 2,3-aminomutase	SgcC4 (AAL06680)	80/89
CB02130_orf6	538	MFS transporter	Spoorf15 (ABP55162)	54/72
CB02130_orf7	147	Apoprotein	CagA (AAL06658)	39/55
CB02130_orf8	466	Oxidoreductase	SpoT4 (ABP55175)	32/45

CB02130_orf9	510	Methyltransferase	SpoT6 (ABP55177)	49/62
CB02130_F	389	Epoxide hydrolase	KedF (AFV52152)	77/84
CB02130_orf11	469	Phosphate ABC transporter	SgcB1 (AAL06653)	50/62
CB02130_orf12	459	Coenzyme F390 synthase-like protein	SgcH (AAL06673)	82/89
CB02130_orf13	211	Anthranilate synthase II	SgcDI (AAL06663)	77/83
CB02130_orf14	493	2-Amino-4-deoxychorismate synthase	SgcD (AAL06664)	71/79
CB02130_orf15	478	Phenylacetyl-CoA ligase	SgcD5 (AAL06665)	77/83
CB02130_orf16	223	2-Amino-4-deoxychorismate dehydrogenase	SgcG (AAL06666)	86/90
CB02130_orf17	412	Cytochrome P450 hydroxylase	SgcD3 (AAL06684)	71/79
CB02130_orf18	335	*O*-Methyltransferase	SgcD4 (AAL06683)	74/84
CB02130_orf19	702	Antibiotic transporter	SgcB4 (AAL06682)	68/79
CB02130_orf20	262	Hydrolase	SgcI (AAL06675)	46/62
CB02130_orf21	432	3-*O*-Acyltransferase	SgcD6 (AAL06667)	40/59
CB02130_orf22	605	Amino acid adenylation domain	SpoT2 (ABP55169)	59/70
CB02130_orf23	456	C-domain type II peptide synthetase	SgcC5 (AAL06678)	44/60
CB02130_orf24	411	Acyl-CoA dehydrogenase	Spoorf17 (ABP55164)	68/77
CB02130_orf25	416	Cytochrome P450 hydroxylase	NcsB3 (AAM77997)	52/68
CB02130_orf26	448	FAD-dependent monooxygenase	KedD2 (AFV52150)	63/74
CB02130_J	140	Unknown	KedJ (AFV52149)	59/76
CB02130_orf28	393	Oxidoreductase	SgcL (AAL06685)	66/77
CB02130_orf29	526	Chlorophenol-4-monooxygenase	SgcC (AAL06674)	75/86
CB02130_orf30	553	Hypothetical protein	WP_027735081	78/84
CB02130_orf31	332	Methyltransferase	MppJ (AAU34201)	55/70
CB02130_orf32	298	Hypothetical protein	Sros_6984 (ACZ89684)	50/61
CB02130_E11	265	Unknown	NcsE11 (AAM78004)	69/82
CB02130_M	344	Unknown	SgcM (AAL06686)	56/65
CB02130_E9	555	Oxidoreductase	NcsE9 (AAM78005)	81/88
CB02130_E8	195	Unknown	NcsE8 (AAM78006)	75/84
CB02130_orf37	357	Transcriptional regulator	NcsR6 (AAM78007)	57/71
CB02130_orf38	260	AraC family transcriptional regulator	NcsR5 (AAM78008)	66/76
CB02130_E7	447	Cytochrome P450	NcsE7 (AAM78009)	78/85
CB02130_E6	182	Flavin reductase	NcsE6 (AAM78010)	71/78
CB02130_E10	152	Type II thioesterase	NcsE10 (AAM78011)	84/90
CB02130_E	1957	Enediyne polyketide synthase	NcsE (AAM78012)	75/83
CB02130_E5	350	Unknown	NvsE5 (AAM78013)	76/85
CB02130_E4	607	Unknown	NcsE4 (AAM78014)	78/89
CB02130_E3	307	Unknown	NcsE3 (AAM78015)	73/83
CB02130_E2	325	Unknown	NcsE2 (AAM78016)	65/74
CB02130_E1	147	HxlR family transcriptional regulator	NcsE1 (AAM78017)	71/84
CB02130_orf48	404	Transcriptional regulator	NcsR7 (AAM78019)	59/71
CB02130_orf49	216	TetR family transcriptional regulator	NcsR3 (AAM78020)	48/62
CB02130_orf50	316	Autoregulator biosynthesis protein	NcsC2 (AAM78021)	54/68
CB02130_orf51	208	Butyrolactone receptor	NcsR2 (AAM78022)	62/73
CB02130_orf52	293	Gama-butyrolactone biosynthetic protein	NcsR1 (AAM78023)	61/76
CB02130_orf(+1)	445	Hypothetical protein	SSFG_07727 (EFE72492)	60/69

[a]*orf(-1)* and *orf(+1)* are predicted to represent the upstream and downstream boundaries of the enediyne gene cluster.
[b]Number of amino acids.
[c]Also see Figure 4 and Figure 2C for the organization of the enediyne gene cluster.

Table S4-4. Related to Figures 2, 4. Predicted functions of ORFs in the enediyne biosynthetic gene cluster from *Streptomyces* sp. CB00455

gene[a]	aa[b]	putative function[c]	protein homologue	% identity/

				% similarity
CB00455_orf(-1)	286	Hypothetical protein	HMPREF1486_03680 (EPD93824)	56/71
CB00455_orf1	427	Major facilitator family transporter	SSFG_07655 (EFE72420)	74/83
CB00455_orf2	411	Phosphoribosylglycinamide synthetase	SSNG_06428 (EFL19176)	67/76
CB00455_orf3	414	Carboxylase	SSNG_06429 (EFL19177)	68/76
CB00455_M	347	Unknown	SgcM (AAL06686)	60/71
CB00455_orf5	497	Bilirubin oxidase	SACTE_6357 (AEN14128)	51/66
CB00455_orf6	275	*O*-Methyltransferase	LkmG (BAC76486)	36/52
CB00455_orf7	232	Unknown	Spoorf22 (ABP55176)	33/50
CB00455_orf8	539	MFS transporter	Spoorf15 (ABP55162)	55/72
CB00455_orf9	541	Major facilitator MFS1 protein	SgcB (AAL06672)	49/65
CB00455_orf10	374	Acyltransferase	CynA4 (AGO97170)	36/50
CB00455_orf11	356	Hypothetical protein	Cseg_0869 (ADG09375)	44/58
CB00455_orf12	461	Oxidoreductase	SpoT4 (ABP55175)	29/44
CB00455_orf13	518	Methyltransferase	SpoT6 (ABP55177)	50/62
CB00455_orf14	256	Type II thioesterase	KedU43 (AFV52205)	43/54
CB00455_orf15	416	3-*O*-Acyltransferase	SgcD6 (AAL06667)	40/57
CB00455_orf16	158	ATPase	Cynorf32 (AGO97175)	37/48
CB00455_orf17	113	DGPFAETKE family protein	Spoorf12 (ABP55159)	32/54
CB00455_orf18	262	Short chain dehydrogenase	KedU16 (AFV52172)	36/51
CB00455_orf19	387	Acyl-CoA dehydrogenase	KedU41 (AFV52203)	33/50
CB00455_orf20	559	AMP-binding protein	NcsB2 (AAM77987)	75/84
CB00455_orf21	290	Luciferase	MPTA5024_08200 (ETK36646)	55/69
CB00455_orf22	506	FAD-linked oxidase	TrdL (ADY38530)	54/68
CB00455_orf23	549	Asparagine synthetase	Psm3C (BAF68992)	52/70
CB00455_orf24	613	Nonribosomal peptide synthetase	SgcC1 (AAL06681)	34/46
CB00455_orf25	731	Hypothetical protein	WP_020555677	48/57
CB00455_orf26	237	Aspartate racemase	SVEN_6253 (CCA59539)	61/69
CB00455_orf27	447	Glycerol phosphate ABC transporter	SgcB1 (AAL06653)	50/63
CB00455_orf28	743	Molybdopterin-binding oxidoreductase	STTU_4313 (EGJ77102)	63/75
CB00455_orf29	415	Acyl-CoA dehydrogenase	Spoorf17 (ABP55164)	67/78
CB00455_orf30	482	Hydroxylase	MdpH (ABY66008)	56/71
CB00455_orf31	890	A-domain type II peptide synthetase	SgcC1 (AAL06681)	41/50
CB00455_orf32	541	MIO-dependent tyrosine 2,3-aminomutase	SgcC4 (AAL06680)	77/87
CB00455_orf33	445	C-domain type II peptide synthetase	SgcC5 (AAL06678)	51/66
CB00455_orf34	528	Monooxygenase	SgcC (AAL06674)	76/85
CB00455_orf35	396	Oxidoreductase	SgcL (AAL06685)	66/78
CB00455_J	143	Unknown	SgcJ (AAL06676)	57/75
CB00455_orf37	418	Cytochrome P450 monooxygenase	KedN3 (AFV52151)	78/86
CB00455_orf38	289	Phytanoyl-CoA dioxygenase	STTU_4295 (EGJ77084)	63/75
CB00455_orf39	86	Type II PCP domain protein	MdpC2 (ABY66003)	43/68
CB00455_orf40	411	NikS protein	SSAG_05939 (EDX26128)	78/84
CB00455_orf41	344	Cysteine synthase	SSAG_05938 (EDX26127)	79/87
CB00455_orf42	197	Serine *O*-acetyltransferase	SSFG_07670 (EFE72435)	77/85
CB00455_orf43	196	Prephenate dehydrogenase	SSFG_07703 (EFE72468)	52/61
CB00455_E11	267	Unknown	NcsE11 (AAM78004)	74/82
CB00455_E9	553	Oxidoreductase	NcsE9 (AAM78005)	82/88
CB00455_E8	195	Unknown	NcsE8 (AAM78006)	77/84
CB00455_orf47	366	Transcriptional regulator	NcsR6 (AAM78007)	58/70
CB00455_orf48	256	AraC family transcriptional regulator	NcsR5 (AAM78008)	69/79
CB00455_E7	449	Cytochrome P450	NcsE7 (AAM78009)	77/85
CB00455_E6	138	Flavin reductase	NcsE6 (AAM78010)	73/83
CB00455_E10	152	Type II thioesterase	NcsE10 (AAM78011)	83/90
CB00455_E	1972	Enediyne polyketide synthase	NcsE (AAM78012)	75/83
CB00455_E5	371	Unknown	NcsE5 (AAM78013)	73/82

CB00455_E4	617	Unknown	NcsE4 (AAM78014)	75/86
CB00455_E3	327	Unknown	NcsE3 (AAM78015)	71/82
CB00455_E2	329	Unknown	NcsE2 (AAM78016)	70/76
CB00455_E1	147	Unknown	NcsE1 (AAM78017)	73/87
CB00455_orf58	400	Transcriptional regulator	NcsR7 (AAM78019)	61/74
CB00455_orf59	210	TetR family transcriptional regulator	NcsR3 (AAM78020)	51/68
CB00455_orf60	318	Autoregulator biosynthesis enzyme	NcsC2 (AAM78021)	52/64
CB00455_orf61	208	Butyrolactone receptor	NcsR2 (AAM78022)	58.72
CB00455_orf62	315	Gama-butyrolactone biosynthetic protein	NcsR1 (AAM78023)	62/78
CB00455_orf63	571	Hypothetical protein	SSFG_07727 (EFE72492)	46/56
CB00455_orf64	292	Unknown	NcsORF55 (AAM78024)	63/76
CB00455_orf(+1)	434	Predicted protein	SSMG_03914 (EFL08243)	39/52

[a]*orf(-1)* and *orf(+1)* are predicted to represent the upstream and downstream boundaries of the enediyne gene cluster.
[b]Number of amino acids.
[c]Also see Figure 4 and Figure 2C for the organization of the enediyne gene cluster.

Table S4-5. Related to Figures 2, 4. Predicted functions of ORFs in the enediyne biosynthetic gene cluster from *Streptomyces* sp. CB03578

gene[a]	aa[b]	putative function[c]	protein homologue	% identity/ % similarity
CB03578_orf(-1)	410	NikS protein	WP_008743142	99/99
CB03578_orf1	497	MFS transporter	NcsA2 (AAM77999)	33/47
CB03578_orf2	343	Imine deaminase	WP_008743144	99/99
CB03578_orf3	494	Acyl-CoA synthetase	WP_037794763	100/100
CB03578_orf4	541	MIO-dependent tyrosine 2,3-aminomutase	SgcC4 (AAL06680)	80/89
CB03578_orf5	523	MFS transporter	Spoorf15 (ABP55162)	54/72
CB03578_orf6	399	Kynureninase	WP_037794765	100/100
CB03578_orf7	263	Tryptophan 2,3-dioxygenase	WP_008743149	99/99
CB03578_orf8	514	AMP-dependent synthetase and ligase	KedU37 (AFV52199)	31/42
CB03578_orf9	148	Apoprotein	NcsA (AAM77994)	41/49
CB03578_orf10	456	FAD-linked oxidase	SpoT4 (ABP55175)	32/44
CB03578_orf11	515	Methyltransferase	SpoT6 (ABP55177)	51/65
CB03578_F	390	Epoxide hydrolase	KedF (AFV52152)	76/83
CB03578_orf13	468	Phosphate ABC transporter	SgcB1 (AAL06653)	50/63
CB03578_orf14	261	Hydrolase	SgcI (AAL06675)	47/59
CB03578_orf15	444	3-*O*-Acyltransferase	SgcD6 (AAL06667)	42/60
CB03578_orf16	303	N-acetyltransferase protein	WP_047961327	99/99
CB03578_orf17	602	Amino acid adenylation domain	SpoT2 (ABP55169)	59/71
CB03578_orf18	73	MbtH	WP_008743161	100/100
CB03578_orf19	459	Condensation domain protein	SpoT10 (ABP55165)	39/58
CB03578_orf20	411	Acyl-CoA dehydrogenase	SpoOrf17 (ABP55164)	65/76
CB03578_orf21	415	Cytochrome P450 monooxygenase	KedN3 (AFV52151)	76/84
CB03578_orf22	447	FAD-dependent monooxygenase	KedD2 (AFV52150)	63/75
CB03578_J	142	Unknown	KedJ (AFV52149)	63/73
CB03578_orf24	393	Oxidoreductase	MdpD2 (ABY66021)	71/84
CB03578_orf25	529	Monooxygenase	MdpC3 (ABY66026)	78/87
CB03578_orf26	572	Thiamine pyrophosphate-binding protein	WP_037800491	99/99
CB03578_orf27	332	Methyltransferase	WP_047961328	99/100
CB03578_orf28	409	Cytochrome P450 hydroxylase	SgcD3 (AAL06684)	53/69
CB03578_orf29	337	*O*-methyltransferase	SgcD4 (AAL06683)	55/69
CB03578_orf30	297	Esterase	WP_047961330	99/99

CB03578_orf31	104	Hypothetical protein	WP_037800485	99/99
CB03578_orf32	299	Prephenate dehydrogenase	WP_037800482	99/98
CB03578_E11	272	Unknown	SgcE11 (AAL06691)	70/83
CB03578_M	357	Unknown	SgcM (AAL06686)	54/63
CB03578_E9	555	Oxidoreductase	SgcE9 (AAL06693)	86/92
CB03578_E8	199	Unknown	NcsE8 (AAM78006)	78/86
CB03578_orf37	384	Regulator	SgcR1 (AAL06695)	55/69
CB03578_orf38	258	AraC family transcriptional regulator	NcsR5 (AAM78008)	70/80
CB03578_E7	449	Cytochrome P450	NcsE7 (AAM78009)	77/86
CB03578_E6	181	Flavin reductase	NcsE6 (AAM78010)	73/80
CB03578_E10	152	Type II thioesterase	NcsE10 (AAM78011)	84/90
CB03578_E	1950	Enediyne polyketide synthase	NcsE (AAM78012)	75/83
CB03578_E5	378	Unknown	NcsE5 (AAM78013)	75/85
CB03578_E4	636	Unknown	NcsE4 (AAM78014)	75/86
CB03578_E3	328	Unknown	NcsE3 (AAM78015)	72/83
CB03578_E2	326	Unknown	NcsE2 (AAM78016)	70/78
CB03578_E1	147	Unknown	NcsE1 (AAM78017)	73/86
CB03578_orf48	401	Transcriptional regulator	NcsR7 (AAM78019)	63/76
CB03578_orf49	105	Hypothetical protein	SSAG_04940 (EDX25073)	99/99
CB03578_orf50	212	TetR family transcriptional regulator	NcsR3 (AAM78020)	51/66
CB03578_orf51	320	Autoregulator biosynthesis protein	NcsC2 (AAM78021)	58/71
CB03578_orf52	208	Butyrolactone receptor	NcsR2 (AAM78022)	68/81
CB03578_orf53	314	Gama-butyrolactone biosynthetic protein	NcsR1 (AAM78023)	61/76
CB03578_orf54	557	Hypothetical protein	WP_008742097	97/98
CB03578_(+1)	287	Unknown	NcsORF55 (AAM78024)	62/74

[a]*orf(-1)* and *orf(+1)* are predicted to represent the upstream and downstream boundaries of the enediyne gene cluster.
[b]Number of amino acids.
[c]Also see Figure 4 and Figure 2C for the organization of the enediyne gene cluster.

Table S4-6. Related to Figures 2, 4. Predicted functions of ORFs in the enediyne biosynthetic gene cluster from *Streptomyces* sp. CB01883

gene[a]	aa[b]	putative function[c]	protein homologue	% identity/ % similarity
CB01883_orf(-1)	410	NikS protein	SSFG_07672 (EFE72437)	84/88
CB01883_orf1	493	MFS transporter	NcsA2 (AAM77999)	31/46
CB01883_orf2	424	Cytochrome P450	KedU45 (AFV52207)	39/54
CB01883_orf3	342	Chorismatase	Cuv10 (AGO98693)	47/58
CB01883_orf4	494	Acyltransferase	MdpB2 (ABY66018)	29/39
CB01883_orf5	537	MIO-dependent tyrosine 2,3-aminomutase	SgcC4 (AAL06680)	79/88
CB01883_orf6	545	MFS transporter	Spoorf15 (ABP55162)	53/73
CB01883_orf7	146	Apoprotein	CagA (AAL06658)	34/52
CB01883_orf8	461	Oxidoreductase	SpoT4 (ABP55175)	34/47
CB01883_orf9	512	Methyltransferase	SpoT6 (ABP55177)	48/61
CB01883_F	389	Epoxide hydrolase	KedF (AFV52152)	77/84
CB01883_orf11	466	Glycerol phosphate ABC transporter	SgcB1 (AAL06653)	51/65
CB01883_orf12	435	Phenylacetate-CoA ligase	SgcH (AAL06673)	81/90
CB01883_orf13	217	Anthranilate synthase II	SgcD1 (AAL06663)	75/82
CB01883_orf14	492	2-Amino-4-deoxychorismate synthase	SgcD (AAL06664)	71/79
CB01883_orf15	480	Phenylacetyl CoA ligase	SgcD5 (AAL6665)	76/83
CB01883_orf16	223	2-Amino-4-deoxychorismate dehydrogenase	SgcG (AAL06666)	85/92
CB01883_orf17	414	Cytochrome P450 hydroxylase	SgcD3 (AAL06684)	71/79

CB01883_orf18	335	*O*-Methyltransferase	SgcD4 (AAL06683)	75/83
CB01883_orf19	700	Antibiotic transporter	SgcB4 (AAL06682)	66/77
CB01883_orf20	262	Hydrolase	SgcI (AAL06675)	45/63
CB01883_orf21	514	3-*O*-Acyltransferase	SgcD6 (AAL06667)	41/59
CB01883_orf22	589	NRPS A-PCP didomain	SpoT2 (ABP55169)	59/70
CB01883_orf23	69	MbtH protein	SSAG_05958 (EDX26147)	65/76
CB01883_orf24	457	C-domain type II peptide synthetase	SgcC5 (AAL06678)	44/59
CB01883_orf25	410	Acyl-CoA dehydrogenase	Spoorf17 (ABP55164)	70/78
CB01883_orf26	441	Cytochrome P450	NcsB3 (AAM77997)	51/67
CB01883_orf27	448	FAD-dependent monooxygenase	KedD2 (AFV52150)	67/76
CB01883_J	141	Unknown	SgcJ (AAL06676)	62/73
CB01883_orf29	393	Oxidoreductase	SgcL (AAL06685)	65/78
CB01883_orf30	526	Chlorophenol-4-monooxygenase	SgcC (AAL06674)	76/86
CB01883_orf31	549	Thiamine pyrophosphate-binding protein	SSAG_05966 (EDX26155)	75/80
CB01883_orf32	332	*O*-Methyltransferase family 2	H340_06661 (EMF01408)	61/72
CB01883_orf33	297	Prephenate dehydrogenase	MPTA5024_05410 (ETK37147)	52/59
CB01883_E11	269	Unknown	NcsE11 (AAM78004)	71/80
CB01883_M	346	Unknown	SgcM (AAL06686)	54/65
CB01883_E9	555	Oxidoreductase	NcsE9 (AAM78005)	83/89
CB01883_E8	195	Unknown	NcsE8 (AAM78006)	75/84
CB01883_orf38	358	Transcriptional regulator	NcsR6 (AAM78007)	60/72
CB01883_orf39	265	AraC family transcriptional regulator	NcsR5 (AAM78008)	66/77
CB01883_E7	440	Cytochrome P450	NcsE7 (AAM78009)	80/86
CB01883_E6	181	Flavin reductase	NcsE6 (AAM78010)	72/82
CB01883_E10	152	Type II thioesterase	NcsE10 (AAM78011)	86/90
CB01883_E	1961	Enediyne polyketide synthase	NcsE (AAM78012)	75/83
CB01883_E5	372	Unknown	NcsE5 (AAM78013)	72/80
CB01883_E4	636	Unknown	NcsE4 (AAM78014)	77/87
CB01883_E3	329	Unknown	NcsE3 (AAM78015)	74/84
CB01883_E2	325	Unknown	NcsE2 (AAM78016)	67/75
CB01883_E1	147	HxlR family transcriptional regulator	NcsE1 (AAM78017)	75/86
CB01883_orf49	401	Transcriptional regulator	NcsR7 (AAM78019)	59/74
CB01883_orf50	227	TetR family transcriptional regulator	NcsR3 (AAM78020)	53/67
CB01883_orf51	317	Autoregulator biosynthesis enzyme	NcsC2 (AAM78021)	68/76
CB01883_orf52	199	Butyrolactone receptor	NcsR2 (AAM78022)	65/79
CB01883_orf53	318	Gama-butyrolactone biosynthetic protein	NcsR1 (AAM78023)	58/74
CB01883_orf54	172	Hypothetical protein	WP_016828662	63/70
CB01883_orf55	102	Hypothetical protein	WP_026085629	53/60
CB01883_orf56	256	Unknown	NcsORF55 (AAM78024)	64/75
CB01883_orf(+1)	435	Plasmid partitioning protein	ParB1 (AIS02472)	62/69

[a]*orf(-1)* and *orf(+1)* are predicted to represent the upstream and downstream boundaries of the enediyne gene cluster.
[b]Number of amino acids.
[c]Also see Figure 4 and Figure 2C for the organization of the enediyne gene cluster.

Table S4-7. Related to Figures 2, 4. Predicted functions of ORFs in the enediyne biosynthetic gene cluster from *Streptomyces* sp. CB02400

gene[a]	aa[b]	putative function[c]	protein homologue	% identity/ % similarity
CB02400_orf(-1)	95	Valyl-tRNA synthetase	VM98_08460 (KJS56197)	81/86
CB02400_orf1	420	Cytochrome P450	SgcD3 (AAL06684)	31/44

CB02400_orf2	362	Chorismatase	Cuv10 (AGO98693)	51/64
CB02400_orf3	519	MIO-dependent tyrosine 2,3-aminomutase	SgcC4 (AAL06680)	70/82
CB02400_orf4	329	Unknown	CalU6 (AAM94767)	48/66
CB02400_orf5	93	Hypothetical protein	STRIP9103_09474 (EKX60173)	86/92
CB02400_orf6	133	Putative translation initiation inhibitor	SAMT0191 (CAI78120)	85/93
CB02400_orf7	127	HxlR family transcriptional regulator	SgcE1 (AAL06704)	34/50
CB02400_orf8	537	Major facilitator MFS1 protein	SgcB (AAL06672)	46/62
CB02400_orf9	148	Apoprotein	CagA (AAL06658)	40/57
CB02400_orf10	316	Short chain dehydrogenase	SgcN (AAL06687)	53/62
CB02400_orf11	180	Unknown	SgcO (WP_010056303)	66/75
CB02400_orf12	433	Citrate/shikimate transporter	SgcK (AAL06677)	63/77
CB02400_orf13	308	GCN5 family acetyltransferase	MCBG_03141 (EWM66008)	29/41
CB02400_orf14	466	FAD-dependent oxidoreductase	SpoT4 (ABP55175)	30/44
CB02400_orf15	510	Methyltransferase	SpoT6 (ABP55177)	50/64
CB02400_F	394	Epoxide hydrolase	SgcF (AAL06662)	67/76
CB02400_orf17	467	Glycerol phosphate ABC transporter	SgcB1 (AAL06653)	51/63
CB02400_orf18	214	Peptidase S15	T261_4505 (AJT66148)	39/54
CB02400_orf19	441	Phenylacetate-CoA ligase	SgcH (AAL06673)	83/91
CB02400_orf20	216	Anthranilate synthase II	SgcD1 (AAL06663)	76/86
CB02400_orf21	504	2-Amino-4-deoxychorismate synthase	SgcD (AAL0664)	72/77
CB02400_orf22	472	Phenylacetyl-CoA ligase	SgcD5 (AAL06665)	76/83
CB02400_orf23	241	2-Amino-4-deoxychorismate dehydrogenase	SgcG (AAL06666)	79/89
CB02400_orf24	412	Cytochrome P450 hydroxylase	SgcD3 (AAL06684)	67/77
CB02400_orf25	338	*O*-Methyltransferase	SgcD4 (AAL06683)	72/81
CB02400_orf26	186	Pyrimidine reductase	Cyaorf7 (AGO97196)	28/44
CB02400_orf27	233	Hypothetical protein	IX27_15600 (KFK88781)	82/87
CB02400_orf28	193	*N*-acetyltransferase GCN5	SAM23877_7239 (AKZ60282)	83/89
CB02400_orf29	172	Hypothetical protein	ADK35_01840 (KOG29462)	50/65
CB02400_orf30	310	Hypothetical protein	ML5_5948 (ADU11400)	40/51
CB02400_orf31	702	Antibiotic transporter	SgcB4 (AAL06682)	65/79
CB02400_orf32	261	Hydrolase	SgcI (AAL06675)	51/68
CB02400_orf33	434	3-*O*-Acyltransferase	SgcD6 (AAL06667)	38/57
CB02400_orf34	107	Hypothetical protein	M444_37705 (AKL71115)	60/72
CB02400_orf35	603	FAD-dependent oxidoreductase	SSFG_07663 (EFE72428)	69/77
CB02400_orf36	472	Dihydroorotase	StnX (AFW04592)	67/75
CB02400_orf37	359	Acyl-CoA dehydrogenase	KedU41 (AFV52203)	36/46
CB02400_orf38	377	Hypothetical protein	StnK5 (AFW04590)	51/62
CB02400_orf39	604	Asparagine synthase	M444_37680 (AKL71111)	77/83
CB02400_orf40	317	Cysteine synthase	ADK74_31310 (KOG38833)	78/85
CB02400_orf41	264	Aldolase	M271_07080 (AGP53038)	57/70
CB02400_orf42	286	Taurine dioxygenase	SSAG_06914 (EDX27123)	86/94
CB02400_orf43	423	Major facilitator family transporter	SSFG_07655 (EFE72420)	75/83
CB02400_orf44	335	Methyltransferase	DynO6 (ACB47081)	31/45
CB02400_orf45	551	Thiamine pyrophosphate-binding protein	SSAG_05966 (EDX26155)	71/77
CB02400_orf46	527	Monooxygenase	MdpC3 (ABY66026)	80/87
CB02400_orf47	393	Oxidoreductase	MdpD2 (ABY66021)	67/81
CB02400_J	143	Unknown	MdpJ (AAM77985)	51/70
CB02400_orf49	450	FAD-binding monooxygenase	NcsD2 (AAM78003)	55/70
CB02400_orf50	125	Fic family toxin-antitoxin system, toxin component	ABB07_00340 (AKJ08553)	90/94
CB02400_orf51	246	Hypothetical protein	SCATT_p00160 (AEW98209)	90/92
CB02400_orf52	324	GCN5 family acetyltransferase	ACH49_20030 (KMS77632)	92/94
CB02400_orf53	527	Alpha-ketoglutarate decarboxylase	SZN_00135 (EGX61722)	74/80
CB02400_orf54	415	Cytochrome P450 monooxygenase	KedN3 (AFV52151)	76/83
CB02400_orf55	412	Acyl-CoA dehydrogenase	Spoorf17 (ABP55164)	66/77

CB02400_orf56	469	C-domain type II peptide synthetase	SgcC5 (AAL06678)	42/58
CB02400_orf57	588	Amino acid adenylation domain	SpoT2 (ABP55169)	57/68
CB02400_orf58	410	NikS protein	SSAG_05939 (EDX26128)	82/88
CB02400_orf59	317	Cysteine synthase	SSFG_07671 (EFE72436)	81/88
CB02400_orf60	212	Serine *O*-acetyltransferase	SSFG_07670 (EFE72435)	77/82
CB02400_orf61	421	Carboxylase	SSFG_07669 (EFE72434)	80/85
CB02400_orf62	414	Phosphoribosylglycinamide synthetase	SSFG_07668 (EFE72433)	75/81
CB02400_orf63	296	Prephenate dehydrogenase	SSPG_04318 (EFD68678)	41/51
CB02400_E11	269	Unknown	NcsE11 (AAM78004)	70/80
CB02400_M	345	Unknown	SgcM (AAL06686)	55/62
CB02400_E9	557	Oxidoreductase	NcsE9 (AAM78005)	82/88
CB02400_E8	195	Unknown	NcsE8 (AAM78006)	78/85
CB02400_orf68	361	Regulator	SgcR1 (AAL06695)	63/70
CB02400_orf69	256	AraC family transcriptional regulator	SgcR2 (AAL06695)	59/70
CB02400_E7	449	Cytochrome P450	NcsE7 (AAM78009)	78/86
CB02400_E6	179	Flavin reductase	NcsE6 (AAM78010)	68/78
CB02400_E10	152	Type II thioesterase	NcsE10 (AAM78011)	84/91
CB02400_E	1978	Enediyne polyketide synthase	NcsE (AAM78012)	74/82
CB02400_E5	365	Unknown	NcsE5 (AAM78013)	74/83
CB02400_E4	616	Unknown	NcsE4 (AAM78014)	81/89
CB02400_E3	308	Unknown	NcsE3 (AAM78015)	74/83
CB02400_E2	329	Unknown	NcsE2 (AAM78016)	69/76
CB02400_E1	147	HxlR family transcriptional regulator	NcsE1 (AAM78017)	71/84
CB02400_orf79	402	Transcriptional regulator	NcsR7 (AAM78019)	60/70
CB02400_orf80	229	TetR family transcriptional regulator	NcsR3 (AAM78020)	51/67
CB02400_orf81	327	Autoregulator biosynthesis enzyme	NcsC2 (AAM78021)	69/76
CB02400_orf82	199	Butyrolactone receptor	NcsR2 (AAM78022)	67/76
CB02400_orf83	319	Gama-butyrolactone biosynthetic protein	NcsR1 (AAM78023)	59/69
CB02400_orf84	461	Hypothetical protein	SSFG_07727 (EFE72492)	60/67
CB02400_orf85	262	Unknown	NcsORF55 (AAM78024)	73/82
CB02400_orf(+1)	227	Hypothetical protein	SSAG_02705 (EDX22914)	64/73

[a]*orf(-1)* and *orf(+1)* are predicted to represent the upstream and downstream boundaries of the enediyne gene cluster.
[b]Number of amino acids.
[c] [c]Also see Figure 4 and Figure 2C for the organization of the enediyne gene cluster.

Table S4-8. Related to Figures 2, 4. Predicted functions of ORFs in the enediyne biosynthetic gene cluster from *Streptomyces* sp. TSRI0281

gene[a]	aa[b]	putative function[c]	protein homologue	% identity/ % similarity
TSRI0281_orf(-1)	286	Taurine dioxygenase	SSAG_06914 (EDX27123)	82/90
TSRI0281_orf1	422	Integral membrane protein LmrP	SSAG_06912 (EDX27121)	72/82
TSRI0281_orf2	407	Phosphoribosylglycinamide synthetase	M444_37775 (AKL71127)	61/74
TSRI0281_orf3	427	Carboxylase	M444_37780 (AKL71128)	65/75
TSRI0281_orf4	212	Serine acetyltransferase	ADK74_31290 (KOG38831)	73/84
TSRI0281_orf5	317	Cysteine synthase	ADK74_31285 (KOG38830)	74/83
TSRI0281_orf6	413	NikS protein	SSAG_05939 (EDX26128)	71/78
TSRI0281_orf7	590	Amino acid adenylation domain	SpoT2 (ABP55169)	56/68
TSRI0281_orf8	457	Type II condensation domain protein	MdpC5 (ABY66007)	45/57
TSRI0281_orf9	413	Hydroxylase	SpoORF17 (ABP55164)	65/76
TSRI0281_orf10	414	Cytochrome P450 monooxygenase	KedN3 (AFV52151)	76/83

TSRI0281_orf11	449	FAD-dependent monooxygenase	KedD2 (AFV52150)	67/77
TSRI0281_J	143	Unknown	KedJ (AFV52149)	59/74
TSRI0281_orf13	393	Oxidoreductase	MdpD2 (ABY66021)	71/83
TSRI0281_orf14	511	Hydroxylase	SpoT3 (ABP55157)	79/91
TSRI0281_orf15	225	Acetyltransferase	B005_4669 (AFR10201)	48/56
TSRI0281_orf16	343	NAD-dependent epimerase	ADL35_15005 (KPC85895)	83/90
TSRI0281_orf17	444	Hypothetical protein	ADL35_15000 (KPC85503)	77/84
TSRI0281_orf18	157	Hypothetical protein	ADL35_14995 (KPC85502)	53/67
TSRI0281_orf19	311	Hypothetical protein	Caci_8969 (ACU77782)	42/52
TSRI0281_orf20	478	DNA polymerase subunit beta	SCLAV_p0482 (EFG03972)	40/51
TSRI0281_orf21	259	Hypothetical protein	WP_054811771	54/64
TSRI0281_orf22	372	Radical SAM domain protein	Caci_8971 (ACU77784)	73/86
TSRI0281_orf23	365	Radical SAM domain protein	Caci_8972 (ACU77785)	73/81
TSRI0281_orf24	431	Glutamate--tRNA ligase	AQ490_06330 (KRV47635)	73/81
TSRI0281_orf25	556	Hypothetical protein	AQJ66_32270 (KUN77897)	68/76
TSRI0281_orf26	332	Methyltransferase	M444_37930 (AKL71155)	77/88
TSRI0281_orf27	192	Alkylhydroperoxidase	SgcO (WP_010056303)	76/83
TSRI0281_orf28	216	Hypothetical protein	SgcP (AAL06688)	54/66
TSRI0281_orf29	382	Regulator	SgcR (AAL06689)	84/91
TSRI0281_orf30	324	Oxidase	SgcQ (AAL06690)	64/76
TSRI0281_orf31	256	4'-Phosphopantetheinyl transferase	APS67_04678 (KTF43851)	55/62
TSRI0281_orf32	126	Hypothetical protein	SSAG_06910 (EDX27119)	59/79
TSRI0281_orf33	392	S-adenosylmethionine synthetase	AW27_24875 (EYU67025)	87/92
TSRI0281_M	348	Unknown	SgcM (AAL06686)	62/68
TSRI0281_orf35	328	Prephenate dehydrogenase	TyrC (CNE74985)	50/61
TSRI0281_E11	267	Unknown	NcsE11 (AAM78004)	82/90
TSRI0281_E10	156	Type II thioesterase	SgcE10 (AAL06692)	86/92
TSRI0281_E9	552	Oxidoreductase	SgcE9 (AAL06693)	93/97
TSRI0281_E8	196	Unknown	SgcE8 (AAL06694)	82/90
TSRI0281_orf40	370	Regulatory protein	SgcR1 (AAL06695)	73/82
TSRI0281_orf41	258	AraC family transcriptional regulator	SgcR2 (AAL06696)	72/81
TSRI0281_E7	449	Cytochrome P450	SgcE7 (AAL06697)	85/91
TSRI0281_E6	182	Flavin reductase	SgcE6 (AAL06698)	69/76
TSRI0281_E10	154	Type II thioesterase	SgcE10 (AAL06692)	51/66
TSRI0281_E	1937	Enediyne polyketide synthase	SgcE (AAL06699)	82/88
TSRI0281_E5	313	Unknown	SgcE5 (AAL06700)	81/85
TSRI0281_E4	642	Unknown	SgcE4 (AAL06701)	86/93
TSRI0281_E3	328	Unknown	SgcE3 (AAL06702)	83/90
TSRI0281_E2	327	Unknown	SgcE2 (AAL06703)	66/75
TSRI0281_E1	147	HxlR family transcriptional regulator	SgcE1 (AAL06704)	80/89
TSRI0281_orf51	187	Unknown	SgcS (AAL06705)	83/89
TSRI0281_orf52	143	Unknown	SgcT (AAL06706)	66/74
TSRI0281_orf53	413	Transcriptional regulator	SgcR3 (AAL06707)	75/84
TSRI0281_orf54	201	TetR family transcriptional regulator	NcsR3 (AAM78020)	44/61
TSRI0281_orf55	254	Short chain dehydrogenase	KedU16 (AFV52172)	35/48
TSRI0281_orf56	224	Butyrolactone receptor	NcsR2 (AAM78022)	41/61
TSRI0281_orf57	305	Gama-butyrolactone biosynthetic protein	NcsR1 (AAM78023)	39/54
TSRI0281_orf(+1)	557	DNA primase (plasmid)	SgcORF60 (AAL06713)	84/90

[a]*orf(-1)* and *orf(+1)* are predicted to represent the upstream and downstream boundaries of the enediyne gene cluster.
[b]Number of amino acids.
[c]Also see Figure 4 and Figure 2C for the organization of the enediyne gene cluster.

Table S4-9. Related to Figures 2, 4. Predicted functions of ORFs in the enediyne biosynthetic gene cluster (partial) from *Streptomyces* sp. CB01580

gene[a]	aa[b]	putative function[c]	protein homologue	% identity/ % similarity
CB01580_orf(-1)	355	Hypothetical protein	SPW_3202 (EHM28417)	78/84
CB01580_orf1	253	Hypothetical protein	Cyaorf27 (AGO97171)	55/68
CB01580_orf2	116	Alkylhydroperoxidase	SgcO (WP_010056303)	88/92
CB01580_orf3	306	Oxidoreductase	SgcN (AAL06687)	85/89
CB01580_M	345	Alkylhydroperoxidase	SgcM (AAL06686)	79/84
CB01580_orf5	104	Sphingomyelin synthase 2	SACT1_0707 (EGE40096)	76/84
CB01580_E7	449	Cytochrome P450	SgcE7 (AAL06697)	84/90
CB01580_E6	174	Flavin reductase	SgcE6 (AAL06698)	72/80
CB01580_E	1938	Enediyne polyketide synthase	SgcE (AAL06699)	81/87
CB01580_E5	380	Unknown	SgcE5 (AAL06700)	80/85
CB01580_E4	642	Unknown	SgcE4 (AAL06701)	82/91
CB01580_E3	322	Unknown	sgcE3 (AAL06702)	82/89
CB01580_E2	327	Unknown	SgcE2 (AAL06703)	84/89
CB01580_orf13	319	Unknown	NcsORF56 (AAM78025)	66/78
CB01580_E10	159	Type II thioesterase	SgcE10 (AAL06692)	68/81
CB01580_J	142	Unknown	SgcJ (pdb|4I4K|A)	56/72
CB01580_orf16	259	AraC family transcriptional regulator	SgcR2 (AAL06696)	49/61
CB01580_E8	189	Unknown	SgcE8 (AAL06694)	59/71
CB01580_E9	551	Oxidoreductase	MdpE9 (ABY65999)	80/87
CB01580_orf19	415	*C*-3'-Methyltransferase	SgcA3 (AAL0661)	38/51
CB01580_orf20	326	dTDP-D-glucose synthase	SgcA1 (AAL06657)	56/70
CB01580_orf21	415	FAD-binding monooxygenase	MdpD2 (ABY66021)	69/79
CB01580_orf22	539	MFS transporter	SgcB (AAL06672)	53/68
CB01580_orf23	1111	Regulatory protein	KedR1 (AFV52162)	50/61
CB01580_orf24	390	Sugar aminotransferase	KedS7 (AFV52158)	56/68
CB01580_orf25	445	Glycosyl transferase	SgcA6 (AAL06670)	44/60
CB01580_orf26	771	Subtilisin-like serine protease	SCLAV_p1364 (EFG04848)	69/76
CB01580_orf27	461	FAD-binding monooxygenase	MdpL (ABY66029)	73/79
CB01580_orf28	238	*N*-methyltransferase	KedS9 (AFV52160)	48/65
CB01580_F	397	Epoxide hydrolase	NcsF2 (AAM78002)	64/73
CB01580_E11	267	Unknown	NcsE11 (AAM78004)	64/77
CB01580_orf31	114	Unknown	SgcORF56 (AAL06709)	56/63

[a]*orf(-1)* is predicted to represent the upstream boundary of the enediyne gene cluster.
[b]Number of amino acids.
[c]Also see Figure 4 and Figure 2C for the organization of the enediyne gene cluster.

Table S4-10. Related to Figures 2, 4. Predicted functions of ORFs in the enediyne biosynthetic gene cluster (partial) from *Streptomyces* sp. CB00316

gene	aa[a]	putative function[b]	protein homologue	% identity/ % similarity
CB00316_E7	449	Cytochrome P450	NcsE7 (AAM78009)	71/79
CB00316_E6	182	Flavin reductase	SgcE6 (AAL06698)	70/77
CB00316_E	1957	Enediyne polyketide synthase	SgcE (AAL06699)	80/86
CB00316_E5	358	Unknown	SgcE5 (AAL06700)	81/85
CB00316_E4	642	Unknown	SgcE4 (AAL06701)	90/95
CB00316_E3	320	Unknown	SgcE3 (AAL06702)	82/90
CB00316_E2	332	Unknown	SgcE2 (AAL06703)	78/86
CB00316_E10	155	Type II thioesterase	SgcE10 (AAL06692)	66/80

CB00316_orf9	209	Alkylhydroperoxidase	SgcORF-6 (AAL06649)	44/54
CB00316_orf10	630	Hypothetical protein	O3I_015930 (AFU01147)	48/63
CB00316_orf11	408	PEP-utilizing protein	SACT1_0691 (EGE40080)	86/91
CB00316_E9	551	Oxidoreductase	MdpE9 (ABY65999)	79/87
CB00316_E8	186	Unknown	NcsE8 (AAM78006)	60/71
CB00316_orf14	259	AraC family transcriptional regulator	MdpR2 (ABY65996)	51/66
CB00316_E11	267	Unknown	MdpE11 (ABY66001)	55/69
CB00316_J	135	Unknown	MdpJ (ABY66022)	57/78
CB00316_orf17	148	Endoribonuclease L-PSP	SCATT_57860 (AEW98157)	89/91
CB00316_orf18	455	Hypothetical protein	SBI_03716 (ADI06837)	85/88
CB00316_orf19	73	Predicted protein	SSNG_07258 (EFL20006)	82/89
CB00316_orf20	102	Hypothetical protein	SSNG_07259 (EFL20007)	74/84
CB00316_orf21	163	Transposase, partial	WP_040702653	68/75
CB00316_orf22	396	Acyl-CoA dehydrogenase	SgcL (AAL06685)	60/72
CB00316_orf23	338	Zn-dependent alcohol dehydrogenase	MdpC8 (ABY66028)	67/80
CB00316_orf24	398	Isovaleryltransferase	CynA4 (AGO97170)	41/60
CB00316_orf25	521	MFS transporter	Spoorf15 (ABP55162)	51/66
CB00316_orf26	242	Unknown	Spoorf22 (ABP55176)	39/57
CB00316_orf27	495	FAD-dependent monooxygenase	MdpL (ABY66029)	55/71
CB00316_orf28	84	Type II PCP domain protein	MdpC2 (ABY66003)	51/65
CB00316_orf29	293	Phytanoyl-CoA dioxygenase	STTU_4295 (EGJ77084)	89/93
CB00316_orf30	500	FAD-linked oxidase	SpoT4 (ABP55175)	26/38
CB00316_orf31	422	RNA polymerase subunit sigma-24	Spoorf11 (ABP55158)	53/66
CB00316_orf32	470	Oxidoreductase	SpoT4 (ABP55175)	29/42
CB00316_orf33	257	Short-chain dehydrogenase	KedU16 (AFV52172)	34/43
CB00316_orf34	419	Acyl-CoA dehydrogenase	Spoorf17 (ABP55164)	55/68
CB00316_orf35	422	Type II condensation domain protein	MdpC5 (ABY66007)	48/62
CB00316_orf36	280	Methyltransferase type 11	MitM (AAD28459)	33/50
CB00316_orf37	125	Putative DGPFAETKE family protein	Spoorf12 (ABP55159)	42/61
CB00316_orf38	410	Cytochrome P450 hydroxylase	NcsB3 (AAM77997)	44/60
CB00316_orf39	1052	Type II beta-Tyr adenylation domain protein	MdpC1 (ABY66004)	40/50
CB00316_orf40	539	Tyrosine aminomutase	MdpC4 (ABY66005)	73/82
CB00316_orf41	509	Monooxygenase	MdpC (ABY66026)	81/90
CB00316_orf42	119	Hypothetical protein	Spoorf16 (ABP55163)	47/72
CB00316_orf43	464	FAD-linked oxidase	SpoT4 (ABP55175)	34/50
CB00316_orf44	709	Molybdopterin-binding oxidoreductase	Sros_0816 (ACZ83830)	68/80
CB00316_orf45	526	Type 11 methyltransferase	SpoT6 (ABP55177)	48/60
CB00316_orf46	453	Glycerol phosphate ABC transporter	SgcB1 (AAL06653)	49/63

[a]Number of amino acids.
[b]Also see Figure 4 and Figure 2C for the organization of the enediyne gene cluster.

Table S4-11. Related to Figures 2, 4. Predicted functions of ORFs in the enediyne biosynthetic gene cluster (partial) from *Streptomyces* sp. CB02058

gene[a]	aa[b]	putative function[c]	protein homologue	% identity/ % similarity
CB02058_orf1	331	NDP-hexose reductase	CalS12 (AAM70349)	65/76
CB02058_orf2	421	*O*-Acyltransferase	CynA4(AGO97170)	32/49
CB02058_orf3	413	Alkylhalidase	CalO3 (AAM70353)	52/69
CB02058_orf4	450	C-domain type II peptide synthetase	SgcC5 (AAL06678)	40/54
CB02058_orf5	375	4-Hydroxyphenylpyruvate dioxygenase	SpoT1 (ABP55171)	57/68
CB02058_orf6	70	MbtH-like protein	H480_12332 (EOD68243)	62/70

CB02058_orf7	581	Thioester reductase	SpoT2 (ABP55169)	55/67
CB02058_orf8	474	FAD linked oxidase	MCAG_02308 (EEP71981)	68/76
CB02058_orf9	222	Glutamate racemase	H480_39545 (EOD62955)	62/70
CB02058_orf10	427	GntR family transcriptional regulator	H480_39540 (EOD62954)	69/81
CB02058_orf11	271	Methyltransferase	SIRAN144 (CDR01144)	47/60
CB02058_orf12	216	Hypothetical protein	AMETH_5624 (AIJ25716)	44/58
CB02058_orf13	261	2-Hydroxycyclohexanecarboxyl-CoA dehydrogenase	DV20_28550 (KDN18898)	64/74
CB02058_orf14	463	Sodium/hydrogen exchanger	SgcB3 (AAL06655)	50/65
CB02058_orf15	359	Glucose-1-phosphate thymidylyltransferase	SgcA1 (AAL06657)	57/73
CB02058_orf16	307	Oxidoreductase	SgcN (AAL06687)	69/78
CB02058_orf17	410	Regulator	SgcR (AAL06689)	72/84
CB02058_orf18	324	Putative oxidase	SgcQ (AAL06690)	59/72
CB02058_M	351	Unknown	SgcM (AAL06686)	61/69
CB02058_orf20	357	Prephenate dehydrogenase	SFRA_22575 (KDS85806)	38/49
CB02058_E11	267	Unknown	NcsE11 (AAM78004)	74/83
CB02058_E10	157	Type II thioesterase	NcsE10 (AAM78011)	85/90
CB02058_E9	552	Oxidoreductase	NcsE9 (AAM78005)	85/91
CB02058_E8	196	Unknown	NcsE8 (AAM78006)	73/84
CB02058_orf25	346	Regulatory protein	SgcR1 (AAL06695)	67/78
CB02058_orf26	257	AraC family transcriptional regulator	SgcR2 (AAL06696)	64/77
CB02058_E7	449	Cytochrome P450	SgcE7 (AAL06697)	88/92
CB02058_E6	182	Flavin reductase	SgcE6 (AAL06698)	71/75
CB02058_E	1941	Enediyne polyketide synthase	SgcE (AAL06699)	86/90
CB02058_E5	374	Unknown	SgcE5 (AAL06700)	87/90
CB02058_E4	642	Unknown	SgcE4 (AAL06701)	92/95
CB02058_E3	328	Unknown	SgcE3 (AAL06702)	89/94
CB02058_E2	317	Unknown	SgcE2 (AAL06703)	85/90
CB02058_E1	147	HxlR family transcriptional regulator	SgcE1 (AAL06704)	81/89
CB02058_orf35	187	Unknown	SgcS (AAL06705)	82/90
CB02058_orf36	141	Unknown	SgcT (AAL06706)	61/70
CB02058_orf37	396	TylR regulatory protein-like protein	SgcR3 (AAL06707)	74/82
CB02058_orf38	200	TetR family transcriptional regulator	NcsR3 (AAM78020)	43/60
CB02058_orf39	256	Short-chain dehydrogenase	KedU16 (AFV51172)	34/46
CB02058_orf40	224	Butyrolactone receptor	NcsR2 (AAM78022)	43/63
CB02058_orf41	311	Gama-butyrolactone biosynthetic protein	NcsR1 (AAM78023)	38/52
CB02058_orf(+1)	466	Primase/helicase-like protein	SgcORF60 (AAL06713)	80/86

[a]*orf(+1)* is predicted to represent the downstream boundary of the enediyne gene cluster.
[b]Number of amino acids.
[c]Also see Figure 4 and Figure 2C for the organization of the enediyne gene cluster.

Table S4-12. Related to Figures 2, 4. Predicted functions of ORFs in the enediyne biosynthetic gene cluster from *Streptomyces* sp. CB02414

gene[a]	aa[b]	putative function[c]	protein homologue	% identity/ % similarity
CB02414_orf(-1)	267	Phytanoyl-CoA dioxygenase	T261_7676 (AJT69273)	60/73
CB02414_orf1	326	dTDP-glucose 4,6-dehydratase	CalS3 (AAM94770)	71/79
CB02414_orf2	205	dTDP-4-dehydrorhamnose 3,5-epimerase	SgcA2 (AAL06668)	42/54
CB02414_orf3	288	Sugar *O*-methyltransferase	NanM (AAP42862)	47/65
CB02414_orf4	295	Glucose-1-phosphate thymidylyltransferase	CynA1 (AGO97177)	57/75
CB02414_M	355	Unknown	SgcM (AAL06686)	47/55
CB02414_orf6	459	Hydroxylase	NcsORF34 (AAM78003)	49/64

CB02414_orf7	492	Type II beta-Tyr adenylation domain protein	MdpC1 (ABY66004)	52/61
CB02414_orf8	500	Monooxygenase	MdpC (ABY66026)	81/89
CB02414_orf9	480	Oxidoreductase-like protein	Aur2I (AAK61713)	47/61
CB02414_orf10	709	Molybdopterin-binding oxidoreductase	STTU_4313 (EGJ77102)	66/76
CB02414_orf11	537	MIO-dependent tyrosine 2,3-aminomutase	SgcC4 (AAL06680)	75/83
CB02414_orf12	402	Sugar aminotransferase	MdpA5 (ABY66023)	56/68
CB02414_orf13	425	*C*-methyltransferase	KedS4 (AFV52186)	59/73
CB02414_orf14	448	NDP-hexose 2,3-dehydratase	CalS14 (AAM70359)	48/60
CB02414_orf15	293	NDP-hexose-3-ketoreductase	KedS3 (AFV52211)	30/37
CB02414_orf16	557	Major facilitator superfamily transporter	SgcB (AAL06672)	51/67
CB02414_orf17	492	Methyltransferase domain protein	I548_5298 (EUA27189)	41/53
CB02414_orf18	1036	Amino acid adenylation protein	Sfla_3512 (ADW04932)	40/54
CB02414_orf19	425	Glycosyltransferase	KedS10 (AFV52161)	50/65
CB02414_orf20	411	Cytochrome P450 monooxygenase	KedN3 (AFV52151)	43/62
CB02414_orf21	277	Dioxygenase	KirHVI (AGS73543)	57/71
CB02414_orf22	89	Type II PCP domain protein	MdpC2 (ABY66003)	51/64
CB02414_orf23	393	Oxidoreductase	MdpD2 (ABY66021)	56/71
CB02414_orf24	463	Glycerol phosphate ABC transporter	SgcB1 (AAL06653)	45/59
CB02414_J	142	Unknown	MdpJ (ABY66022)	61/78
CB02414_orf26	449	C-domain type II peptide synthetase	SgcC5 (AAL06678)	45/60
CB02414_orf27	414	Acyl-CoA dehydrogenase	Spoorf17 (ABP55164)	59/71
CB02414_orf28	194	Prephenate dehydrogenase	SSFG_07703 (EFE72468)	52/64
CB02414_E11	267	Unknown	NcsE11 (AAM78004)	71/77
CB02414_E10	152	Type II thioesterase	NcsE10 (AAM78011)	83/88
CB02414_E9	554	Oxidoreductase	NcsE9 (AAM78005)	84/90
CB02414_E8	190	Unknown	NcsE8 (AAM78006)	69/80
CB02414_orf33	338	Transcriptional regulator	NcsR6 (AAM78007)	49/68
CB02414_orf34	256	AraC family transcriptional regulator	NcsR5 (AAM78008)	57/70
CB02414_E7	448	Cytochrome P450	NcsE7 (AAM78009)	70/80
CB02414_E6	183	Flavin reductase	NcsE6 (AAM78010)	67/72
CB02414_orf37	150	Type II thioesterase	NcsE10 (AAM78011)	33/49
CB02414_E	1962	Enediyne polyketide synthase	SgcE (AAL06699)	69/78
CB02414_E5	342	Unknown	SgcE5 (AAL06700)	80/87
CB02414_E4	654	Unknown	SgcE4 (AAL06701)	71/81
CB02414_E3	332	Unknown	SgcE3 (AAL06702)	69/80
CB02414_E2	326	Unknown	SgcE2 (AAL06703)	68/79
CB02414_E1	147	Unknown	SgcE1 (AAL06704)	69/80
CB02414_orf44	391	Transcriptional regulator	NcsR7 (AAM78019)	51/66
CB02414_orf45	326	Unknown	NcsORF56 (AAM78025)	66/79
CB02414_orf46	243	TetR family transcriptional regulator	NcsR3 (AAM78020)	41/57
CB02414_orf47	248	Short-chain dehydrogenase	KedU16 (AFV52172)	31/44
CB02414_orf48	227	Phosphatase	Lct11 (ABX71094)	53/64
CB02414_orf49	317	Gama-butyrolactone biosynthetic protein	NcsR1 (AAM78023)	54/68
CB02414_orf50	210	Butyrolactone receptor	NcsR2 (AAM78022)	46/65
CB02414_orf(+1)	120	Deaminase	SFUL_5634 (AGK80521)	75/87

[a]*orf(-1)* and *orf(+1)* are predicted to represent the upstream and downstream boundaries of the enediyne gene cluster.
[b]Number of amino acids.
[c]Also see Figure 4 and Figure 2C for the organization of the enediyne gene cluster.

Table S4-13. Related to Figures 2, 4. Predicted functions of ORFs in the enediyne biosynthetic gene cluster from *Streptomyces* sp. CB02460

gene[a]	aa[b]	putative function[c]	protein homologue	% identity/ % similarity
CB02460_orf(-1)	286	Phytanoyl-CoA dioxygenase	Z951_28675 (EXU64826)	47/59
CB02460_M	345	Unknown	SgcM (AAL06686)	53/60
CB02460_orf2	395	Acyl-CoA dehydrogenase	KedU41 (AFV52203)	30/42
CB02460_orf3	325	Dehydrogenase	SBI_07609 (ADI10729)	52/65
CB02460_orf4	461	FAD-binding monooxygenase	KedD2 (AFV52150)	57/67
CB02460_F	396	Epoxide hydrolase	NcsF2 (AAM78002)	62/72
CB02460_orf6	387	Oxidoreductase	SgcL (AAL06685)	56/70
CB02460_orf7	245	Alpha/beta hydrolase	KedU43 (AFV52205)	42/50
CB02460_orf8	183	Hypothetical protein	SgcO (WP_010056303)	71/78
CB02460_orf9	456	Type II condensation domain protein	MdpC5 (ABY66007)	39/49
CB02460_orf10	183	Hypothetical protein	Sros_6984 (ACZ89684)	48/59
CB02460_E11	267	Unknown	NcsE11 (AAM78004)	67/76
CB02460_E10	147	Type II thioesterase	NcsE10 (AAM78011)	87/95
CB02460_E9	557	Oxidoreductase	NcsE9 (AAM78005)	81/89
CB02460_E8	190	Unknown	NcsE8 (AAM78006)	67/80
CB02460_orf15	332	Transcriptional regulator	NcsR6 (AAM78007)	51/67
CB02460_orf16	257	AraC family transcriptional regulator	NcsR5 (AAM78008)	56/70
CB02460_E7	448	Cytochrome P450	NcsE7 (AAM78009)	68/77
CB02460_E6	180	Flavin reductase	NcsE6 (AAM78010)	63/76
CB02460_orf19	121	Type II thioesterase	NcsE10 (AAM78011)	34/49
CB02460_E	1956	Enediyne polyketide synthase	NcsE (AAM78012)	68/76
CB02460_E5	306	Unknown	NcsE5 (AAM78013)	70/79
CB02460_E4	641	Unknown	NcsE4 (AAM78014)	68/78
CB02460_E3	312	Unknown	NcsE3 (AAM78015)	62/74
CB02460_E2	329	Unknown	NcsE2 (AAM78016)	66/74
CB02460_E1	146	Unknown	NcsE1 (AAM78017)	66/80
CB02460_orf26	208	Butyrolactone receptor	NcsR2 (AAM78022)	35/49
CB02460_orf27	391	Transcriptional regulator	NcsR7 (AAM78019)	50/64
CB02460_orf28	195	TetR family transcriptional regulator	NcsR3 (AAM78020)	44/65
CB02460_orf29	317	Autoregulator biosynthesis protein	NcsC2 (AAM78021)	56/69
CB02460_orf30	194	Butyrolactone receptor	NcsR2 (AAM78022)	54/68
CB02460_orf31	334	Gama-butyrolactone biosynthetic protein	NcsR1 (AAM78023)	51/64
CB02460_orf32	449	Unknown	WP_027769280	58/67
CB02460_orf33	128	Hypothetical protein	Cynorf21 (AGO97165)	42/56
CB02460_F2	385	Epoxide hydrolase	NcsF2 (AAM78002)	61/71
CB02460_orf35	448	FAD-dependent monooxygenase	KedD2 (AFV52150)	56/71
CB02460_orf36	88	Sulfur transfer protein	CalU18 (AAM70358)	40/55
CB02460_orf37	297	Sulphatase-modifying factor protein	CalU17 (AAM70357)	62/73
CB02460_orf38	460	Type II condensation domain protein	MdpC5 (ABY66007)	45/60
CB02460_orf39	446	Glycosyl transferase	SgcA6 (AAL06670)	40/54
CB02460_orf40	522	Transmembrane efflux protein	MdpR3 (ABY66009)	45/65
CB02460_orf41	1427	Alpha-mannosidase	SCATT_54970 (AEW97868)	50/60
CB02460_orf42	187	dTDP-4-dehydrorhamnose 3,5-epimerase	SgcA2 (AAL06668)	66/77
CB02460_orf43	226	Pyridoxamine 5'-phosphate oxidase	SSFG_04083 (EFE68840)	60/75
CB02460_orf44	406	Glycosyl transferase family 1	CalG4 (AAM70365)	25/36
CB02460_orf45	394	Glycosyl transferase group 1	Rpdx1_2124 (ADU43722)	37/52
CB02460_orf46	269	Hypothetic protein	WP_030062606	38/48
CB02460_orf47	687	Hypothetical protein	MPTA5024_34085 (ETK31637)	47/58
CB02460_orf48	356	dTDP-1-glucose synthase	MdpA1 (ABY66030)	58/74
CB02460_orf49	455	UDP-glucose 6-dehydrogenase	MdpA2 (ABY66024)	67/77
CB02460_orf50	331	Glucuronic acid decarboxylase	MdpA3 (ABY66025)	71/79
CB02460_orf51	121	Hypothetical protein	SVEN_3959 (CCA57245)	59/71

CB02460_orf(+1)	218	Maleylpyruvate isomerase	DC60_30390 (KDR63913)	76/82

[a]*orf(-1)* and *orf(+1)* are predicted to represent the upstream and downstream boundaries of the enediyne gene cluster.
[b]Number of amino acids.
[c]Also see Figure 4 and Figure 2C for the organization of the enediyne gene cluster.

Table S4-14. Related to Figures 2, 4. Predicted functions of ORFs in the enediyne biosynthetic gene cluster from *Streptomyces* sp. TSRI0395

gene[a]	aa[b]	putative function[c,d]	protein homologue	% identity/ % similarity
TSRI0395_orf(-1)	173	Hypothetical protein	pFRL5_45c (AHE39708)	85/89
TSRI0395_orf1	416	Hypothetical protein	SPW_3227 (EHM28442)	93/96
TSRI0395_orf2	192	Transporter	SPW_3226 (EHM28441)	96/97
TSRI0395_orf3	95	Copper-sensing transcriptional repressor	SPW_3225 (EHM28440)	100/100
TSRI0395_orf4	244	Protein of unknown function DUF81	SPW_3224 (EHM28439)	97/97
TSRI0395_orf5	121	Sulfurtransferase	SPW_3223 (EHM28438)	93/94
TSRI0395_orf6	454	Beta-lactamase	SPW_3222 (EHM28437)	97/98
TSRI0395_orf7	584	Glucoamylase	SPW_3221 (EHM28436)	96/97
TSRI0395_orf8	249	*N*-Methyltransferase	SPW_3220 (EHM28435)	95/97
TSRI0395_orf9	417	Aminotransferase class I and II	SPW_3219 (EHM28434)	99/99
TSRI0395_orf10	1056	Singaling protein	SPW_3218 (EHM28433)	95/96
TSRI0395_orf11	547	PA14 domain-containing protein	SPW_3217 (EHM28432)	93/95
TSRI0395_orf12	335	NAD-dependent epimerase/dehydratase	MdpA3 (ABY66025)	37/49
TSRI0395_orf13	439	UDP-glucose 6-dehydrogenase	MdpA2 (ABY66024)	34/45
TSRI0395_orf14	351	UDP-glucose 4-epimerase	NcsC1 (AAM77990)	29/44
TSRI0395_orf15	608	*N*-Acetylglucosaminyltransferase	SPW_3213 (EHM28428)	98/98
TSRI0395_orf16	243	TetR family transcriptional regulator	SPW_3212 (EHM28427)	94/96
TSRI0395_orf17	408	Hypothetical protein	SPW_3211 (EHM28426)	95/97
TSRI0395_orf18	216	Hypothetical protein	Sfla_4470 (ADW05875)	74/79
TSRI0395_orf19	420	Rieske (2Fe-2S) iron-sulfur domain protein	SPW_3210 (EHM28425)	96/97
TSRI0395_orf20	252	AraC family transcriptional regulator	MdpR2 (ABY65996)	51/62
TSRI0395_E6	198	Flavin reductase	SgcE6 (AAL06698)	52/60
TSRI0395_E10	150	Type II thioesterase	SgcE10 (AAL06692)	73/81
TSRI0395_E	1952	Enediyne polyketide synthase	SgcE (AAL06699)	67/76
TSRI0395_E5	323	Unknown	SgcE5 (AAL06700)	68/81
TSRI0395_E4	635	Unknown	SgcE4 (AAL06701)	64/76
TSRI0395_E3	311	Unknown	SgcE3 (AAL06702)	56/65
TSRI0395_orf27	250	Hypothetical protein	Cynorf27 (AGO97171)	98/99
TSRI0395_orf28	371	Hypothetical protein	SPW_3202 (EHM28417)	97/98
TSRI0395_orf29	265	Methyltransferase	PsmC (AHL44341)	38/51
TSRI0395_orf30	244	Short chain dehydrogenase	KedU16 (AFV52172)	34/47
TSRI0395_orf31	156	Activator of HSP90 ATPase	SPW_3199 (EHM28414)	99/98
TSRI0395_orf32	170	Hypothetical protein	CalU16 (AAM70339)	35/46
TSRI0395_orf33	401	Major facilitator transporter	SPW_3197 (EHM28412)	99/99
TSRI0395_orf34	148	Hypothetical protein	SPW_3196 (EHM28411)	99/100
TSRI0395_orf35	214	HxlR family transcriptional regulator	DynU8 (ACB47051)	46/58
TSRI0395_orf36	390	PBS lyase HEAT-like repeat protein	DynORF19 (ACB047059)	29/45
TSRI0395_orf37	447	Putative regulator	DynR3 (ACB47054)	46/60
TSRI0395_orf38	246	Hypothetical protein	SPW_3192 (EHM28407)	92/94
TSRI0395_orf39	192	Hypothetical protein	SgcORF58 (AAL06711)	70/77
TSRI0395_orf40	187	Unknown	CalU21 (AAM70363)	65/78

TSRI0395_orf41	168	Unknown	SgcT (AAL06706)	81/85
TSRI0395_orf(+1)	137	Pyridoxamine 5'-phosphate oxidase	DF18_03160 (KEF22332)	74/85

[a]*orf(-1)* and *orf(+1)* are predicted to represent the upstream and downstream boundaries of the enediyne gene cluster.
[b]Number of amino acids.
[c]Also see Figure 4 and Figure 2C for the organization of the enediyne gene cluster.
[d]Selected strains from the same clade (Figure 2A, panel B) that have been sequenced, yielding highly homologous gene clusters (also see Tables S4-15, S4-16, S4-17). Only the cluster from CB00072 was shown in Figure 2C, but all four homologous gene clusters are included in the enediyne GNN (Figure 4).

Table S4-15. Related to Figures 2, 4. Predicted functions of ORFs in the enediyne biosynthetic gene cluster from *Streptomyces* sp. TSRI0261

gene[a]	aa[b]	putative function[c,d]	protein homologue	% identity/ % similarity
TSRI0261_orf(-1)	471	Secreted protein	P376_1243 (ESU50765)	87/90
TSRI0261_orf1	175	Hypothetical protein	F750_0394 (AGJ52905)	80/84
TSRI0261_orf2	417	Hypothetical protein	SFUL_128 (AGK75113)	74/80
TSRI0261_orf3	192	Transporter	SPW_3226 (EHM28441)	96/98
TSRI0261_orf4	95	Copper-sensing transcriptional repressor	SPW_3225 (EHM28440)	99/100
TSRI0261_orf5	244	Hypothetical protein	SPW_3224 (EHM28439)	97/98
TSRI0261_orf6	121	Sulfurtransferase	SPW_3223 (EHM28438)	93/94
TSRI0261_orf7	454	Beta-lactamase	SPW_3222 (EHM28437)	98/98
TSRI0261_orf8	217	Hypothetical protein	Sfla_4470 (ADW05875)	74/79
TSRI0261_orf9	409	Rieske (2Fe-2S) iron-sulfur domain protein	SPW_3210 (EHM28425)	94/96
TSRI0261_orf10	213	AraC family transcriptional regulator	MdpR2 (ABY65996)	48/57
TSRI0261_E6	198	Flavin reductase	MdpE6 (AAQ17112)	58/69
TSRI0261_E10	150	Type II thioesterase	SgcE10 (AAL06692)	74/83
TSRI0261_E	1952	Enediyne polyketide synthase	SgcE (AAL06699)	67/76
TSRI0261_E5	323	Unknown	SgcE5 (AAL06700)	69/81
TSRI0261_E4	635	Unknown	SgcE4 (AAL06701)	64/76
TSRI0261_E3	311	Unknown	SgcE3 (AAL06702)	56/65
TSRI0261_orf17	250	Hypothetical protein	Cynorf27 (AGO97171)	55/68
TSRI0261_orf18	371	Hypothetical protein	SPW_3202 (EHM28417)	99/99
TSRI0261_orf19	269	Methyltransferase	SPW_3201 (EHM28416)	97/98
TSRI0261_orf20	246	Short chain dehydrogenase	KedU16 (AFV52172)	35/48
TSRI0261_orf21	156	Activator of HSP90 ATPase	SPW_3199 (EHM28414)	99/98
TSRI0261_orf22	169	Hypothetical protein	CalU16 (AAM70339)	36/48
TSRI0261_orf23	401	Major facilitator transporter	SPW_3197 (EHM28412)	99/9
TSRI0261_orf24	148	Hypothetical protein	SCLAV_p1238 (EFG04724)	68/80
TSRI0261_orf25	214	Unknown	DynU8 (ACB47051)	46/58
TSRI0261_orf26	398	PBS lyase HEAT-like repeat protein	DynORF16 (ACB47059)	29/45
TSRI0261_orf27	436	Unknown	DynR3 (ACB47054)	46/60
TSRI0261_orf28	244	Hypothetical protein	SPW_3192 (EHM28407)	92/95
TSRI0261_orf29	186	Hypothetical protein	SgcORF58 (AAL06711)	69/76
TSRI0261_orf30	187	Unknown	CalU21 (AAM70363)	64/78
TSRI0261_orf31	168	Unknown	SgcT (AAL06706)	79/83
TSRI0261_orf32	183	PadR family transcriptional regulator	SSDG_07573 (EFH32306)	94/95
TSRI0261_orf33	423	Transporter	Sli4 (ABX00627)	84/90
TSRI0261_orf34	221	NADH dehydrogenase	M271_01215 (AGP51880)	90/95
TSRI0261_orf35	494	Oxidoreductase	SPW_3177 (EHM28392)	98/99
TSRI0261_orf36	132	Thioredoxin	SGR_690 (BAG17519)	99/100
TSRI0261_orf37	91	Hypothetical protein	SSIG_06838 (EWS96053)	99/98
TSRI0261_orf38	340	LacI family transcriptional regulator	ABB07_03680 (AKJ09154)	69/76

TSRI0261_orf(+1)	155	RNA 3'-terminal phosphate cyclase	BF14_0528 (KIX50925)	95/96

[a]*orf(-1)* and *orf(+1)* are predicted to represent the upstream and downstream boundaries of the enediyne gene cluster.
[b]Number of amino acids.
[c]Also see Figure 4 and Figure 2C for the organization of the enediyne gene cluster.
[d]Selected strains from the same clade (Figure 2A, panel B) that have been sequenced, yielding highly homologous gene clusters (also see Tables S4-14, S4-16, S4-17). Only the cluster from CB00072 was shown in Figure 2C, but all four homologous gene clusters are included in the enediyne GNN (Figure 4).

Table S4-16. Related to Figures 2, 4. Predicted functions of ORFs in the enediyne biosynthetic gene cluster from *Streptomyces* sp. CB02115

gene[a]	aa[b]	putative function[c,d]	protein homologue	% identity/ % similarity
CB02115_orf(-1)	171	Hypothetical protein	F750_0394 (AGJ52905)	76/81
CB02115_orf1	411	Hypothetical protein	SPW_3227 (EHM28442)	91/95
CB02115_orf2	192	Transporter	SPW_3226 (EHM28441)	96/97
CB02115_orf3	95	Copper-sensing transcriptional repressor	SPW_3225 (EHM28440)	100/100
CB02115_orf4	247	Protein of unknown function DUF81	SPW_3224 (EHM28439)	98/98
CB02115_orf5	121	Sulfurtransferase	SPW_3223 (EHM28438)	94/95
CB02115_orf6	454	Beta-lactamase	SPW_3222 (EHM28437)	97/97
CB02115_orf7	584	Glucoamylase	SPW_3221 (EHM28436)	97/97
CB02115_orf8	249	*N*-methyltransferase	SPW_3220 (EHM28435)	97/98
CB02115_orf9	417	Aminotransferase class I and II	SPW_3219 (EHM28434)	98/98
CB02115_orf10	1063	Signaling protein	SPW_3218 (EHM28433)	95/96
CB02115_orf11	547	PA14 domain-containing protein	SPW_3217 (EHM28432)	94/97
CB02115_orf12	335	NAD-dependent epimerase/dehydratase	MdpA3 (ABY66025)	38/49
CB02115_orf13	439	UDP-glucose 6-dehydrogenase	CalS8 (AAM70332)	31/43
CB02115_orf14	351	UDP-glucose 4-epimerase	NcsC1 (AAM77990)	99/98
CB02115_orf15	608	*N*-acetylglucosaminyltransferase	SPW_3213 (EHM28428)	99/99
CB02115_orf16	247	TetR family transcriptional regulator	SPW_3212 (EHM28427)	93/95
CB02115_orf17	408	Hypothetical protein	SPW_3211 (EHM28426)	96/97
CB02115_orf18	217	Hypothetical protein	Sfla_4470 (ADW05875)	73/80
CB02115_orf19	409	Rieske (2Fe-2S) iron-sulfur domain protein	SPW_3210 (EHM28425)	97/97
CB02115_orf20	213	AraC family transcriptional regulator	NcsR5 (AAM78008)	47/57
CB02115_E6	194	Flavin reductase	NcsE6 (AAM78010)	58/64
CB02115_E10	150	Type II thioesterase	NcsE10 (AAM78011)	77/84
CB02115_E	1952	Enediyne polyketide synthase	NcsE (AAM78012)	64/74
CB02115_E5	324	Unknown	NcsE5 (AAM78013)	69/81
CB02115_E4	635	Unknown	NcsE4 (AAM78014)	66/76
CB02115_E3	323	Unknown	NcsE3 (AAM78015)	55/65
CB02115_orf27	250	Hypothetical protein	Cynorf27 (AGO97171)	55/68
CB02115_orf28	371	Hypothetical protein	SPW_3202 (EHM28417)	98/98
CB02115_orf29	265	Methyltransferase	SPW_3201 (EHM28416)	98/99
CB02115_orf30	244	Short chain dehydrogenase	KedU16 (AFV52172)	98/98
CB02115_orf31	156	Activator of HSP90 ATPase	SPW_3199 (EHM28414)	99/99
CB02115_orf32	170	Unknown	CalU16 (AAM70339)	34/45
CB02115_orf33	426	Major facilitator transporter	SPW_3197 (EHM28412)	99/99
CB02115_orf34	148	HxlR family transcriptional regulator	CalU8 (AAM94775)	46/58
CB02115_orf35	214	HxlR family transcriptional regulator	CalU8 (AAM94775)	46/62
CB02115_orf36	390	PBS lyase HEAT-like repeat protein	DynORF16 (ACB47059)	29/45
CB02115_orf37	447	Putative regulator	DynR3 (ACB47054)	46/60
CB02115_orf38	244	Hypothetical protein	SPW_3192 (EHM28407)	92/94
CB02115_orf39	192	Hypothetical protein	SgcORF58 (AAL06711)	69/77
CB02115_orf40	187	Unknown	CalU21 (AAM70363)	65/78
CB02115_orf41	168	Unknown	SgcT (AAL06706)	80/83
CB02115_orf(+1)	107	Hypothetical protein	IQ62_05010 (KFG01908)	67/76

[a]*orf(-1)* and *orf(+1*) are predicted to represent the upstream and downstream boundaries of the enediyne gene cluster.
[b]Number of amino acids.
[c]Also see Figure 4 and Figure 2C for the organization of the enediyne gene cluster.
[d]Selected strains from the same clade (Figure 2A, panel B) that have been sequenced, yielding highly homologous gene clusters (also see Tables S4-14, S4-15, S4-17). Only the cluster from CB00072 was shown in Figure 2C, but all four homologous gene clusters are included in the enediyne GNN (Figure 4).

Table S4-17. Related to Figures 2, 4. Predicted functions of ORFs in the enediyne biosynthetic gene cluster from *Streptomyces* sp. CB00072

gene[a]	aa[b]	putative function[c,d]	protein homologue	% identity/ % similarity
CB00072_orf(-1)	124	ArsR family transcriptional regulator	DSMT0013 (CAK51094)	93/95
CB00072_orf1	470	Flavoprotein	DSMT0011 (CAK51092)	82/88
CB00072_orf2	431	Hypothetical protein	SLIV_04130 (AIJ11849)	53/62
CB00072_orf3	192	Transporter	SGLAU_00240 (AIR96076)	82/89
CB00072_orf4	95	Hypothetical protein	SACT1_1507 (EGE40872)	96/98
CB00072_orf5	244	Hypothetical protein	SGLAU_00235 (AIR96075)	79/86
CB00072_orf6	121	Sulfurtransferase	FM21_33875 (KFG71729)	74/82
CB00072_orf7	454	Beta-lactamase	IX27_29110 (KFK86222)	84/89
CB00072_orf8	590	Glucoamylase	DF19_10205 (KDN72532)	71/81
CB00072_orf9	249	Methyltransferase	DF19_29950 (KDN79623)	72/79
CB00072_orf10	417	Aminotransferase	SPW_3219 (EHM28434)	76/84
CB00072_orf11	1063	Aminotransferase	SPW_3218 (EHM28433)	76/84
CB00072_orf12	547	PA14 domain-containing protein	SPW_3217 (EHM28432)	96/97
CB00072_orf13	335	UDP-glucuronic acid decarboxylase	SgcA (AAL06671)	32/47
CB00072_orf14	426	UDP-glucose 6-dehydrogenase	MdpA2 (ABY66024)	32/43
CB00072_orf15	351	UDP-glucose 4-epimerase	SgcA (AAL06671)	31/41
CB00072_orf16	608	*N*-acetylglucosaminyltransferase	BU52_16600 (KES06039)	63/74
CB00072_orf17	246	TetR family transcriptional regulator	STAFG_1716 (EPJ41222)	64/73
CB00072_orf18	415	Hypothetical protein	SGR_423 (BAG17252)	80/86
CB00072_orf19	411	Rieske (2Fe-2S) iron-sulfur domain protein	SFUL_443 (AGK75427)	87/90
CB00072_orf20	214	AraC family transcriptional regulator	MdpR2 (ABY65996)	51/61
CB00072_E6	194	Flavin reductase	NcsE6 (AAM78010)	57/64
CB00072_E10	150	Type II thioesterase	NcsE10 (AAM78011)	77/83
CB00072_E	1944	Enediyne polyketide synthase	NcsE (AAM78012)	64/73
CB00072_E5	326	Unknown	NcsE5 (AAM78013)	70/81
CB00072_E4	635	Unknown	NcsE4 (AAM78014)	66/76
CB00072_E3	311	Unknown	NcsE3 (AAM78015)	54/64
CB00072_orf27	250	Hypothetical protein	Cynorf27 (AGO97171)	55/69
CB00072_orf28	364	Hypothetical protein	BN6_37270 (CCH31018)	67/83
CB00072_orf29	271	Methyltransferase	PsmC (AHL44341)	38/51
CB00072_orf30	246	Oxidoreductase	KedU16 (AFV52172)	34/47
CB00072_orf31	156	Activator of HSP90 ATPase	SPW_3199 (EHM28414)	99/99
CB00072_orf32	170	Hypothetical protein	CalU16 (AAM70339)	34/45
CB00072_orf33	426	Major facilitator superfamily transporter	SalK (ACF40866)	26/44
CB00072_orf34	148	Hypothetical protein	SCLAV_p1238 (EFG04724)	68/80
CB00072_orf35	214	Transcriptional regulator	CalU8 (AAM94775)	46/61
CB00072_orf36	398	PBS lyase HEAT-like repeat protein	Dynorf16 (ACB47059)	29/45
CB00072_orf37	447	Putative regulator	DynR3 (ACB47054)	47/60
CB00072_orf38	244	Hypothetical protein	MCAG_00208 (EEP69881)	46/56
CB00072_orf39	192	Alkylhydroperoxidase	Sgcorf58 (AAL06711)	68/76
CB00072_orf40	187	Unknown	SgcS (AAL06705)	84/91
CB00072_orf41	147	Unknown	SgcT (AAL06706)	78/82
CB00072_orf(+1)	1150	Oxidoreductase	ChxJ (AFO59871)	89/92

[a]*orf(-1)* and *orf(+1)* are predicted to represent the upstream and downstream boundaries of the enediyne gene cluster.
[b]Number of amino acids.
[c]Also see Figure 4 and Figure 2C for the organization of the enediyne gene cluster.
[d]Selected strains from the same clade (Figure 2A, panel B) that have been sequenced, yielding highly homologous gene clusters (also see Tables S4-14, S4-15, S4-16). Only the cluster from CB00072 was shown in Figure 2C, but all four homologous gene clusters are included in the enediyne GNN (Figure 4).

Table S4-18. Related to Figures 2, 4. Predicted functions of ORFs in the enediyne biosynthetic gene cluster from *Streptomyces* sp. TSRI0445

gene[a]	aa[b]	putative function[c]	protein homologue	% identity/ % similarity
TSRI0445_orf(-1)	86	Transposase, partial	WP_030192830	78/86
TSRI0445_orf1	432	Serine/threonine kinase	SHJGH_1651 (AGF61317)	47/59
TSRI0445_orf2	215	Hypothetical protein	VAB18032_17895 (AEB44681)	49/65
TSRI0445_orf3	214	Hypothetical protein	BN6_41430 (CCH31430)	35/50
TSRI0445_orf4	1208	Serine/threonine protein kinase	Z951_15810 (EXU67304)	42/54
TSRI0445_orf5	266	Hypothetical protein	WP_030523012	40/54
TSRI0445_orf6	217	Transposase	SSBG_01101 (EDY43139)	80/86
TSRI0445_orf7	244	AraC family transcriptional regulator	NcsR6 (AAM78008)	44/57
TSRI0445_E6	194	Flavin reductase	NcsE6 (AAM78010)	60/65
TSRI0445_E10	150	Type II thioesterase	NcsE10 (AAM78011)	80/86
TSRI0445_E	1954	Enediyne polyketide synthase	SgcE (AAL06699)	66/76
TSRI0445_E5	324	Unknown	NcsE5 (AAM78013)	69/81
TSRI0445_E4	635	Unknown	NcsE4 (AAM78014)	65/76
TSRI0445_E3	323	Unknown	NcsE3 (AAM78015)	65/76
TSRI0445_orf14	250	Hypothetical protein	CynORF27 (AGO97171)	55/68
TSRI0445_orf15	370	Hypothetical protein	SPW_3202 (EHM28417)	95/97
TSRI0445_orf16	269	Methyltransferase	SPW_3201 (EHM28416)	86/93
TSRI0445_orf17	244	Short chain dehydrogenase	KedU16 (AFV52172)	35/47
TSRI0445_orf18	156	Activator of HSP90 ATPase	SPW_3199 (EHM28415)	92/97
TSRI0445_orf19	170	Hypothetical protein	CalU16 (AAM70339)	35/47
TSRI0445_orf20	425	Major facilitator transporter	SPW_3197 (EHM28412)	94/96
TSRI0445_orf21	148	Hypothetical protein	SPW_3196 (EHM28411)	94/96
TSRI0445_orf22	214	HxlR family transcriptional regulator	DynU8 (ACB47051)	45/58
TSRI0445_orf23	398	PBS lyase HEAT-like repeat protein	DynORF16 (ACB47059)	30/46
TSRI0445_orf24	447	Unknown	DynR3 (ACB47054)	47/61
TSRI0445_orf25	244	Hypothetical protein	SPW_3192 (EHM28407)	82/86
TSRI0445_orf26	183	Hypothetical protein	SgcO (WP_010056303)	79/86
TSRI0445_orf27	187	Unknown	SgcS (AAL06705)	87/94
TSRI0445_orf28	156	Unknown	SgcT (AAL06706)	76/85
TSRI0445_orf29	235	Hypothetical protein SPW	SPW_5764 (EHM25843)	45/56
TSRI0445_orf(+1)	300	Chitinase	ChiF (BAA75646)	69/79

[a]*orf(-1)* and *orf(+1)* are predicted to represent the upstream and downstream boundaries of the enediyne gene cluster.
[b]Number of amino acids.
[c]Also see Figure 4 and Figure 2C for the organization of the enediyne gene cluster.

Table S4-19. Related to Figures 2, 4. Predicted functions of ORFs in the enediyne biosynthetic gene cluster (partial) from *Micromonospora* sp. CB01531

gene	aa[a]	putative function[b]	protein homologue	% identity/ % similarity
CB01531_orf1	282	Hypothetical protein	LQ51_28645 (KKJ93962)	73/79
CB01531_orf2	367	Hypothetical protein	LQ51_28650 (KKJ93983)	82/87
CB01531_orf3	140	Putative glyoxalase	VAB18032_18715 (AEB44845)	78/89
CB01531_orf4	233	AraC family transcriptional regulator	Spoorf3 (ABP55137)	58/74
CB01531_E7	454	Cytochrome P450	SpoE7 (ABP55138)	68/78

CB01531_E6	178	Flavin reductase	SpoE6 (ABP55139)	71/81
CB01531_E10	147	Type II thioesterase	CyaE10 (AGO97217)	87/92
CB01531_E	1922	Enediyne polyketide synthase	SpoE (ABP55141)	73/82
CB01531_E5	329	Unknown	SpoE5 (ABP55142)	79/87
CB01531_E4	657	Unknown	SpoE4 (ABP55143)	76/86
CB01531_E3	296	Unknown	SpoE3 (ABP55144)	65/73
CB01531_E2	332	Unknown	SpoE2 (ABP55145)	68/77
CB01531_orf13	359	Unknown	Spoorf5 (ABP55147)	44/58
CB01531_M	370	Unknown	MdpM (ABY66000)	56/65
CB01531_E1	153	HxlR family transcriptional regulator	SgcE1 (AAL06704)	56/70
CB01531_E9	551	Oxidoreductase	SpoE9 (ABP55148)	85/92
CB01531_E8	176	Unknown	SpoE8 (ABP55149)	74/81
CB01531_orf18	198	Unknown	CalU21 (AAM70363)	66/82
CB01531_orf19	203	Unknown	CalU20 (AAM70362)	41/52
CB01531_E11	267	Unknown	CyaE11 (AGO97209)	68/78
CB01531_orf21	152	Putative chorismate mutase	AroH (AAO40746)	44/66
CB01531_orf22	143	Putative SAM binding protein	AF_0433 (O29816)	45/57
CB01531_orf23	209	Hypothetical protein	Mb2612c (P64262)	31/48
CB01531_orf24	354	Zinc-containing alcohol dehydrogenase	SpoT5 (ABP55167)	29/42

[a]Number of amino acids.
[b]Also see Figure 4 and Figure 2C for the organization of the enediyne gene cluster.

Table S4-20. Related to Figures 2, 4. Predicted functions of ORFs in the enediyne biosynthetic gene cluster from *Micromonospora* sp. TSRI0369

gene[a]	aa[b]	putative function[c]	protein homologue	% identity/ % similarity
TSRI0369_orf(-1)	287	Hypothetical protein	Micau_1945 (ADL45493)	84/88
TSRI0369_orf1	602	Hypothetical protein	WP_030503336	89/90
TSRI0369_orf2	242	Unknown	CalU12 (AAM94790)	71/77
TSRI0369_orf3	467	NDP-hexose 2,3-dehydratase	KedS2 (AFV52155)	45/57
TSRI0369_orf4	158	Activator of Hsp90 ATPase	ML5_2051 (ADU07577)	90/94
TSRI0369_orf5	274	ABC transporter permease	CalT4 (AAM70340)	43/62
TSRI0369_orf6	328	ABC transporter ATP-binding protein	CalT5 (AAM70341)	57/74
TSRI0369_orf7	274	Methyltransferase	CalE5 (AAM94788)	35/47
TSRI0369_orf8	410	*C*-3'-Methyltransferase	MdpA4 (ABY66027)	60/71
TSRI0369_orf9	250	SAM-dependent methyltransferase	CalS10 (AAM70334)	44/56
TSRI0369_orf10	328	NDP-4-keto-6-deoxyhexose reductase	NcsC4 (AAM77980)	40/61
TSRI0369_orf11	371	Glycosyltransferase	CalG3 (AAM94798)	39/53
TSRI0369_orf12	584	Hypothetical protein	MCBG_01985 (EWM64852)	98/98
TSRI0369_orf13	369	NDP-hexose aminotransferase	MdpA5 (ABY66023)	33/48
TSRI0369_orf14	181	Pyridoxamine 5-phosphate oxidase	MCBG_01983 (EWM64850)	98/99
TSRI0369_orf15	447	Cytochrome P450	CynE7 (AGO97155)	29/44
TSRI0369_orf16	180	Hypothetical protein	O3I_023225 (AFU02600)	59/72
TSRI0369_orf17	297	Unknown	CalE5 (AAM94788)	44/55
TSRI0369_orf18	232	Copper resistance protein C	SacmaDRAFT_0229 (EHR48539)	40/49
TSRI0369_orf19	187	Unknown	CalU21 (AAM70363)	67/77
TSRI0369_E6	187	Flavin reductase	NcsE6 (AAM78010)	62/72
TSRI0369_E10	149	Type II thioesterase	SgcE10 (pdb|4I4J|A)	61/72
TSRI0369_E	1925	Enediyne polyketide synthase	NcsE (AAM78012)	55/66
TSRI0369_E5	323	Unknown	NcsE5 (AAM78013)	69/78
TSRI0369_E4	650	Unknown	NcsE4 (AAM78014)	55/68

TSRI0369_E3	303	Unknown	NcsE3 (AAM78015)	51/61
TSRI0369_orf26	291	AraC family transcriptional regulator	CalR7 (AAM70331)	47/58
TSRI0369_orf27	329	Adenosine deaminase	CalS5 (AAM94774)	47/55
TSRI0369_orf28	827	ATP-dependent Clp protease	CalR4 (AAM94782)	98/98
TSRI0369_orf29	634	Transcription activator	CalR2 (AAM94777)	40/55
TSRI0369_orf30	399	Unknown	CalU10 (AAM94783)	70/84
TSRI0369_orf31	298	Unknown	CalE3 (AAM94778)	61/69
TSRI0369_orf32	213	Unknown	CalU9 (AAM94779)	39/58
TSRI0369_orf33	428	Unknown	CalR3 (AAM94780)	64/75
TSRI0369_orf34	234	HxlR family transcriptional regulator	CalU8 (AAM94775)	45/61
TSRI0369_orf35	425	Cysteine desulfurase	CalE4 (AAM94786)	67/76
TSRI0369_orf36	379	Cystathione gamma lyase	CalE6 (AAM94792)	64/73
TSRI0369_orf37	300	Homocysteine methyltransferase	CalE2 (AAM94773)	55/63
TSRI0369_orf38	217	Hypothetical protein	CalU13 (AAM94791)	58/69
TSRI0369_orf39	445	Aminotransferase V	CalE9 (AAM94796)	71/82
TSRI0369_orf40	564	ABC transporter substrate-binding protein	CalT7 (AAM70346)	48/58
TSRI0369_orf41	410	Unknown	CalR9 (AAM70347)	63/73
TSRI0369_orf42	570	ABC transporter substrate-binding protein	CalT6 (AAM70342)	57/70
TSRI0369_orf43	575	ABC transporter substrate-binding protein	CalT6 (AAM70342)	39/52
TSRI0369_orf44	260	Unknown	CalU11 (AAM94787)	56/65
TSRI0369_orf45	457	Peptidase	CalR5 (AAM94784)	49/60
TSRI0369_orf46	488	Modulator of DNA gyrase	CalR6 (AAM94785)	56/67
TSRI0369_orf(+1)	423	Cellulose-binding family II protein	Micau_1946 (ADL45494)	84/86

[a]*orf(-1)* and *orf(+1)* are predicted to represent the upstream and downstream boundaries of the enediyne gene cluster.
[b]Number of amino acids.
[c]Also see Figure 4 and Figure 2C for the organization of the enediyne gene cluster.

Table S4-21. Related to Figures 2, 4. Predicted functions of ORFs in the enediyne biosynthetic gene cluster from *Streptomyces* sp. CB01249

gene[a]	aa[b]	putative function[c]	protein homologue	% identity/ % similarity
CB01249_orf(-1)	272	Hydrolase	SBI_00645 (ADI03766)	73/84
CB01249_orf1	288	AraC family transcriptional regulator	SSTG_05665 (EFF88658)	78/84
CB01249_E11	265	Unknown	NcsE11 (AAM78004)	55/65
CB01249_E8	185	Unknown	NcsE8 (AAM78006)	55/68
CB01249_E9	550	Oxidoreductase	MdpE9 (ABY65999)	78/86
CB01249_M	367	Unknown	MdpM (ABY66000)	50/60
CB01249_orf6	327	Transcriptional regulator	NcsR6 (AAM78007)	40/55
CB01249_orf7	280	AraC family transcriptional regulator	NcsR5 (AAM78008)	57/72
CB01249_orf8	407	Transcriptional regulator	NcsR7 (AAM78019)	53/64
CB01249_orf9	198	TetR family transcriptional regulator	NcsR3 (AAM78020)	47/64
CB01249_E7	456	Cytochrome P450	NcsE7 (AAM78009)	57/71
CB01249_E3	327	Unknown	SgcE3 (AAL06702)	55/67
CB01249_E2	332	Unknown	NcsE2 (AAM78016)	61/70
CB01249_E6	181	Flavin reductase	NcsE6 (AAM78010)	47/60
CB01249_E10	157	Type II thioesterase	KedE10 (AFV52146)	68/76
CB01249_E	1965	Enediyne polyketide synthase	KedE (AFV52145)	57/68
CB01249_E5	331	Unknown	KedE5 (AFV52140)	51/69
CB01249_E4	643	Unknown	KedE4 (AFV52141)	51/64
CB01249_orf18	215	Transcriptional repressor	CynR2 (AGO97151)	53/66

CB01249_orf19	451	Dioxygenase	STTU_4287 (EGJ77076)	57/66
CB01249_orf20	515	Hypothetical protein	MPTA5024_35235 (ETK31428)	60/72
CB01249_orf21	218	Maleylpyruvate isomerase	SU9_04126 (EJJ08333)	74/82
CB01249_orf22	131	Hypothetical protein	SVEN_3959 (CCA57245)	58/68
CB01249_orf23	148	Transcriptional regulator	NONO_c68770 (AHH21644)	72/83
CB01249_orf24	229	SnoaL-like domain-containing protein	NONO_c68760 (AHH21643)	58/71
CB01249_J	149	Unknown	KedJ (AFV52149)	41/53
CB01249_orf26	250	Oxidoreductase	KedU16 (AFV52172)	58/68
CB01249_orf27	263	Type II thioesterase	KedU43 (AFV52205)	39/55
CB01249_orf(+1)	871	LuxR family transcriptional regulator	SlnR (AEZ53964)	49/58

[a]*orf(-1)* and *orf(+1)* are predicted to represent the upstream and downstream boundaries of the enediyne gene cluster.
[b]Number of amino acids.
[c]Also see Figure 4 and Figure 2C for the organization of the enediyne gene cluster.

Table S4-22. Related to Figures 2, 4. Predicted functions of ORFs in the enediyne biosynthetic gene cluster from *Streptomyces* sp. CB03911

gene[a]	aa[b]	putative function[c]	protein homologue	% identity/ % similarity
CB03911_orf(-1)	379	ABC transporter permease	H340_14231 (EME99882)	69/80
CB03911_orf1	168	Amino-acid acetyltransferase	STRAU_1250 (EPH45669)	49/58
CB03911_orf2	270	ABC transporter ATP-binding protein	CalT5 (AAM70341)	30/49
CB03911_orf3	210	Isochorismatase	KCH_06130 (KDN87600)	74/82
CB03911_orf4	530	AMP-binding protein	NcsB2 (AAM77987)	48/62
CB03911_orf5	395	Isochorismate synthase	ADK60_09510 (KOV35336)	63/71
CB03911_orf6	249	Short chain dehydrogenase	KedU16 (AFV52172)	38/48
CB03911_orf7	769	5-Methyltetrahydropteroyltriglutamate-homocysteine methyltransferase	UK12_04330 (KJK59434)	89/93
CB03911_orf8	357	N(6)-L-threonylcarbamoyladenine synthase	ADK60_35200 (KOV11490)	89/94
CB03911_orf9	801	UvrA-like drug resistance pump	SgcB2 (AAL06654)	49/64
CB03911_orf10	136	Glyoxalase/bleomycin resistance protein/dioxygenase	DynE15 (ACB47077)	39/47
CB03911_orf11	147	AraC family transcriptional regulator	GZL_01436 (AJC54036)	85/93
CB03911_orf12	299	Hypothetical protein	SNA_21275 (KIZ15831)	54/64
CB03911_orf13	171	RNA polymerase subunit sigma-24	HS99_41870 (KND10017)	64/75
CB03911_orf14	154	Hypothetical protein	ADK51_00990 (KOU37210)	59/71
CB03911_orf15	347	NAD-dependent epimerase/dehydratase	SpoT8 (ABP55146)	60/69
CB03911_orf16	257	Hypothetical protein	ASC99_33065 (KQV13633)	68/75
CB03911_orf17	432	Unknown	CalR3 (AAM94780)	44/57
CB03911_orf18	255	AraC family transcriptional regulator	SgcR2 (AAL06696)	40/57
CB03911_orf19	454	Dioxygenase	AWW66_03690 (KXK63420)	58/70
CB03911_orf20	199	Transcriptional repressor	CynR2 (AGO97151)	58/73
CB03911_orf21	325	Hypothetical protein	KedE1 (AFV52144)	58/72
CB03911_E3	326	Unknown	KedE3 (AFV52142)	58/67
CB03911_E7	457	Cytochrome P450	MdpE7 (AAQ17113)	66/77
CB03911_E6	184	Flavin reductase	NcsE6 (AAM78010)	53/64
CB03911_E10	162	Type II thioesterase	CynE10 (AGO97148)	77/85
CB03911_E	1960	Enediyne polyketide synthase	CynE (AGO97147)	57/68
CB03911_E5	338	Unknown	CyaE5 (AGO97182)	61/73
CB03911_E4	646	Unknown	CyaE4 (AGO97181)	55/67
CB03911_orf29	516	Choline dehydrogenase	CynN2 (AGO97168)	35/51
CB03911_orf30	233	Protein of unknown function DUF899	Spoorf25 (ABP55180)	47/63

CB03911_orf31	123	ArsR family transcriptional regulator	CynR6 (AGO97176)	44/58
CB03911_orf32	210	Peroxidase	ASC99_32500 (KQV13762)	88/94
CB03911_orf33	291	Short-chain dehydrogenase	ASC99_32505 (KQV13763)	92/97
CB03911_orf34	262	Hypothetical protein	ASC99_32510 (KQV13764)	73/83
CB03911_M	334	Unknown	SgcM (AAL06686)	50/59
CB03911_orf36	255	Regulatory protein	Spoorf3 (ABP55137)	41/55
CB03911_orf37	314	ABC transporter permease	KSE_45590 (BAJ30340)	71/83
CB03911_orf38	297	ABC transporter ATP-binding protein	DynT5 (ACB47076)	35/52
CB03911_orf39	435	Cytochrome P450 hydroxylase	SgcD3 (AAL06684)	31/45
CB03911_orf40	272	Hypothetical protein	KSE_45630 (BAJ30344)	59/71
CB03911_orf41	782	Putative toxin ABC transporter ATP-binding and permease protein	KCH_45500 (KDN83901)	80/87
CB03911_orf42	738	ABC transporter ATP-binding protein/permease	KSE_45650 (BAJ30346)	76/83
CB03911_orf43	1106	Hypothetical protein	KSE_45660 (BAJ30347)	69/77
CB03911_orf44	757	Hypothetical protein	ASC99_06355 (KQV18807)	88/92
CB03911_orf45	496	Hypothetical protein	ASC99_06350 (KQV18806)	91/94
CB03911_orf(+1)	222	Putative response regulator	KCH_45580 (KDN83909)	70/78

[a]*orf(-1)* and *orf(+1)* are predicted to represent the upstream and downstream boundaries of the enediyne gene cluster.
[b]Number of amino acids.
[c]Also see Figure 4 and Figure 2C for the organization of the enediyne gene cluster.

Table S4-23. Related to Figures 2, 4. Predicted functions of ORFs in the enediyne biosynthetic gene cluster from *Streptomyces* sp. TSRI0107

gene[a]	aa[b]	putative function[c]	protein homologue	% identity/ % similarity
TSRI0107_orf(-1)	323	*N*-Acetyltransferase GCN5	SGLAU_31165 (AIS02167)	85/89
TSRI0107_orf1	389	Luciferase	Spoorf10 (ABP55156)	64/78
TSRI0107_orf2	423	Sugar dehydrogenase	UK12_21795 (KJK56573)	84/91
TSRI0107_orf3	387	Myo-inositol-1-phosphate synthase	AMETH_2780 (AIJ22872)	65/74
TSRI0107_orf4	306	4-Hydroxybenzoate polyprenyltransferase	SacmaDRAFT_1518 (EHR49794)	58/67
TSRI0107_orf5	288	Sugar phosphate isomerase	BB31_30115 (AJK57390)	66/74
TSRI0107_orf6	209	Sugar phosphate isomerase/epimerase	BB31_30115 (AJK57390)	59/67
TSRI0107_orf7	286	TatD family hydrolase	SacglDRAFT_01227 (EIE98158)	79/87
TSRI0107_orf8	367	Xylose isomerase	BB31_30125 (AJK57392)	62/73
TSRI0107_orf9	463	Phosphodiesterase	SacmaDRAFT_1522 (EHR49798)	73/82
TSRI0107_orf10	531	Hypothetical protein	MPTA5024_05320 (ETK37149)	65/75
TSRI0107_F	385	Epoxide hydrolase	NcsF2 (AAM78002)	62/72
TSRI0107_orf12	463	FAD-binding monooxygenase	KedD2 (AFV52150)	58/70
TSRI0107_J	138	Unknown	SgcJ (AAL06676)	55/73
TSRI0107_orf14	391	Oxidoreductase	SgcL (AAL06685)	60/73
TSRI0107_orf15	412	Cytochrome P450 hydroxylase	NcsB3 (AAM77997)	55/69
TSRI0107_orf16	338	Zn-dependent alcohol dehydrogenase	MdpC8 (ABY66028)	66/76
TSRI0107_orf17	122	YCII-related protein	Spoorf12 (ABP55159)	43/64
TSRI0107_orf18	262	3-Oxoacyl-ACP reductase	KedU16 (AFV52172)	30/46
TSRI0107_orf19	417	RNA polymerase subunit sigma-24	Spoorf11 (ABP55158)	55/66
TSRI0107_orf20	514	Major facilitator superfamily MFS_1	SgcB (AAL06672)	47/66
TSRI0107_orf21	65	Ferredoxin	H480_06778 (EOD69287)	53/70
TSRI0107_orf22	405	Cytochrome P450	CalE10 (AAM94800)	46/61
TSRI0107_orf23	396	Acyltransferase	CynA4 (AGO97170)	36/52
TSRI0107_orf24	122	Hypothetical protein	Spoorf12 (ABP55159)	41/60

TSRI0107_orf25	461	Oxidoreductase	SpoT4 (ABP55175)	30/45
TSRI0107_orf26	412	Acyl-CoA dehydrogenase	Spoorf17 (ABP55164)	74/84
TSRI0107_orf27	462	C-domain type II peptide synthetase	SgcC5 (AAL06678)	44/62
TSRI0107_orf28	445	Glycerol phosphate ABC transporter	SgcB1 (AAL06653)	48/61
TSRI0107_orf29	524	Monooxygenase	MdpC (ABY66026)	80/88
TSRI0107_orf30	593	Non-ribosomal peptide synthetase	SpoT2 (ABP55169)	62/73
TSRI0107_orf31	438	4-Hydroxyphenylpyruvate dioxygenase	SpoT1 (ABP55171)	54/67
TSRI0107_orf32	297	Hypothetical protein	SpoT7 (ABP55172)	65/78
TSRI0107_orf33	507	FAD-linked oxidase	SpoT4 (ABP55175)	26/38
TSRI0107_orf34	258	Short-chain dehydrogenase	KedU16 (AFV52172)	31/45
TSRI0107_orf35	239	Unknown	Spoorf22 (ABP55176)	52/68
TSRI0107_orf36	167	Short-chain dehydrogenase	MCAG_00219 (EEP69892)	31/50
TSRI0107_orf37	635	Hydroxylase-like protein	SgcORF-7 (AAL06648)	73/80
TSRI0107_orf38	384	Hypothetical protein	SGR_596 (BAG17425)	73/84
TSRI0107_orf39	186	Unknown	SgcS (AAL06705)	71/82
TSRI0107_orf40	165	Unknown	SgcT (AAL06706)	56/66
TSRI0107_orf41	179	Hypothetical protein	SgcORF58 (AAL06711)	66/75
TSRI0107_orf42	447	Carotenoid oxygenase	Sim14 (EMF53822)	45/58
TSRI0107_orf43	201	Transcriptional repressor	CynR2 (AGO97151)	57/71
TSRI0107_E6	186	Flavin reductase	SgcE6 (AAL06698)	53/67
TSRI0107_E10	166	Type II thioesterase	SgcE10 (AAL06692)	72/84
TSRI0107_E	1956	Enediyne polyketide synthase	SgcE (AAL06699)	57/67
TSRI0107_E5	333	Unknown	SgcE5 (AAL06700)	55/68
TSRI0107_E4	652	Unknown	MdpE4 (AAQ17108)	50/64
TSRI0107_E11	266	Unknown	NcsE11 (AAM78004)	54/67
TSRI0107_E2	340	Unknown	MdpE2 (ABY65995)	64/73
TSRI0107_E3	326	Unknown	MdpE3 (AAQ17107)	57/69
TSRI0107_E7	454	Cytochrome P450	MdpE7 (AAQ17113)	68/76
TSRI0107_orf53	238	AraC family transcriptional regulator	NcsR5 (AAM78008)	51/65
TSRI0107_E8	189	Unknown	NcsE8 (AAM78006)	64/75
TSRI0107_E9	551	Oxidoreductase	NcsE9 (AAM78005)	80/88
TSRI0107_M	364	Unknown	SgcM (AAL08886)	45/51
TSRI0107_orf57	220	MerR family transcriptional regulator	Spoorf2 (ABP55136)	39/49
TSRI0107_orf58	322	Unknown	CalU6 (AAM94767)	47/61
TSRI0107_orf(+1)	726	Hypothetical protein	SSQG_06405 (EFL35887)	62/68

[a]*orf(-1)* and *orf(+1)* are predicted to represent the upstream and downstream boundaries of the enediyne gene cluster.
[b]Number of amino acids.
[c]Also see Figure 4 and Figure 2C for the organization of the enediyne gene cluster.

Table S4-24. Related to Figures 2, 4. Predicted functions of ORFs in the enediyne biosynthetic gene cluster (partial) from *Streptomyces* sp. CB02923

gene[a]	aa[b]	putative function[c]	protein homologue	% identity/ % similarity
CB02923_orf(-1)	129	Putative cell division protein	SepF (Q93JG0)	72/80
CB02923_orf1	386	Putative transport protein	CorA (Q9WZ31)	27/47
CB02923_orf2	211	MerR family transcriptional regulator	CyaR5 (AGO97221)	38/54
CB02923_orf3	160	Short-chain dehydrogenase	MCAG_00219 (EEP69892)	31/44
CB02923_orf4	263	AraC family transcriptional regulator	SgcR2 (AAL06696)	42/56
CB02923_orf5	122	Hypothetical protein	Spoorf16 (ABP55163)	45/60
CB02923_orf6	418	RNA polymerase subunit sigma-24	Spoorf11 (ABP55158)	53/64
CB02923_orf7	122	YCII-related protein	Spoorf12 (ABP55159)	51/69

CB02923_E6	182	Flavin reductase	KedE6 (AFV52147)	60/72
CB02923_E10	153	Type II thioesterase	CynE10 (AGO97148)	77/87
CB02923_E	1967	Enediyne polyketide synthase	MdpE (AAQ17110)	57/67
CB02923_E5	322	Unknown	CyaE5 (AGO97223)	63/76
CB02923_E4	651	Unknown	CyaE4 (AGO97222)	53/65
CB02923_orf13	618	Non-ribosomal peptide synthetase	SpoT2 (ABP55169)	58/71
CB02923_orf14	76	Putative chaperone	MbtH (P59965)	61/70
CB02923_orf15	358	4-Hydroxyphenylpyruvate dioxygenase	SpoT1 (ABP55171)	50/63
CB02923_orf16	285	Hypothetical protein	SpoT7 (ABP55172)	59/73
CB02923_orf17	507	Putative methyltransferase	SpoT6 (ABP55177)	52/64
CB02923_orf18	507	FAD-linked oxidase	SpoT4 (ABP55175)	25/38
CB02923_orf19	257	Short-chain dehydrogenase	KedU16 (AFV52172)	32/46
CB02923_orf20	232	Unknown	Spoorf22 (ABP55176)	54/74
CB02923_orf21	535	Major facilitator superfamily permease	Spoorf15 (ABP55162)	62/76
CB02923_orf22	334	Zinc-containing alcohol dehydrogenase	SpoT5 (ABP55167)	59/73
CB02923_orf23	413	Cytochrome P450	SpoT9 (ABP55168)	66/77
CB02923_orf24	381	Oxidoreductase	Spoorf7(ABP55152)	62/78
CB02923_J	137	Unknown	SgcJ (AAL06676)	52/69
CB02923_orf26	457	FAD-dependent monooxygenase	Spoorf9(ABP55154)	67/77
CB02923_F	384	Epoxide hydrolase	SpoF1(ABP55155)	72/82
CB02923_orf28	548	Monooxygenase	MdpC3 (ABY66026)	76/86
CB02923_orf29	440	ABC transporter	Spoorf18 (ABP55166)	60/70
CB02923_orf30	416	Oxidoreductase	Spoorf10 (ABP55156)	66/81
CB02923_orf31	445	C-domain type II peptide synthetase	SpoT10 (ABP55165)	55/70
CB02923_orf32	414	Hydroxylase	Spoorf17 (ABP55164)	65/76
CB02923_orf33	460	Oxidoreductase	SpoT4 (ABP55175)	28/42
CB02923_E11	293	Unknown	SpoE11 (ABP55150)	57/68
CB02923_E2	338	Unknown	MdpE2 (ABY65995)	63/71
CB02923_E3	326	Unknown	KedE3 (AFV52142)	61/68
CB02923_E7	455	Cytochrome P450	KedE7 (AFV52138)	65/76
CB02923_E8	189	Unknown	MdpE8 (ABY65998)	65/73
CB02923_E9	553	Putative ketoreductase	KedE9 (AFV52135)	81/89
CB02923_orf40	355	Unknown	Spoorf5 (ABP55147)	49/62
CB02923_orf41	552	MFS transporter	AQJ91_35690 (KUO16453)	53/67

[a]*orf(-1)* is predicted to represent the upstream boundary of the enediyne gene cluster.
[b]Number of amino acids.
[c]Also see Figure 4 and Figure 2C for the organization of the enediyne gene cluster.

Table S4-25. Related to Figures 2, 4. Predicted functions of ORFs in the enediyne biosynthetic gene cluster from *Streptomyces* sp. CB02488

gene[a]	aa[b]	putative function[c]	protein homologue	% identity/ % similarity
CB02488_orf(-1)	357	PEP-utilizing protein	SACT1_0691 (EGE40080)	74/84
CB02488_orf1	292	AraC family transcriptional regulator	SgcR2 (AAL06696)	39/56
CB02488_orf2	257	Unknown	NcsORF55 (AAM78024)	69/79
CB02488_orf3	274	Enoyl-CoA hydratase/isomerase	SACE_2794 (CAM02075)	42/59
CB02488_orf4	516	AMP-dependent synthetase and ligase	KedU37 (AFV52199)	31/45
CB02488_orf5	232	Protein of unknown function DUF899	Spoorf25 (ABP55180)	51/64
CB02488_orf6	248	Short-chain dehydrogenase	BN973_00665 (CDO86323)	55/64
CB02488_orf7	315	Hypothetical protein	C791_5076 (EMD25067)	59/70

CB02488_orf8	394	Oxidoreductase	MdpD2 (ABY66021)	59/75
CB02488_orf9	238	NADP oxidoreductase	DF18_20635 (KEF18816)	61/75
CB02488_orf10	135	Glutathione-dependent formaldehyde-activating	SSRG_00053 (EFL37249)	54/63
CB02488_orf11	167	Pyridoxamine 5-phosphate oxidase	MCAG_04115 (EEP73788)	56/72
CB02488_orf12	113	Glyoxalase	Sare_3170 (ABV98977)	46/63
CB02488_orf13	148	Activator of HSP90 ATPase	STRTUCAR8_03592 (ELP68531)	74/83
CB02488_orf14	118	ArsR family transcriptional regulator	CynR6 (AGO97176)	43/57
CB02488_orf15	478	MFS transporter	NcsA2 (AAM77999)	33/52
CB02488_orf16	461	Dioxygenase	STTU_4287 (EGJ77076)	56/65
CB02488_orf17	216	Transcriptional repressor	MdpORF-3 (ABY66033)	60/71
CB02488_orf18	319	Unknown	NcsORF56 (AAM78025)	53/68
CB02488_E3	327	Unknown	SgcE3 (AAL06702)	52/64
CB02488_E7	463	Cytochrome P450 hydroxylase	SgcE7 (AAL06697)	59/73
CB02488_E6	189	Flavin reductase	SgcE6 (AAL06698)	53/63
CB02488_E10	158	Type II thioesterase	SgcE10 (AAL06692)	77/85
CB02488_E	1953	Enediyne polyketide synthase	SgcE (AAL06699)	56/67
CB02488_E5	339	Unknown	SgcE5 (AAL06700)	53/66
CB02488_E4	660	Unknown	MdpE4 (AAQ17108)	50/61
CB02488_orf26	515	Choline dehydrogenase	CynN2 (AGO97168)	36/51
CB02488_orf27	211	Peroxidase	Amir_4098 (ACU37954)	59/76
CB02488_orf28	266	Short-chain dehydrogenase	KedU16 (AFV52172)	31/46
CB02488_M	351	Unknown	SgcM (AAL06686)	44/54
CB02488_orf30	265	AraC family transcriptional regulator	SgcR2 (AAL06696)	38/53
CB02488_orf31	233	Hypothetical protein	SPW_3192 (EHM28407)	49/58
CB02488_orf32	344	NAD-dependent epimerase	CyaORF24 (AGO97213)	50/62
CB02488_orf33	436	Unknown	DynR3 (ACB47054)	46/59
CB02488_E11	260	Unknown	NcsE11 (AAM78004)	47/62
CB02488_orf35	415	MFS transporter	DC60_11245 (KDR63745)	67/81
CB02488_orf36	791	Protein phosphatase	XNR_3724 (AGI90059)	58/69
CB02488_orf37	544	Transporter	SAV_1370 (BAC69080)	42/60
CB02488_orf38	258	Glycosyl hydrolase	SSPG_00638 (EFD64998)	71/81
CB02488_orf39	173	Hypothetical protein	NFA_48050 (BAD59657)	38/52
CB02488_orf40	423	Oxidoreductase	MdpD2 (ABY66021)	33/48
CB02488_orf41	214	TetR family transcriptional regulator	Z951_02415 (EXU69849)	54/70
CB02488_orf42	580	Hydrolase	HY68_35010 (KIF67002)	84/91
CB02488_orf43	288	Nucleoside-diphosphate sugar epimerase	FG99_15935 (KES23054)	64/74
CB02488_orf44	352	Membrane protein	STTU_5012 (EGJ77801)	61/73
CB02488_orf45	303	TetR family transcriptional regulator	HY68_35045 (KIF67318)	75/82
CB02488_orf46	209	TetR family transcriptional regulator	SSTG_04890 (EFF89020)	45/58
CB02488_orf(+1)	384	FAD-dependent oxidoreductase	DT87_08965 (KDQ67345)	82/89

[a]*orf(-1)* and *orf(+1)* are predicted to represent the upstream and downstream boundaries of the enediyne gene cluster.
[b]Number of amino acids.
[c]Also see Figure 4 and Figure 2C for the organization of the enediyne gene cluster.

Table S4-26. Related to Figures 2, 4. Predicted functions of ORFs in the enediyne biosynthetic gene cluster from *Kitasatospora* sp. CB01950

gene[a]	aa[b]	putative function[c]	protein homologue	% identity/ % similarity
CB01950_orf(-1)	115	Hypothetical protein	BN159_0732 (CCK25111)	61/76
CB01950_orf1	187	Copper resistance protein	CopC (WP_036496954)	36/49
CB01950_orf2	196	Unknown	CalU21 (AAM70363)	65/77
CB01950_orf3	419	PEP-utilizing protein	STTU_0583 (EGJ73372)	71/79

CB01950_orf4	615	Hypothetical protein	SGR_597 (BAG17426)	63/73
CB01950_orf5	160	Hypothetic protein	KSE_07260 (BAJ26566)	62/70
CB01950_orf6	197	TetR family transcriptional regulator	SGLAU_19960 (AIR99946)	40/50
CB01950_orf7	461	Dioxygenase	Sim14 (AAL15592)	42/54
CB01950_orf8	213	Transcriptional repressor	CynR2 (AGO97151)	56/72
CB01950_orf9	323	Unknown	NcsORF56 (AAM78025)	57/70
CB01950_E3	306	Unknown	SgcE3 (AAL06702)	53/63
CB01950_E7	466	Cytochrome P450	KedE7 (AFV52138)	62/74
CB01950_E6	192	Flavin reductase	KedE6 (AFV52147)	54/67
CB01950_E10	158	Type II thioesterase	KedE10 (AFV52146)	66/74
CB01950_E	1955	Enediyne polyketide synthase	KedE (AFV52145)	56/67
CB01950_E5	340	Unknown	MdpE5 (AAQ17109)	56/69
CB01950_E4	609	Unknown	KedE4 (AFV52141)	52/67
CB01950_orf17	513	Choline dehydrogenase	CynN2 (AGO97168)	36/51
CB01950_orf18	211	Peroxidase	Amir_4098 (ACU37954)	60/74
CB01950_orf19	292	Short-chain dehydrogenase	KedU16 ((AFV52172)	31/46
CB01950_M	344	Unknown	MdpM (ABY66000)	50/60
CB01950_orf21	258	AraC family regulatory protein	SgcR2 (AAL06696)	39/55
CB01950_orf22	248	Hypothetical protein	SPW_3192 (EHM28407)	50/63
CB01950_orf23	368	NAD-dependent epimerase/dehydratase	SpoT8 (ABP55146)	53/62
CB01950_orf24	436	Unknown	CalR3 (AAM94780)	45/56
CB01950_E11	262	Unknown	NcsE11 (AAM78004)	49/58
CB01950_orf26	254	AraC family transcriptional regulator	MdpR2 (ABY65996)	41/57
CB01950_orf27	215	Alkyl hydroperoxide reductase	KCH_58900 (KDN82383)	77/88
CB01950_orf28	197	TetR family transcriptional regulator	SHJGH_6827 (AGF66489)	68/79
CB01950_orf29	263	Thioesterase	KedU43 (AFV52205)	45/58
CB01950_orf30	380	Secreted hydrolase	SSTG_05288 (EFF89418)	62/74
CB01950_orf31	151	Hypothetical protein	WP_027652466	56/72
CB01950_orf32	146	AsnC family transcriptional regulator	IQ62_28255 (KFF97904)	88/93
CB01950_orf33	986	Aminotransferase	SBD_3228 (EMF55915)	76/81
CB01950_orf34	444	Amine oxidase	BN977_00656 (CDO05880)	66/75
CB01950_orf35	363	Polyprenyl synthetase	CF54_29540 (EYT79751)	60/71
CB01950_orf36	306	Membrane protein	FM21_09635 (KFG76345)	76/83
CB01950_orf37	444	Sugar ABC transporter substrate-binding protein	DC74_7769 (AIA08187)	71/80
CB01950_orf38	304	Sugar ABC transporter permease protein	DC74_7770 (AIA08188)	70/85
CB01950_orf39	274	Sugar ABC transporter permease protein	DC74_7771 (AIA08189)	74/87
CB01950_orf(+1)	238	Hypothetical protein	SSPG_06563 (EFD70923)	30/41

[a]*orf(-1)* and *orf(+1)* are predicted to represent the upstream and downstream boundaries of the enediyne gene cluster.
[b]Number of amino acids.
[c]Also see Figure 4 and Figure 2C for the organization of the enediyne gene cluster.

Table S4-27. Related to Figures 2, 4. Predicted functions of ORFs in the enediyne biosynthetic gene cluster from *Amycolatopsis* sp. CB00013

gene[a]	aa[b]	putative function[c]	protein homologue	% identity/ % similarity
CB00013_orf(-1)	154	ATPase	BB31_21500 (AJK55820)	96/98
CB00013_orf1	483	MFS transporter	NcsA2 (AAM77999)	33/51
CB00013_E1	135	HxlR family transcriptional regulator	SgcE1 (AAL06704)	30/53
CB00013_orf3	324	Transcriptional regulator	AJAP_30075 (AIG78844)	98/98
CB00013_orf4	462	Amino acid permease	SSAG_01798 (EFE74432)	76/85
CB00013_orf5	454	PLP-dependent decarboxylase	Sros_4546 (ACZ87410)	66/77
CB00013_orf6	175	Transcriptional regulator	ED92_40280 (KFZ76923)	99/99

CB00013_orf7	90	Hypothetical protein	AORI_2868 (AGM05454)	54/66
CB00013_orf8	305	ATP-dependent DNA ligase	AORI_4514 (AGM07098)	59/70
CB00013_orf9	321	DNA polymerase ligase domain protein	AMETH_5252 (AIJ25344)	50/64
CB00013_orf10	131	Glyoxalase	DynE14 (ACB47074)	34/43
CB00013_orf11	280	RNA polymerase sigma 70	BB31_21445 (AJK55809)	92/95
CB00013_orf12	137	Glyoxalase	Ncsorf29 (AAM77998)	30/40
CB00013_orf13	271	Hypothetical protein	AORI_1866 (AGM04454)	88/95
CB00013_orf14	312	Proline iminopeptidase	SFUL_4173 (AGK79074)	62/70
CB00013_orf15	365	Carboxylate-amine ligase	ACPL_3973 (AEV84868)	46/57
CB00013_orf16	201	MerR family transcriptional regulator	BB31_21365 (AJK55795)	80/90
CB00013_orf17	232	AraC family transcriptional regulator	NcsR5 (AAM78008)	49/62
CB00013_E2	331	Unknown	NcsE2 (AAM78016)	57/66
CB00013_orf19	201	Two-component system response regulator	AJAP_30165 (AIG78862)	99/100
CB00013_orf20	259	SARP family regulator	Spoorf26 (ABP55181)	38/47
CB00013_orf21	228	Regulator	CalU8 (AAM94775)	32/50
CB00013_orf22	206	Regulator	MINT15_01030 (AKJ15167)	82/92
CB00013_orf23	775	Two-component system histidine kinase	AORI_1853 (AGM04441)	96/97
CB00013_orf24	202	Two-component system response regulator	AJAP_30190 (AIG78867)	99/100
CB00013_orf25	63	Hypothetical protein	AORI_1851 (AGM04439)	92/95
CB00013_orf26	184	Peroxidase	SgcO (WP_010056303)	36/50
CB00013_orf27	158	Hypothetical protein	AORI_1849 (AGM04437)	94/96
CB00013_orf28	439	Hypothetical protein	ED92_40390 (KFZ76945)	96/98
CB00013_orf29	182	Hypothetical protein	C791_1366 (EMD28159)	97/99
CB00013_orf30	102	Hypothetical protein	AORI_1846 (AGM04434)	96/98
CB00013_orf31	567	Methylmalonyl-CoA decarboxylase	CynORF6 (AGO97150)	69/79
CB00013_E6	190	Flavin reductase	NcsE6 (AAM78010)	46/64
CB00013_E10	146	Type II thioesterase	NcsE10 (AAM78011)	76/85
CB00013_E	1939	Enediyne polyketide synthase	SgcE (AAL06699)	54/66
CB00013_M	316	Unknown	SgcM (AAL06686)	43/50
CB00013_E9	550	Oxidoreductase	NcsE9 (AAM78005)	75/87
CB00013_E8	185	Unknown	NcsE8 (AAM78006)	67/78
CB00013_E7	450	Cytochrome P450	SgcE7 (AAL06697)	59/73
CB00013_E5	331	Unknown	SgcE5 (AAL06700)	67/80
CB00013_E4	654	Unknown	SgcE4 (AAL06701)	57/70
CB00013_E3	313	Unknown	SgcE3 (AAL06702)	59/69
CB00013_orf42	300	AraC family transcriptional regulator	NcsR5 (AAM78008)	39/54
CB00013_orf43	481	Hypothetical protein	C791_1385 (EMD28178)	91/93
CB00013_orf44	756	Hypothetical protein	AORI_1830 (AGM04418)	98/98
CB00013_orf45	279	SARP-family transcriptional regulator	Spoorf26 (ABP55181)	43/58
CB00013_orf46	228	HxlR family transcriptional regulator	CalU8 (AAM94775)	97/99
CB00013_orf47	192	Hypothetical protein	CalU21 (AAM70363)	63/77
CB00013_orf48	444	Copper resistance protein	ED92_40520 (KFZ76966)	97/99
CB00013_orf49	202	Hypothetical protein	ED92_40525 (KFZ76967)	97/98
CB00013_orf(+1)	222	Chromosome segregation protein	MINT15_01030 (KHF45802)	36/59

[a]*orf(-1)* and *orf(+1)* are predicted to represent the upstream and downstream boundaries of the enediyne gene cluster.
[b]Number of amino acids.
[c]Also see Figure 4 and Figure 2C for the organization of the enediyne gene cluster.

Table S4-28. Related to Figures 2, 4. Predicted functions of ORFs in the enediyne biosynthetic gene cluster from *Kitasatospora* sp. CB02056

gene[a]	aa[b]	putative function[c]	protein homologue	% identity/ % similarity
CB02056_orf(-1)	354	Polyprenyl synthetase	SCATT_56750 (AEW98046)	67/76
CB02056_orf1	607	Acylaminoacyl peptidase	BU52_16835 (KES06082)	50/59
CB02056_orf2	665	Radical SAM protein	Strop_2292 (ABP54742)	65/74
CB02056_orf3	238	Hypothetical protein	Strop_2291 (ABP54741)	52/65

CB02056_orf4	399	Cytochrome P450	KedU45 (AFV52207)	46/58
CB02056_orf5	423	Major facilitator transporter	Strop_2289 (ABP54739)	57/68
CB02056_orf6	278	Non-heme chloroperoxidase	CpoT (AAB86626)	87/96
CB02056_orf7	555	Benzoylformate decarboxylase	P354_07875 (EXU91229)	79/86
CB02056_orf8	211	TetR family transcriptional regulator	M271_40285 (AGP59437)	76/88
CB02056_orf9	155	Hypothetical protein	M271_40290 (AGP59438)	74/83
CB02056_orf10	458	Ammonia channel protein	KCH_52200 (KDN83074)	81/88
CB02056_orf11	394	Cytochrome P450	CalE10 (AAM94800)	43/57
CB02056_orf12	341	Sugar epimerase	CyaORF24 (AGO97213)	55/66
CB02056_E6	192	Flavin reductase	KedE6 (AFV52147)	70/79
CB02056_E10	165	Type II thioesterase	NcsE10 (AAM78011)	73/87
CB02056_E	1960	Enediyne polyketide synthase	NcsE (AAM78012)	56/66
CB02056_M	355	Unknown	SgcM (AAL06686)	49/58
CB02056_E9	551	Oxidoreductase	NcsE9 (AAM78005)	79/87
CB02056_E8	189	Unknown	NcsE8 (AAM78006)	66/75
CB02056_orf19	263	Transcription regulatory protein	MdpR2 (ABY65996)	57/69
CB02056_E7	452	Cytochrome P450	SgcE7 (AAL06697)	67/77
CB02056_E3	324	Unknown	SgcE3 (AAL06702)	58/67
CB02056_E2	346	Unknown	SgcE2 (AAL06703)	66/77
CB02056_orf23	243	Hypothetical protein	Caci_6379 (ACU75233)	46/57
CB02056_E5	337	Unknown	SgcE5 (AAL06700)	54/67
CB02056_E4	642	Unknown	SgcE4 (AAM78014)	50/64
CB02056_orf26	339	Zn-dependent alcohol dehydrogenase	MdpC8 (ABY66028)	68/79
CB02056_orf27	410	Cytochrome P450	NcsB3 (AAM77997)	51/67
CB02056_J	137	Unknown	SgcJ (AAL06676)	55/72
CB02056_F	385	Epoxide hydrolase	NcsF2 (AAM78002)	65/74
CB02056_orf30	463	FAD-binding monooxygenase	KedD2 (AFV52150)	56/66
CB02056_E11	267	Unknown	NcsE11 (AAM78004)	60/70
CB02056_orf32	496	MFS transporter	SSCG_04672 (EDY51467)	34/51
CB02056_orf33	226	Transcriptional repressor	CynR2 (AGO97151)	67/77
CB02056_orf34	214	StrG-like protein	SACT1_0764 (EGE40145)	75/86
CB02056_orf35	229	Hypothetical protein	CynORF27 (AGO97171)	55/72
CB02056_orf36	528	Major facilitator superfamily permease	Spoorf15 (ABP55162)	53/67
CB02056_orf37	114	Hypothetical protein	Spoorf12 (ABP55159)	47/63
CB02056_orf38	248	Unknown	Spoorf22 (ABP55176)	40/59
CB02056_orf39	504	Oxidoreductase	SpoT4 (ABP55175)	28/40
CB02056_orf40	462	NDP-hexose 2,3-dehydratase	KedS2 (AFV52155)	56/69
CB02056_orf41	397	Oxidoreductase	SgcL (AAL06685)	54/69
CB02056_orf42	418	Acyl-CoA dehydrogenase	SACT1_0762 (EGE40143)	86/91
CB02056_orf43	373	NDP-deoxyhexose 3-aminotransferase	SgcA4 (AAL06659)	33/48
CB02056_orf44	420	Methyltransferase	MdpA4 (ABY66027)	60/70
CB02056_orf45	199	dTDP-4-dehydrorhamnose 3,5-epimerase	SgcA2 (AAL06668)	44/58
CB02056_orf46	424	RNA polymerase subunit sigma-24	Spoorf11 (ABP55158)	55/65
CB02056_orf47	429	Glycosyltransferase	KedS10 (AFV52161)	52/65
CB02056_orf48	321	Methyltransferase	NanM (AAP42862)	52/69
CB02056_orf49	336	4-Ketoreductase	KedS5 (AFV52185)	48/58
CB02056_orf50	305	Chitinase	ChiIS (BAC57931)	73/81
CB02056_orf51	488	Amino acid transporter	O3I_022290 (AFU02413)	52/68
CB02056_orf52	215	TetR family transcriptional regulator	O3I_022285 (AFU02412)	47/65
CB02056_orf53	392	Aminopeptidase	ED92_16995 (KFZ81945)	71/79
CB02056_orf54	329	Aldo/keto reductase family oxidoreductase	CalS12 (AAM70349)	32/46
CB02056_orf55	315	Sulfate adenylyltransferase subunit 2	SVEN_5915 (CCA59201)	75/83
CB02056_orf56	192	Adenylylsulfate kinase	DF19_35790 (KDN78997)	72/84
CB02056_orf57	231	Hypothetical protein	Mmar10_1833 (ABI66125)	52/66
CB02056_orf58	401	Cytochrome P450	CalE10 (AAM94800)	46/57

CB02056_orf59	72	Ferredoxin	V519_01015 (KEF16309)	49/62
CB02056_orf60	267	Methyltransferase	BN159_7695 (CCK32074)	52/65
CB02056_orf(+1)	292	Short-chain dehydrogenase	ppKF707_2574 (ELS26341)	47/60

[a]*orf(-1)* and *orf(+1)* are predicted to represent the upstream and downstream boundaries of the enediyne gene cluster.
[b]Number of amino acids.
[c]Also see Figure 4 and Figure 2C for the organization of the enediyne gene cluster.

Table S4-29. Related to Figures 2, 4. Predicted functions of ORFs in the enediyne biosynthetic gene cluster from *Nocardiopsis* sp. TSRI0078

gene[a]	aa[b]	putative function[c]	protein homologue	% identity/ % similarity
TSRI0078_orf(-1)	277	DNA polymerase III subunit epsilon	Ndas_4959 (ADH70341)	90/95
TSRI0078_orf1	138	Hypothetical protein	WP_049570898	58/65
TSRI0078_orf2	147	Unknown	SgcT (AAL06706)	37/54
TSRI0078_orf3	199	Unknown	SgcS (AAL06705)	67/77
TSRI0078_E5	332	Unknown	CynE5 (AGO97182)	80/85
TSRI0078_E4	629	Unknown	CynE4 (AGO97181)	81/88
TSRI0078_orf6	473	FAD-binding monooxygenase	MdpL (ABY66029)	56/71
TSRI0078_J	155	Unknown	CyaJ (AGO97200)	60/71
TSRI0078_orf8	270	Hypothetical protein	AMED_5544 (ADJ47302)	55/72
TSRI0078_orf9	444	Glycosyltransferase	CynGT (AGO97161)	48/65
TSRI0078_orf10	386	Sugar aminotransferase	KedS7 (AFV52158)	59/70
TSRI0078_orf11	357	Glucose-1-phosphate thymidylyltransferase	SgcA1 (AAL06657)	53/68
TSRI0078_orf12	437	Glucose 3,4-dehyratase	CynA3 (AGO97166)	69/79
TSRI0078_orf13	449	UDP-glucose 6-dehydrogenase	MdpA2 (ABY66024)	60/71
TSRI0078_orf14	330	Glucuronic acid decarboxylase	MdpA3 (ABY66025)	61/72
TSRI0078_orf15	172	Hypothetical protein	CyaORF12 (AGO97201)	56/69
TSRI0078_J2	151	Unknown	CyaJ3 (AGO97203)	46/62
TSRI0078_J3	155	Unknown	SgcJ (AAL06676)	59/77
TSRI0078_orf18	392	Oxidoreductase	MdpD2 (ABY66021)	61/74
TSRI0078_orf19	523	Oxidoreductase	CyaN2 (AGO97205)	61/76
TSRI0078_orf20	524	Efflux pump transporter	SpoORF15 (ABP55162)	53/66
TSRI0078_orf21	222	Unknown	SpoORF25 (ABP55180)	70/81
TSRI0078_J4	138	Unknown	NcsJ (AAM77985)	45/58
TSRI0078_orf23	632	Hypothetical protein	WP_028647181	63/73
TSRI0078_orf24	124	Hypothetical protein	WP_028647180	63/74
TSRI0078_orf25	1063	Transcriptional regulator	SpoORF26 (ABP55181)	48/60
TSRI0078_E11	265	Unknown	CynE11 (AGO97159)	83/90
TSRI0078_M	349	Unknown	CynM (AGO97158)	70/75
TSRI0078_E9	549	Oxidoreductase	CynE9 (AGO97157)	91/95
TSRI0078_E8	189	Unknown	CynE8 (AGO97156)	76/83
TSRI0078_E7	448	Cytochrome P450	CynE7 (AGO97155)	83/89
TSRI0078_E3	316	Unknown	CynE3 (AGO97154)	74/82
TSRI0078_E2	339	Unknown	CynE2 (AGO97153)	67/76
TSRI0078_orf33	262	AraC family transcription regulator	CynR3 (AGO97152)	62/76
TSRI0078_orf34	223	TetR family transcription regulator	CynR2 (AGO97151)	77/87
TSRI0078_orf35	539	Methylmalonyl-CoA decarboxylase	CynORF6 (AGO97150)	87/91
TSRI0078_E6	176	Flavin reductase	CynE6 (AGO97149)	75/84
TSRI0078_E10	157	Type II thioesterase	CynE10 (AGO97148)	89/90
TSRI0078_E	1930	Enediyne polyketide synthase	CynE (AGO97147)	80/86
TSRI0078_orf39	520	Hydrolase	DynA4 (ACB47068)	32/45
TSRI0078_orf(+1)	390	DNA polymerase III subunit delta	WP_017569352	90/95

[a]*orf(-1)* and *orf(+1)* are predicted to represent the upstream and downstream boundaries of the enediyne gene cluster.
[b]Number of amino acids.
[c]Also see Figure 4 and Figure 2C for the organization of the enediyne gene cluster.
