# Supplementary material for: Strain Prioritization and Genome Mining for Enediyne Natural Products
Source: mBio. 2016 Dec 20;7(6):e02104-16. doi: 10.1128/mBio.02104-16 (PMC5181780; doi:10.1128/mBio.02104-16)
Supplement: Table S5 — 1H and 13C NMR data and other physicochemical data of TNM A and C. [file mbo006163128st5.pdf]

Table S5. Related to Figure 6. ^{1}H (700 MHz) and ^{13}C (175 MHz) NMR data of TNM A and C in acetone-d_6[a] and other physicochemical data supporting their structural assignments

	TNM A		TNM C	
position	δ_C, type	δ_H (*J* in Hz)	δ_C, type	δ_H (*J* in Hz)
1		9.97 (brs)		9.90 (brs)
2	144.8, C		145.0, C	
3	110.8, C		110.5, C	
4	188.5, C		188.5, C	
5	116.9, C		117.6, C	
6	152.8, C		150.9, C	
7	154.5, C		152.8, C	
8	115.8, CH	7.41 (d, 8.4)	120.1, CH	7.27 (d, 8.2)
9	119.7, CH	7.86 (d, 8.4)	120.3, CH	7.81 (d, 8.2)
10	124.6, C		124.4, C	
11	185.5, C		185.7, C	
12	112.6, C		112.4, C	
13	156.4, C		156.2, C	
14	131.6, CH	8.74 (s)	132.4, CH	8.70 (s)
15	135.1, C		135.8, C	
16	64.3, C		67.8, C	
17	63.9, CH	5.43 (brs)	64.1, CH	6.27 (brs)
18	99.9, C		102.3, C	
19	90.0, C		91.7, C	
20	123.2, CH	6.04 (d, 9.9)	124.1, CH	6.06 (d, 10.0)
21	123.5, CH	5.96 (d, 9.9)	122.7, CH	5.94 (d, 10.0)
22	87.7, C		89.3, C	
23	98.2, C		97.6, C	
24	43.7, CH	5.19 (d, 4.4)	44.7, CH	5.41 (d, 4.9)
25	75.9, C		79.1, C	
26	64.7, CH	4.54 (q, 6.5)	76.5, C	
27	20.8, CH_3	1.44 (d, 6.5)	24.9, CH_3	1.66 (s)
28			75.1, CH	4.42 (s)
29			172.8, C	
30			51.7, CH_3	3.76 (s)
6-OH		13.42 (brs)		13.42 (brs)
7-OMe	55.9, CH_3	4.01 (s)		
13-OH		13.69 (brs)		13.68 (brs)

[a]Assignments were based on COSY, HSQC, and HMBC

Other physicochemical data for TNM A and C:

TNM A: $[\alpha]_D^{25}$ + 1300 (c = 0.002, CH_3OH); UV (CH_3OH) λ_{max} (logε) 208 (3.75), 244 (3.95), 261 (3.93), 395 (3.22), 544 (3.62) nm; IR v_{max} 3403, 2960, 2925, 2853, 1635, 1598, 1458, 1374, 1234, 1198, 1095, 803 cm^{-1}; HR-ESI-MS (positive mode) for the $[M + H]^+$ ion at *m/z* 486.1180 (calculated $[M + H]^+$ ion for $C_{27}H_{19}NO_8$ at *m/z* 486.1189); ^{1}H and ^{13}C NMR spectra are provided in Figure S4.

TNM C: $[\alpha]_D^{25}$ + 200 (c = 0.0005, CH_3OH); UV (CH_3OH) λ_{max} (logε) 219 (3.80), 261 (3.86), 395 (3.31), 558 (3.51) nm; IR v_{max} 3359, 2923, 2852, 1661, 1559, 1467, 1235, 1199, 1097, 802, 724 cm^{-1}; HR-ESI-MS (positive mode) for the $[M + H]^+$ ion at *m/z* 560.1187 (calculated $[M + H]^+$ ion for $C_{29}H_{21}NO_{11}$ at *m/z* 560.1193); ^{1}H and ^{13}C NMR spectra are provided in Figure S4.
