# Supplementary material for: Strain Prioritization and Genome Mining for Enediyne Natural Products
Source: mBio. 2016 Dec 20;7(6):e02104-16. doi: 10.1128/mBio.02104-16 (PMC5181780; doi:10.1128/mBio.02104-16)
Supplement: Text S1 — Supplemental Experimental Procedures. Download [file mbo006163128s1.pdf]

Supplemental Experimental Procedures

General. *Escherichia coli* DH5α was used for routine cloning, *E. coli* XL1-Blue MRF for cosmid library construction, *E. coli* BW25113/pIJ790 for λRED-mediated PCR-targeted gene replacement, and *E. coli* ET12567/pUZ8002 was used for intergeneric conjugation (see Table S1 for all plasmids and strains used). *E. coli* carrying plasmids and cosmids were grown in Luria-Bertani (LB) medium at 37 °C for general cloning or at 28 °C for maintaining temperature-sensitive vectors, and were selected with appropriate antibiotics (2). Standard media and protocols were used for *Streptomyces* growth and sporulation (3). Media components and all other chemicals were from standard commercial sources. Restriction endonucleases were purchased from New England Biolabs (NEB). For Southern analysis, digoxigenin labeling of DNA probes, hybridization, and detection were performed according to the protocols suggested by the manufacturer (Roche Diagnostics Corp.). Cosmid libraries of *Streptomyces* sp. CB02366, *Streptomyces* sp. CB03234 and *Streptomyces uncialis* were constructed using standard protocols (2). Screening of the cosmid libraries were performed by PCR using OneTaq® 2X Master Mix with GC buffer (NEB). The sequences of screened cosmids were determined by end-sequencing using M13 universal primers and by comparison with the genome sequences. Primers used in this study are summarized in Table S1.

Optical rotation values were measured using an Autopol IV automatic polarimeter. CD spectra were measured on a Jasco J-815 Circular Dichroism Spectropolarimeter. UV spectra were recorded with a NanoDrop 2000C spectrophotometer (Thermo Scientific). IR spectra were collected with a Spectrum One FT-IR spectrometer (PerkinElmer). All ^{1}H, ^{13}C, and 2D NMR (HSQC, ^{1}H–^{1}H COSY, HMBC, ROESY) spectra were collected with a Bruker Avance III Ultrashield 700 at 700 MHz for ^{1}H and 175 MHz for ^{13}C nuclei. HR-ESI-MS data were acquired on Agilent 6230 TOF LC/MS instrument.

Construction of the Actinomycetes Genomic DNA Library. Genomic DNA (gDNA) was prepared from strains isolated from various unexplored and underexplored ecological niches (8-10). Strains were cultivated in a rich liquid medium (Tryptic Soy Broth) for 2-3 days. gDNA was isolated using the salting out protocol (3), deposited into 96-well plates, and stored at -80 °C.

Real-time PCR Screening of 3,400 Strains for Enediyne Producers. Real-time PCR was performed using an Applied Biosystems 7900HT Fast Real-Time PCR system. Preparation of gDNA arrays and application of real-time PCR followed a published protocol for strain prioritization (8). The following details were designed for identifying enediyne producers. A positive control, with the gDNA of *Streptomyces globisporus* (the C-1027 producer) (5) or *Streptoalloteichus* sp. ATCC53650 (the KED producer) (11) as a template, and a negative control, with no template, were included in each plate. To anticipate any enediyne cluster not having *E5* or *E10* at the immediate upstream or downstream of *E*, we targeted both *E10/E* and *E/E5* regions (12) (Figure 1B) using the E5T3KS-S/E5T3KS-AS and CTE10E7-S/CTE10E7-AS primers (Table S1), respectively, with final concentrations of 100-500 nM. The reaction conditions consisted of a background check at 50 °C for 2 min; initial denaturation at 95 °C for 7 min; 40 cycles of denaturation at 95 °C for 30 s, primer annealing at 60 °C for 15 s, extension at 68 °C for 90-105 s; and melting steps with a ramp rate of 2%. Each melting temperature (T_m) was normalized to a theoretical T_m (91.7 and 93.5 °C for *E/E5* and *E10/E*, respectively). Samples with $T_m \pm 1.8$ °C compared to the positive control were considered hits, and were subsequently confirmed by DNA sequencing (Figure 1C).

Genome Sequencing and Assembly. To verify the identity of hit strains, another round of PCR was performed using the EKSAT-S/EKSAT-AS primers (Table S1) and the gDNA of the hit strains as templates. The primers were designed to probe the conserved residues of ketosynthase (KS) and acyltransferase (AT) domains within the PKSE (Figure 1B). The resultant products were confirmed by DNA sequencing and BLAST analysis. Aiming at dereplicating identical strains and prioritizing strains with better genetic amenability, three housekeeping genes (16S rRNA, *rpoB*, and *trpB*) were amplified (see Table S1 for primers), sequenced, and analyzed using protocols established previously for taxonomic analysis (13). All DNA sequences have been deposited in the NCBI database (Table S2). Phylogenetic analysis of KS-AT amino acid residues was conducted using ClustalX2 (14) and FigTree v1.4 (http://tree.bio.ed.ac.uk/software/figtree) for sequence alignment and phylogenetic tree construction, respectively. The phylogenetic tree was constructed using the Neighbor Joining method and 1000 bootstrap replications, with AziB, an iterative type I PKS from *Streptomyces sahachiroi* (15) as an outgroup (Figure 2).

Genome sequencing of the representative enediyne producers was performed using an Illumina MiSeq sequencer (2 x 300 paired end sequencing) at the Next Generation Sequencing and Microarray Core Facility, The Scripps Research Institute. Read quality filtering was performed using an in-house developed tool. Adapter trimming and de novo assembly was done with CLC Genomics Workbench version 7.5.1 (CLC Bio.) using default settings. The resulting contigs were further extended and joined into a final scaffold by SSPACE version 2.0 (16) using all quality filtered reads. The remaining gaps inside the final scaffold were partially or completely filled using the quality filtered reads by GapFiller version 1.10 (17). The final assembled drafted geneomes consisted of on averaged 30-50 contigs. Draft genome sequences of the selected hits

(under BioProject PRJNA293172) and *S. uncialis* DCA2648 (under BioProject PRJNA286672) reported in this study have been deposited in GenBank with their accession numbers summarized in Table S2.

Enediyne Gene Cluster Annotation and Genome Neighborhood Network. This was done essentially by following the previously published procedures (1, 18). The *pksE* genes in the enediyne biosynthetic gene clusters in the sequenced genomes were located by BLAST using the SgcE sequence as a query. The fragments containing 100 kb downstream and 100 kb upstream of each located *pksE* were extracted and the ORF assignments were performed by combining the ORF Finder from NCBI, FramePlot 4.0beta, and BLAST analysis. Each ORF was evaluated for homology to proteins from the known enediyne biosynthetic gene clusters using the enediyne genome neighborhood network (GNN) (see below). If no homologue was found, then GenBank was used to propose its possible function. The boundaries of each enediyne biosynthetic gene cluster were estimated by bioinformatics analysis. The annotation tables of all the enediyne biosynthetic gene clusters reported in this study were listed in Tables S3 and S4. The C-1027 gene cluster from *Streptomyces* sp. CB02366, the *ucm* gene cluster from *S. uncialis* DCA2648, and the *tnm* gene cluster from *Streptomyces* sp. CB03234, as well as all the other new enediyne gene producer genomes sequenced, were deposited in GenBank with their accession numbers summarized in Table S2.

The genes from the 10 gene clusters reported previously for the known enediynes, and the *ucm* gene cluster and the 31 gene clusters identified in this study (i.e., the 28 distinct gene clusters plus the three homologous gene clusters from the same clade of CB00072) were collected and translated (Figure 2 and Figure S2). As previously described (1, 18), the total combined list of proteins (1959) was used in an all versus all BLAST using an *E* value limit of 1. Before importation into Cytoscape, *E* values were converted from exponential values using –log(*E* value) with *E* values of 0 arbitrarily converted into a value of 200, i.e., a value larger than the largest calculated value. Self-loops and undirected duplicates were deleted, and an *E* value threshold of 10^{-6} was established based on the lowest found similarity between the apoprotein family. Cytoscape v3.0 was used for GNN generation, visualization, and analysis (1, 19). All GNNs were displayed using the "organic" layout with edge widths corresponding to the *E* value between proteins (Figure 4).

Gene Inactivation. Inactivation of selected genes within the cloned enediyne clusters in *Streptomyces* species was performed by gene replacement (3, 4). To inactivate *pksE* in *Streptomyces* sp. CB02366, the 2.6-kb upstream fragment and the 2.7-kb downstream fragment of *pksE* were amplified and cloned into pHJL401T to afford pBS1157 (Table S1). The *oriT* and the apramycin resistance gene cassette *aac(3)IV* was then inserted into the homologous arms of pBS1157 to afford pBS1158 (Table S1). pBS1158 was finally introduced into *Streptomyces* sp. CB02366 by intergeneric conjugation (3). Apramycin-resistant and thiostrepton-sensitive colonies were selected as the desired *ΔpksE* mutant strain, SB1036 (Figure S3-1A), whose genotype was confirmed by PCR (Table S1) and Southern analysis (Figure S3-1B).

To inactivate *ucmE* in *S. uncialis*, the *ucmE* gene, located on cosmid pBS18003, was replaced by the *aac(3)IV-oriT* cassette from pIJ773 via λRED-mediated PCR-targeted gene replacement (4), to yield pBS18004 (Table S1), which was introduced into the *S. uncialis* wild-type strain by intergeneric conjugation as described above. Apramycin-resistant and kanamycin-sensitive exconjugates were similarly selected to isolate the desired *ΔucmE* mutant strain SB18001 (Figure S3-2A). The genotype of SB18001 was confirmed by PCR (Table S1) and Southern analysis (Figure S3-2B).

To inactivate *tnmE* and *tnmH* in *Streptomyces* sp. CB03234, these two genes, located on the cosmid pBS20002, were replaced by the kanamycin resistant gene from pJTU4659 via λRED-mediated PCR-targeted gene replacement (Table S1) (4), to yield pBS20004 and pBS20005 (Table S1), respectively. Following the same procedure as described above, pBS20004 and pBS20005 were introduced into *Streptomyces* sp. CB03234 by intergeneric conjugation. Kanamycin-resistant and apramycin-sensitive exconjugants were selected and named SB20001 (*ΔtnmE*) and SB20002 (*ΔtnmH*), respectively (Figures S3-3A, S3-3C). The genotypes of these mutants were similarly confirmed by PCR (Table S1) and Southern analysis (Figures S3-3B, S3-3D).

Fermentation, Production, and HPLC Analysis of C-1027. The *Streptomyces* sp. CB02366 wild-type and SB1036 (i.e., *ΔpksE*) mutant strains were cultured individually following previously reported procedures (5), with the original C-1027 producer *S. globisporus* wild-type strain as a control. HPLC was carried out on a Beckman ODS column (5 μm, 150 × 4.6 mm, Ultrasphere), eluted isocratically with 20 mM potassium phosphate (pH 6.86):CH_3CN (50:50, v/v) at a flow rate of 1 mL per min and UV detection at 350 nm (Figure 3B). The identity of C-1027 was confirmed by HR-ESI-MS analysis, yielding the $[M + H]^+$ ions at *m/z* 844.2487 and 846.2641, respectively, for C-1027 (calculated $[M + H]^+$ ion for $C_{43}H_{43}N_3O_{13}Cl$ at *m/z* 844.2485) and its aromatized metabolite (calculated $[M + H]^+$ ion for $C_{43}H_{45}N_3O_{13}Cl$ at *m/z* 846.2642), as well as comparison with authentic standards from the known C-1027 producer of *S. globisporus* (Figure 3C) (5). To determine C-1027 titers, HPLC analysis was calibrated with an authentic C-1027 standard (5, 20). The *S. globisporus* wild-

type produces ~ 5.5 mg/L of the C-1027 chromophore, which translates to ~ 74 mg/L of the C-1027 chromoprotein (i.e., the chromoprotein complex consisting of the C-1027 chromophore and the CagA protein) (20). On the basis of the HPLC analysis (Figure 3B), *Streptomyces* sp. CB02366 was estimated to produce the C-1027 chrompotein complex at a titer of ~750 mg/L, which is minimally 10-fold higher than that by the original *S. globisporus* wild-type producer under the same fermentation condition (20).

Fermentation, Production, and HPLC Analysis of UCM. Fermentation of *S. uncialis* and production of UCM was performed on solid agar medium (ISP4, 1 L) following previously published procedures (6). For HPLC analysis, the concentrated crude exact was dissolved in CH_3OH and analyzed on an Agilent Poroshell 120 EC-C18 column (2.7 µm, 50 × 4.6 mm). The column was equilibrated with solvent A (H_2O with 0.1% HCO_2H) and solvent B (CH_3CN) (90:10, v/v), and eluted with a linear gradient (0-15 min, 10-70% B; 15-20 min, 70% B; 20-22 min, 70-100% B; 22 to 25 min, 100% B) at a flow rate of 0.4 mL per min and with UV detection at 539 nm (Figure 5C). The identity of UCM was confirmed by HR-ESI-MS analysis, yielding the $[M + H]^+$ ion at *m/z* 440.1135 (calculated $[M + H]^+$ ion for $C_{26}H_{18}NO_6$ at *m/z* 440.1134) (Figure 6) (6, 21).

Fermentation, Production, and HPLC Analyses of TNM A, B, and C. The *Streptomyces* sp. CB03234 wild-type and the SB20001 (i.e., *ΔtnmE*) and SB20002 (i.e., *ΔtnmH*) mutant strains were cultured individually in 250-mL baffled flasks containing 50 mL of TSB liquid medium. After growth at 28 °C and 250 rpm for 2 days, 5 mL of seed culture were inoculated into 250-mL baffled flasks containing 50 mL of the production medium (1% soluble starch, 0.5% pharmamedia, 0.2% $CaCO_3$, 0.005% $CuSO_4{\cdot}5H_2O$, 0.0005% NaI, pH 7.0). The resulting cultures were incubated at 28 °C and 250 rpm for 7 days and individually harvested. Each culture was centrifuged, the supernatant was extracted with EtOAc, and the cell pellet was extracted with CH_3COCH_3. The combined extracts were concentrated in vacuum and dissolved in CH_3OH for LC-MS analysis. HPLC was carried out on an Agilent Poroshell 120 EC-C18 column (2.7 µm, 50 × 4.6 mm). The column was equilibrated with solvent A (H_2O with 0.1% HCO_2H) and B (CH_3CN) (95:5, v/v), and eluted with a linear gradient (0-18 min, 5-100% B; 18-25 min, 100% B) at a flow rate of 0.4 mL per min and with UV detection at 539 nm (Figure 5D). To determine TNM titers, HPLC analysis was calibrated with authentic TNM standards.

Isolation and Structural Elucidation of TNM A and C. To isolate sufficient quantities of TNM A and C for structural elucidation, large-scale fermentations (6 L each) of *Streptomyces* sp. CB03234 wild-type and the *ΔtnmH* mutant strain SB20002 were carried out in twelve 2-L baffled flasks containing 500 mL of production medium (1% soluble starch, 0.5% pharmamedia, 0.2% $CaCO_3$, 0.005% $CuSO_4{\cdot}H_2O$, 0.0005% NaI, pH 7.0). After fermentation, the culture was centrifuged and the supernatant was extracted with 5 L of EtOAc for three times. The cell pellet was treated with equal volume of CH_3OCH_3, dried in vacuum, and then extracted three times with H_2O: EtOAc (1:1, v/v). The organic phase was combined with the EtOAc phase for the supernatant and concentrated in vacuum to give a crude extract. The crude extract was dissolved in CH_3OH and fractionated by Sephadex LH-20. The fractions with purple color were combined and finally purified by a semipreparative HPLC (C18 column, 5 µm, 250 × 9.4 mm, Agilent) to afford TNM A and C using a 50-min gradient solvent system from 30% CH_3CN in H_2O to 70% CH_3CN in H_2O with a flow rate of 3.2 mL per min. TNM A (1.2 mg) was isolated from 6 L fermentation culture of the *Streptomyces* sp. CB03234 wild-type strain, while TNM C (0.7 mg) was isolated from 6 L fermentation culture of the *ΔtnmH* mutant strain SB20002.

TNM A was isolated as a purple powder. The molecular formula of TNM A was assigned as $C_{27}H_{19}NO_8$ based on HR-ESI-MS analysis, affording an $[M + H]^+$ ion at *m/z* 486.1180 (calculated $[M + H]^+$ ion for $C_{27}H_{19}NO_8$ at *m/z* 486.1189). The ^1H NMR spectrum of TNM A in DMSO-d_6 resembled to that of UCM, suggesting TNM A could be an analogue of UCM (6, 21). To get the well-resolved NMR signals, 1D and 2D NMR data were thus recorded in acetone-d_6 at 700 MHz using a cryoprobe (Table S5). A pair of mutually coupled olefinic protons at δ 5.96 (H-21) and 6.04 (H-20) with a coupling constant of 10 Hz were assigned to be a cis-disubstituted olefin. The ^3J HMBC correlations of H-21 with two quaternary carbons at C-23 (δ 98.2) and C-19 (δ 90.2), and H-20 with the other two quaternary carbons C-22 (δ 87.7) and C-18 (δ 99.9) strongly suggested the presence of an enediyne substructure (Figures 6A, 6B). Further 1D and 2D NMR analysis identified TNM A has the same carbon skeleton as that of UCM (Table S5). Compared to the molecular formula of UCM ($C_{26}H_{17}NO_6$), TNM A could have one extra hydroxyl group and one extra methoxyl group, which were also revealed by the additional proton signal at 4.01 (3H, s, H-28) and 13.42 (1H, brs, 6-OH) and the absence of two aromatic proton in the ^1H NMR of TNM A. To assign the location of the methoxyl and hydroxyl groups, the HMBC correlation of OCH_3/(δ 4.01)/C-7 (δ 154.5); OH (13.42)/C-6 (δ 152.8), C-7 (δ 154.5) and C-5 (δ 116.9), together with a pair of meta-coupled aromatic protons of H-8 (δ 7.41, d, J = 8.4 Hz) and H-9 (δ 7.86, d, J = 8.4 Hz), were observed, indicating the methoxyl and hydroxyl group was substituted at C-7 and C-8, respectively (Figures 6A, 6B).

The molecular formula of TNM C was determined to be $C_{29}H_{21}NO_{11}$ based on HRE-ESI-MS analysis, affording an $[M + H]^+$ ion at *m/z* 560.1187 (calculated $[M + H]^+$ ion for $C_{29}H_{22}NO_{11}$ at *m/z* 560.1193). The enediyne moiety was evidenced by the presence of a pair of cis oriented olefinic protons at δ 6.06 (H-20, *J* = 10.0 Hz) and 5.94 (H-21, *J* = 10.0 Hz), which are correlated with δ 102.3 (C-18), 89.3 (C-22); and 91.3 (C-19) and 97.6 (C-23) in HMBC spectrum, respectively. Three aromatic protons at δ 7.27 (H-8, d, *J* = 8.2 Hz), 7.81 (H-9, d, *J* = 8.2 Hz), and 8.70 (H-14, s) were present in its ^{1}H NMR, similar to those in TNM A, suggesting TNM C has the same substituted pattern in its anthraquinone moiety to that of TNM A (Table S5 and Figures 6A, 6B). The HMBC correlations of H-8/C-6 (δ 150.9), and H-9/C-7 (δ 152.8) indicated two hydroxyl groups were substituted at C-6 and C-7. The finding of the hydroxyl group at C-7 instead of methoxyl group is consistent with the predicted function of methyltransferase encoded by *tnmH* (Table S3). The NH resonance at δ 9.90 showed correlations to C-3 (δ 110.8) and C-15 (δ 135.8); a methine proton at δ 5.19 (H-24) correlated with C-2 (δ 145.0), C-16 (δ 67.8), and C-22 (δ 87.7); and an hydroxylated methine proton at δ 6.27 correlated with C-15 (δ 135.8) and C-19 (δ 91.7) in HMBC spectrum led to the fusion of anthraquinone and enediyne core moieties. The remaining unassigned $C_5H_9O_4$ includes a methyl (δ_{H-27} 1.66, δ_{C-27} 24.9), a methoxyl (δ_{H-30} 3.76, δ_{C-30} 51.7), a methine (δ_H 4.42, δ_C 75.1), a carbonyl carbon (δ_C 172.8), and a hydroxylated quaternary carbon (δ_C 76.9) group. The HMBC correlations observed between H-30 and C-29, and H-27 and C-28 suggested the presence of a methyl 2,3-dihydroxybutanoate side chain, the stereochemistry of which, however, could not be assigned on the basis of the current NMR data alone. Finally, the side chain was attached to C-25 through C-25-C-26 linkage based on the HMBC correlations of H-24 (δ 5.41) with C-26 (76.5), H-28 (δ 4.42) with C-25 (δ 79.1), and CH_3-27 (δ 1.66) with C-25 (δ 79.1) (Table S5 and Figures 6A, 6B).

The similar CD spectra among TNM A and C and the same biosynthetic origin suggested all of them possessed same stereochemistry at C-16, C-17, C-24 and C-25 positions (Figure 6A). The absolute configuration at these positions were finally assigned as 16*S*, 17*R*, 24*S*, and 25*R* on the basis of comparison of their CD spectra with that of an authentic sample of UCM (Figure 6C) (6, 21).

TNM A: $[\alpha]_D^{25}$ + 1300 (c = 0.002, CH_3OH); UV (CH_3OH) λ_{max} (logε) 208 (3.75), 244 (3.95), 261 (3.93), 395 (3.22), 544 (3.62) nm; IR ν_{max} 3403, 2960, 2925, 2853, 1635, 1598, 1458, 1374, 1234, 1198, 1095, 803 cm^{-1}; HR-ESI-MS (positive mode) for the $[M + H]^+$ ion at *m/z* 486.1180 (calculated $[M + H]^+$ ion for $C_{27}H_{19}NO_8$ at *m/z* 486.1189); ^{1}H and ^{13}C NMR data summarized in Table S5 and spectra provided as Figures S4-1 to S4-3.

TNM C: $[\alpha]_D^{25}$ + 200 (c = 0.0005, CH_3OH); UV (CH_3OH) λ_{max} (logε) 219 (3.80), 261 (3.86), 395 (3.31), 558 (3.51) nm; IR ν_{max} 3359, 2923, 2852, 1661, 1559, 1467, 1235, 1199, 1097, 802, 724 cm^{-1}; HR-ESI-MS (positive mode) for the $[M + H]^+$ ion at *m/z* 560.1187 (calculated $[M + H]^+$ ion for $C_{29}H_{21}NO_{11}$ at *m/z* 560.1193); ^{1}H and ^{13}C NMR data summarized in Table S5 and spectra provided as Figures S4-4 to S4-6.

Cytotoxicity Assay of TNMs. The IC_{50}s of TNMs against selected human cancer cell lines, including breast (MDA-MB-468, MDA-MB-231, SKBR-3, KPL-4, BT474, and DYT2), melanoma (M14 and SK-MEL-5), non-small cell lung cancer (NCI-H226), and central nervous system (SF-295 and SF-539), with UCM as a control, were determined as follows. Suspended cultures of cells were diluted to a concentration of 5×10^4 cells per mL in RPMI 1640 medium supplemented with 10% fetal bovine serum, 100 μg per mL of streptomycin, and 100 U per mL of penicillin. The suspended cultures were dispensed into 96-well plates (100 μL per well), and the plates were incubated for 24 hours at 37 °C in an atmosphere of 5% CO_2, 95% air, and 100% humidity. The original medium was then removed, and 100 μL of fresh medium was added, followed by adding serial dilutions of TNM A and UCM (1 μL in DMSO with final concentration ranging from 0 to 100 nM). Plates were incubated under the above conditions for 72 hours. Finally, 20 μL of CellTiter 96® AQueous One Solution Reagent (Promega) was added to the plates and incubation continued at 37 °C in a humidified, 5% CO_2 atmosphere for 30 to 60 min. The absorbance at 490 nm was recorded using an ELISA plate reader. Each point represents the mean ± SD of three replicates, and the IC_{50} was determined by computerized curve fitting using GraphPad Prism (Table 1A).

For comparison of the cell killing rate between TNM A, AFP (22), and AP-3 (see Figure S1C for structures), SK-BR-3 breast cancer cells were incubated with graded doses (ranging from 0 to 100 nM) of drugs for 8, 12, 24, and 72 hours before the assays were developed as described above. Cell viability was expressed as percentage of untreated control cells (Tables 1B, 1C).

Supplemental References

1. **Rudolf JD, Yan X, Shen B.** 2016. Genome neighborhood network reveals insights into enediyne biosynthesis and facilitates prediction and prioritization for discovery. J. Ind. Microbiol. Biotechnol. **43**:261-276.
2. **Sambrook J, Russell DW.** 2001. Molecular Cloning: A Laboratory Manual, 3rd ed (Cold Spring Harbor Laboratory Press, New York).
3. **Kieser T, Bibb MJ, Buttner MJ, Chater KF, Hopwood DA.** 2000. Practical *Streptomyces* genetics (The John Innes Foundation, Norwich, UK).
4. **Gust B, Challis GL, Fowler K, Kieser T, Chater KF.** 2003. PCR-targeted *Streptomyces* gene replacement identifies a protein domain needed for biosynthesis of the sesquiterpene soil odor geosmin. Proc. Natl. Acad. Sci. USA **100**:1541-1546.
5. **Liu W, Christenson SD, Standage S, Shen B.** 2002. Biosynthesis of the enediyne antitumor antibiotic C-1027. Science **297**:1170-1173.
6. **Davies J, Wang H, Taylor T, Warabi K, Huang XH, Andersen RJ.** 2005. Uncialamycin, a new enediyne antibiotic. Org. Lett. **7**:5233-5236.
7. **Larson J, Hershberger C.** 1986. The minimal replicon of a streptomycete plasmid produces an ultrahigh level of plasmid DNA. Plasmid **15**:199-209.
8. **Hindra, Huang T, Yang D, Rudolf JD, Xie P, Xie G, Teng Q, Lohman JR, Zhu X, Huang Y, Zhao LX, Jiang Y, Duan Y, Shen B.** 2014. Strain prioritization for natural product discovery by a high-throughput real-time PCR method. J. Nat. Prod. **77**:2296-2303.
9. **Ma M, Rateb ME, Teng Q, Yang D, Rudolf JD, Zhu X, Huang Y, Zhao LX, Jiang Y, Li X, Rader C, Duan Y1, Shen B.** 2015. Angucyclines and angucyclinones from *Streptomyces* sp. CB01913 featuring C-ring cleavage and expansion. J. Nat. Prod. **78**:2471-2480.
10. **Xie P, Ma M, Rateb ME, Shaaban KA, Yu Z, Huang SX, Zhao LX, Zhu X, Yan Y, Peterson RM, Lohman JR, Yang D, Yin M, Rudolf JD, Jiang Y, Duan Y, Shen B.** 2014. Biosynthetic potential-based strain prioritization for natural product discovery: a showcase for diterpenoid-producing actinomycetes. J. Nat. Prod. **77**:377-387.
11. **Lohman JR, Huang SX, Horsman GP, Dilfer PE, Huang T, Chen Y, Wendt-Pienkowski E, Shen B.** 2013. Cloning and sequencing of the kedarcidin biosynthetic gene cluster from *Streptoalloteichus* sp. ATCC 53650 revealing new insights into biosynthesis of the enediyne family of antitumor antibiotics. Mol. BioSyst. **9**:478-491.
12. **Liu W, Ahlert J, Gao Q, Wendt-Pienkowski E, Shen B, Thorson JS.** 2003. Rapid PCR amplification of minimal enediyne PKS cassette leads to a predictive familial classification model. Proc. Natl. Acad. Sci. USA **100**:11959-11963.
13. **Guo Y, Zheng W, Rong, X, Huang Y.** 2008. A multilocus phylogeny of the *Streptomyces griseus* 16S rRNA gene clade: use of multilocus sequence analysis for streptomycete systematics. Int. J. Syst. Evol. Microbiol. **58**:149-159.
14. **Larkin MA, Blackshields G, Brown NP, Chenna R, McGettigan PA, McWilliam H, Valentin F, Wallace IM, Wilm A, Lopez R, Thompson JD, Gibson TJ, Higgins DG.** 2007. Clustal W and Clustal X version 2.0. Bioinformatics **23**:2947-2948.
15. **Zhao Q, He Q, Ding W, Tang M, Kang Q, Yu Y, Deng W, Zhang Q, Fang J, Tang G, Liu W.** 2008. Characterization of the azinomycin B biosynthetic gene cluster revealing a different iterative type I polyketide synthase for naphthoate biosynthesis. Chem. Biol. **15**:693-705.
16. **Boetzer M, Henkel CV, Jansen HJ, Butler D, Pirovano W.** 2011. Scaffolding pre-assembled contigs using SSPACE. Bioinformatics **27**:578-579.
17. **Boetzer M, Pirovano W.** 2012. Toward almost closed genomes with GapFiller. Genome Biol. **13**:R56.
18. Shen, B. *et al*. 2015. Enediynes: Exploration of microbial genomics to discover new anticancer drug leads. *Bioorg.* Med. Chem. Lett. **25**:9-15.
19. **Shannon P, Markiel A, Ozier O, Baliga NS, Wang JT, Ramage D, Amin N, Schwikowski B, Ideker T.** 2003. Cytoscape: a software environment for integrated models of biomolecular interaction networks. Genome Res. **13**:2498–2504.
20. **Chen Y, Yin M, Horsman GP, Shen B.** 2011. Improvement of the enediyne antitumor antibiotic C-1027 production by manipulating its biosynthetic pathway regulation in *Streptomyces globisporus*. J. Nat. Prod. **74**:420-424.
21. **Nicolaou KC, Chen JS, Zhang H, Montero A.** 2008. Asymmetric synthesis and biological properties of uncialamycin and 26-*epi*-uncialamycin. Angew. Chem. Int. Ed. **47**:185-189.
22. **Law CL, Gordon KA, Toki BE, Yamane AK, Hering MA, Cerveny CG, Petroziello JM, Ryan MC, Smith L, Simon R, Sauter G, Oflazoglu E, Doronina SO, Meyer DL, Francisco JA, Carter P, Senter PD, Copland JA, Wood CG, Wahl AF.** 2006. Lymphocyte activation antigen CD70 expressed by renal cell carcinoma is a potential therapeutic target for anti-CD70 antibody-drug conjugates. Cancer Res. **66**:2328-2337.
