# Supplementary material for: Strain Prioritization and Genome Mining for Enediyne Natural Products
Source: mBio. 2016 Dec 20;7(6):e02104-16. doi: 10.1128/mBio.02104-16 (PMC5181780; doi:10.1128/mBio.02104-16)

Figure S2. Related to Figure 2. A virtual survey of all bacterial genomes available in public databases (1). (A) Alignment of the 10 known enediyne biosynthetic gene clusters highlighting the enediyne PKS gene cassette (i.e., *E10*, *E*, *E5*, *E4*, *and E3*) common to all enediynes. (B) A virtual survey of the GenBank and Joint Genome Institute databases (as of May 2015), with the enediyne PKS cassette as query, yielding 88 enediyne biosynthetic gene clusters, including the 10 known ones, a finding that validates the enediyne PKS cassette as a viable search probe to mine enediyne gene clusters and reveals the rich biosynthetic potential of enediynes by actinomycetes. (C) Taxonomic distribution of all known or potential enediyne producers in public databases with approximately 22% of them belonging to *Streptomyces* species, the most prolific producers of known biologically active natural products.

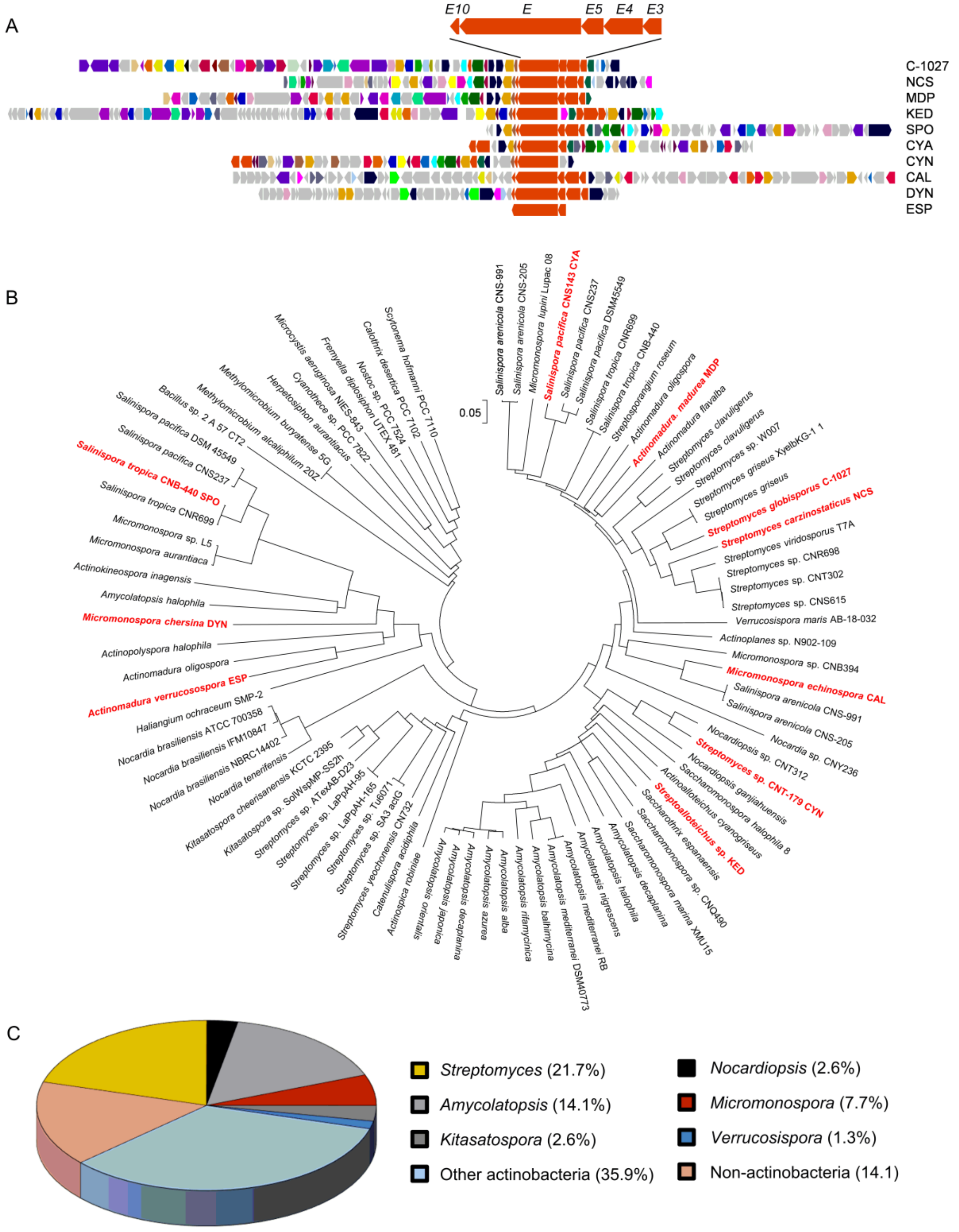

Supplement: Figure S2 — A virtual survey of bacterial genomes for enediyne producers. Download [file mbo006163128sf2.pdf]
